# Supplementary material for: Plant Species as Potential Forage for Honey Bees in the Al-Baha Mountain Region in Southwestern Saudi Arabia
Source: Plants (Basel). 2023 Mar 22;12(6):1402. doi: 10.3390/plants12061402 (PMC10058344; doi:10.3390/plants12061402)

Abutilon pannosum

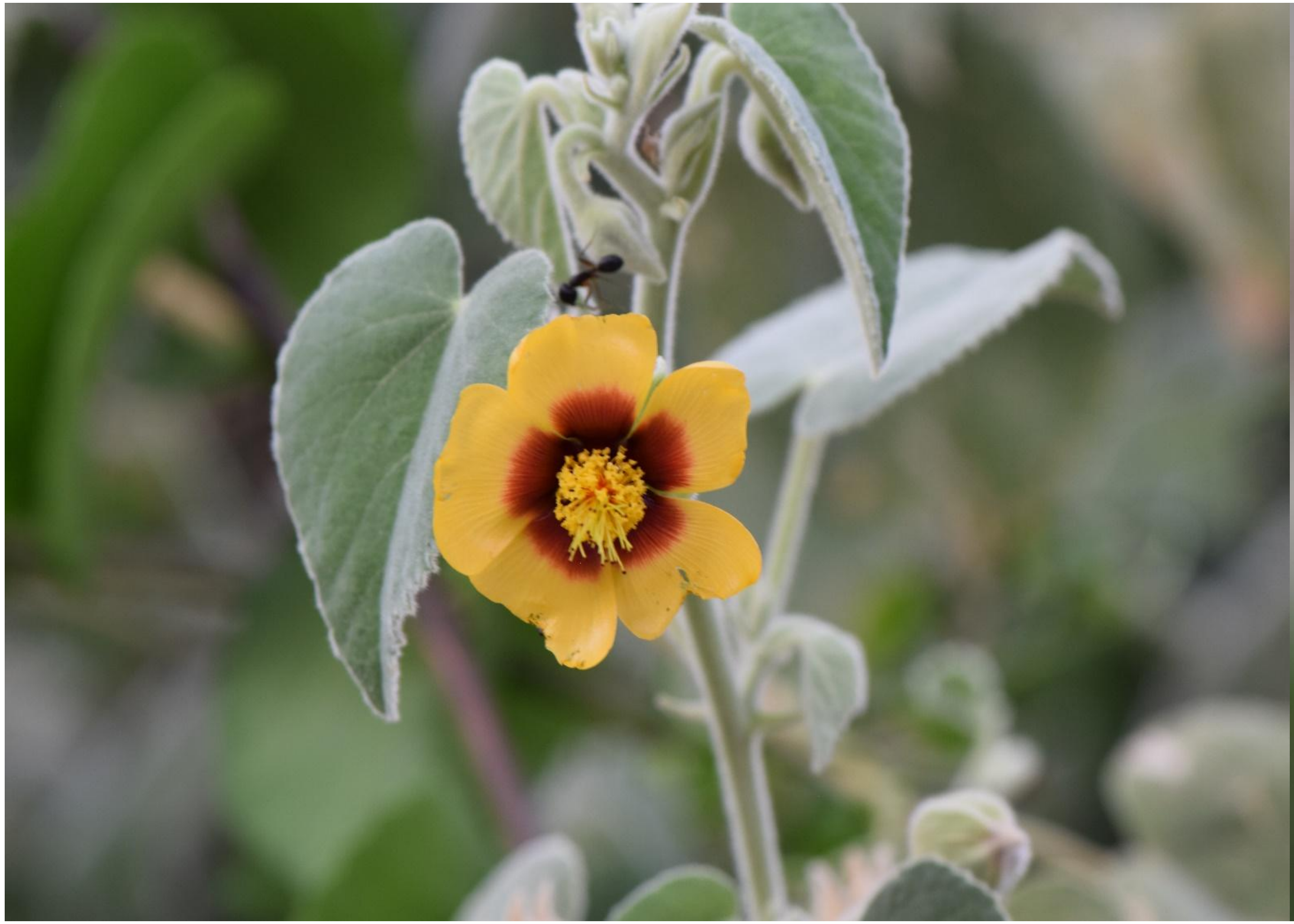
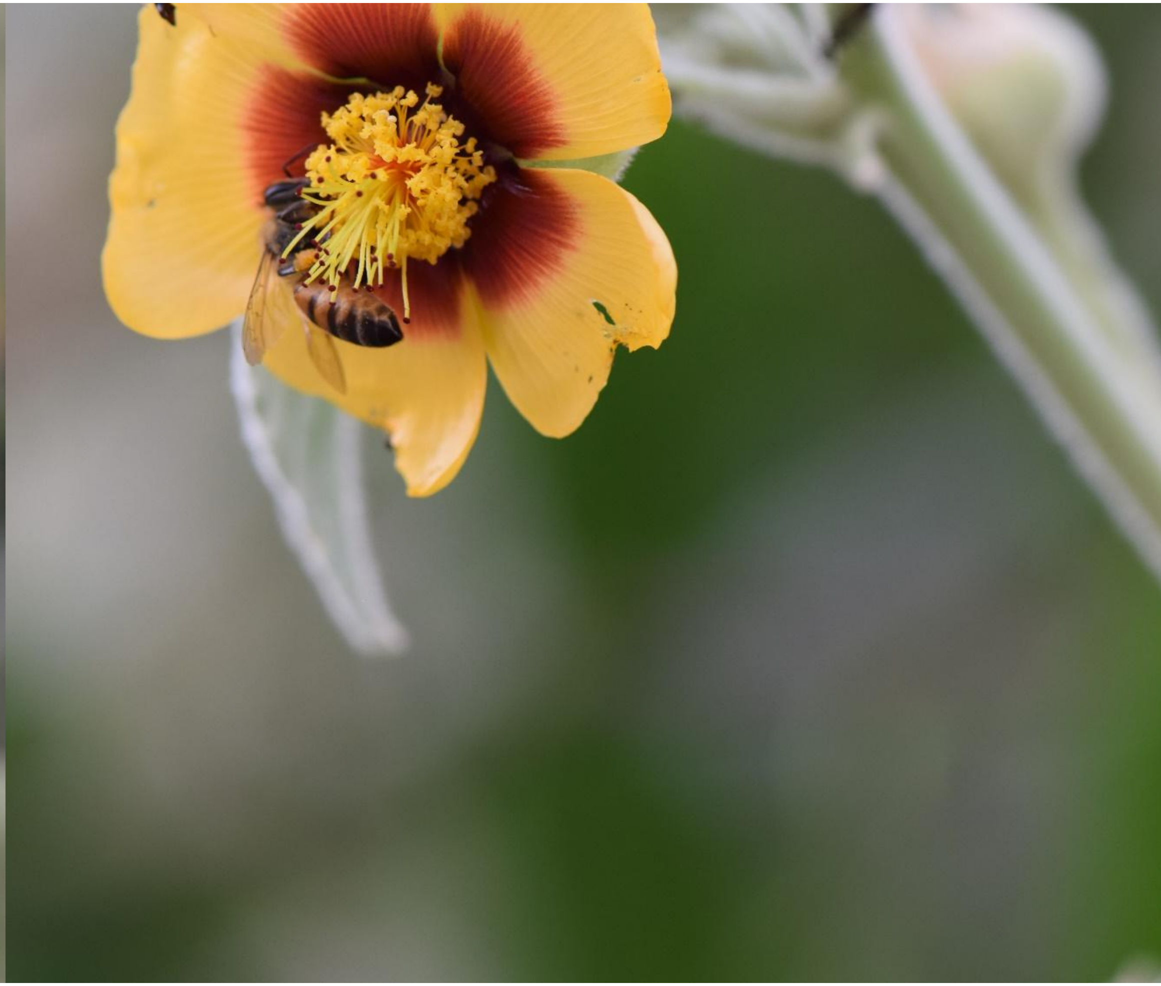

Achyranthes aspera

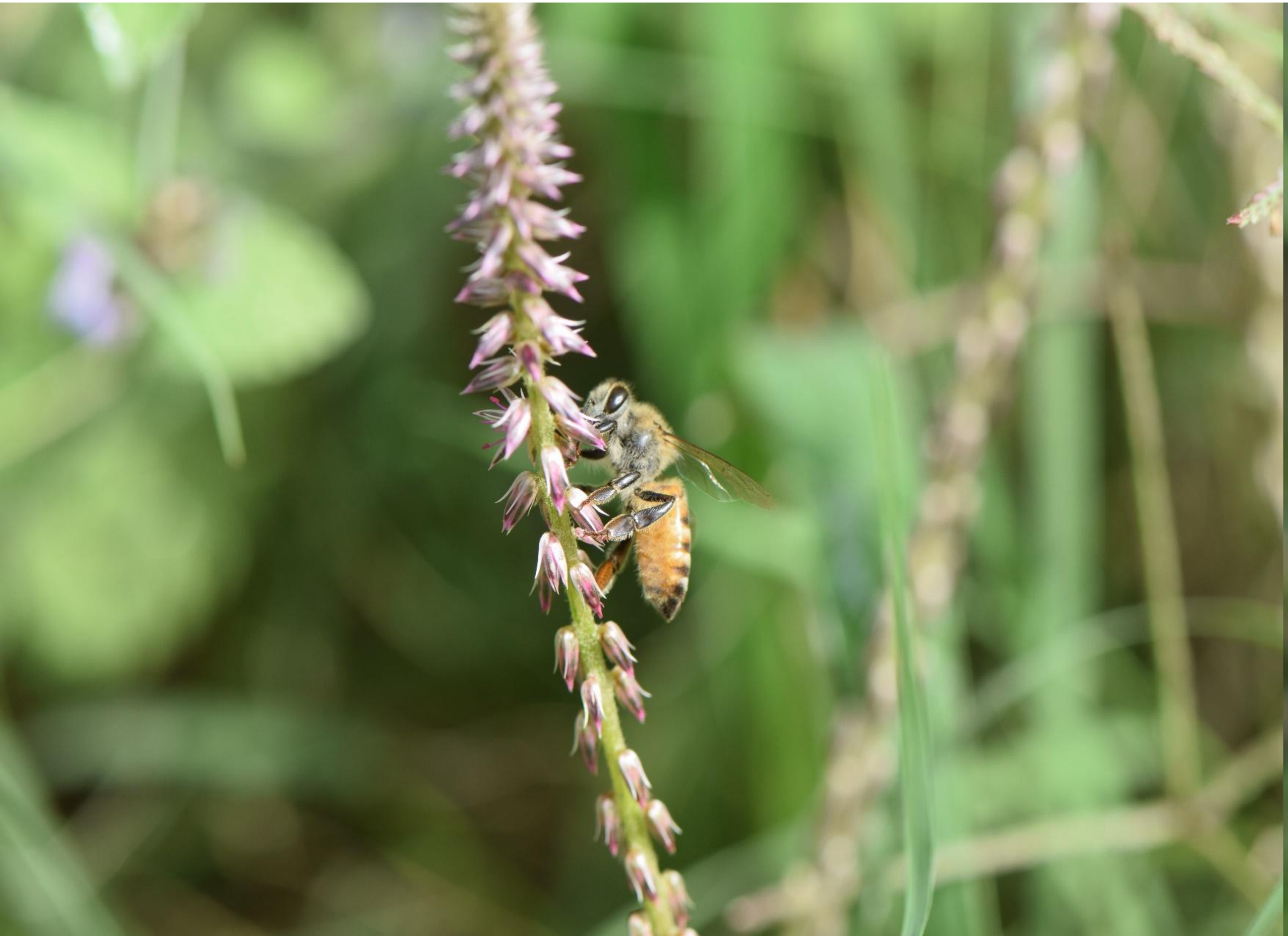

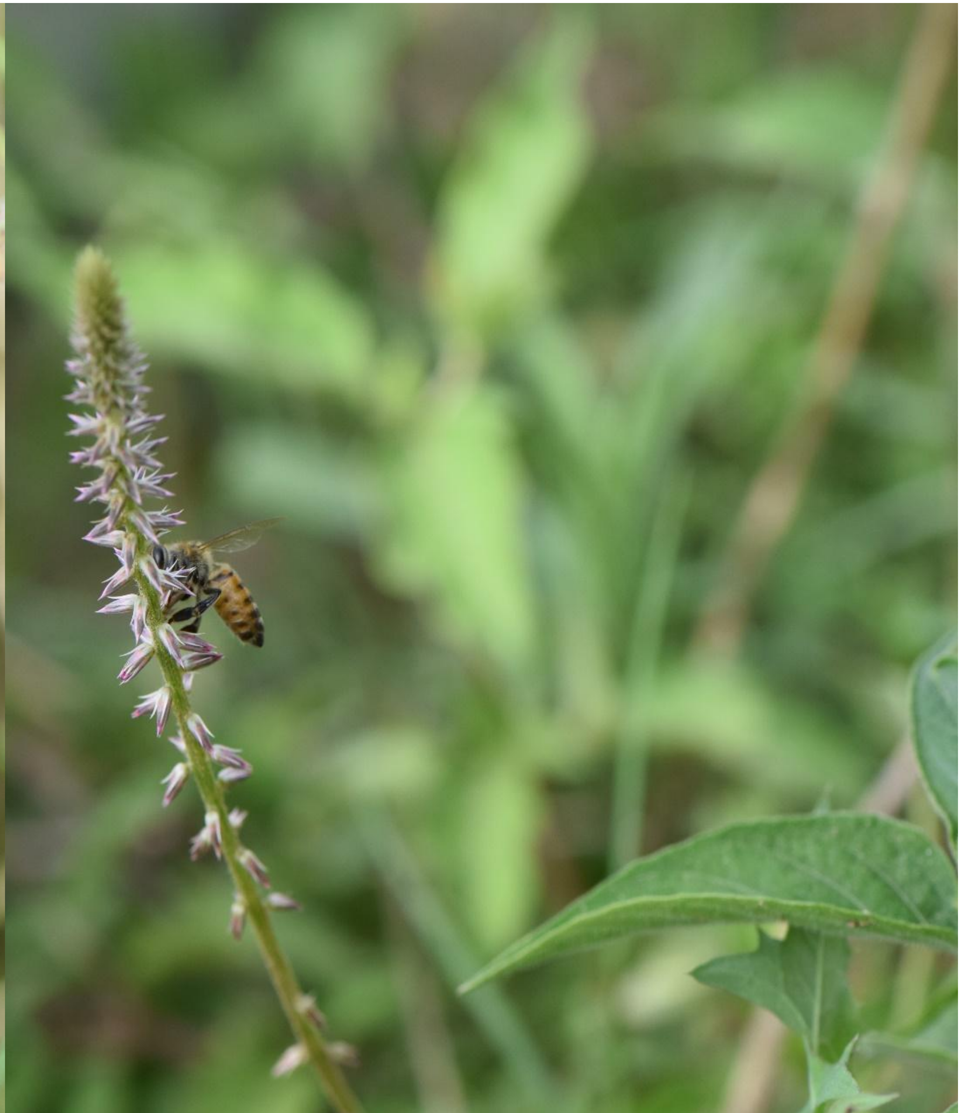

Nepeta deflersiana

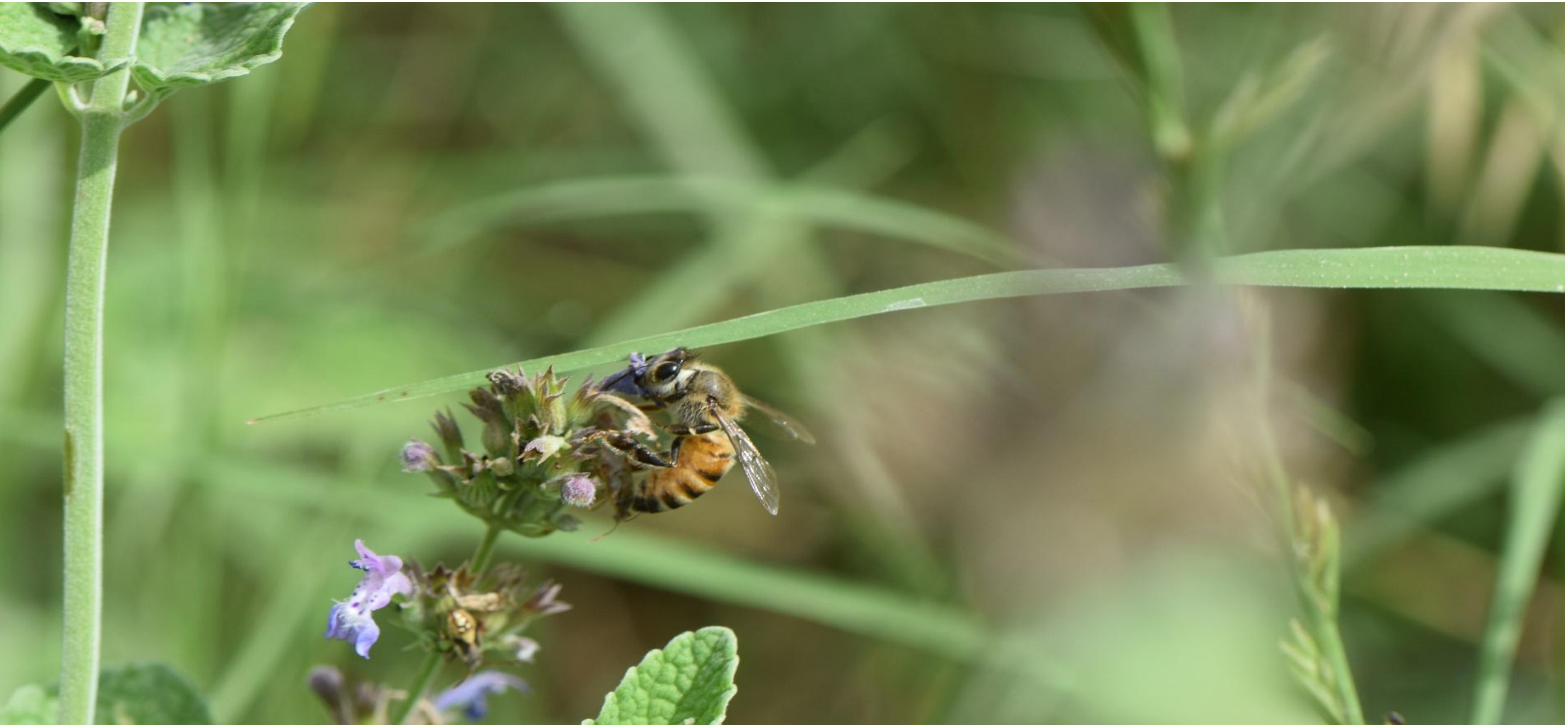

Aloe castellorum

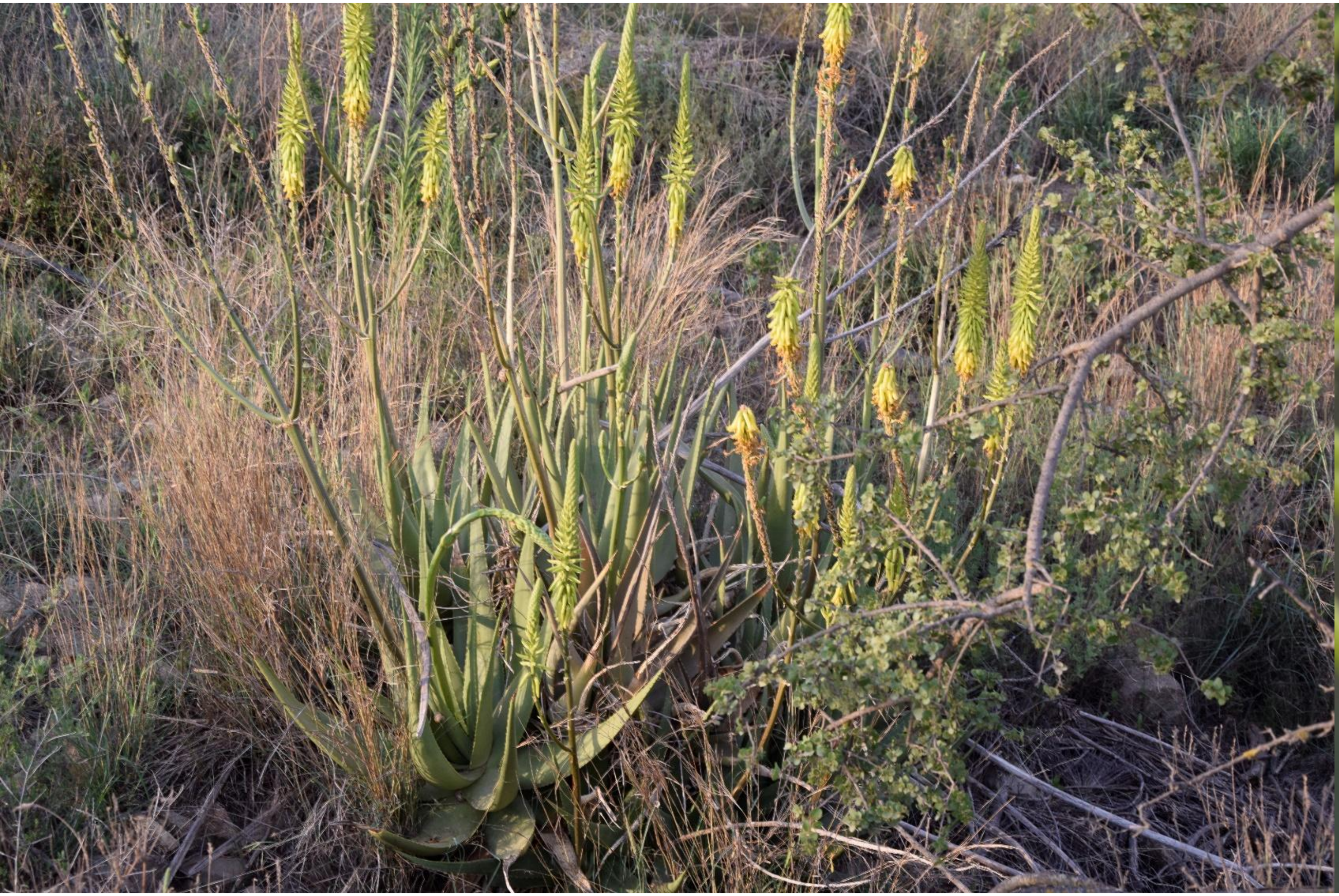

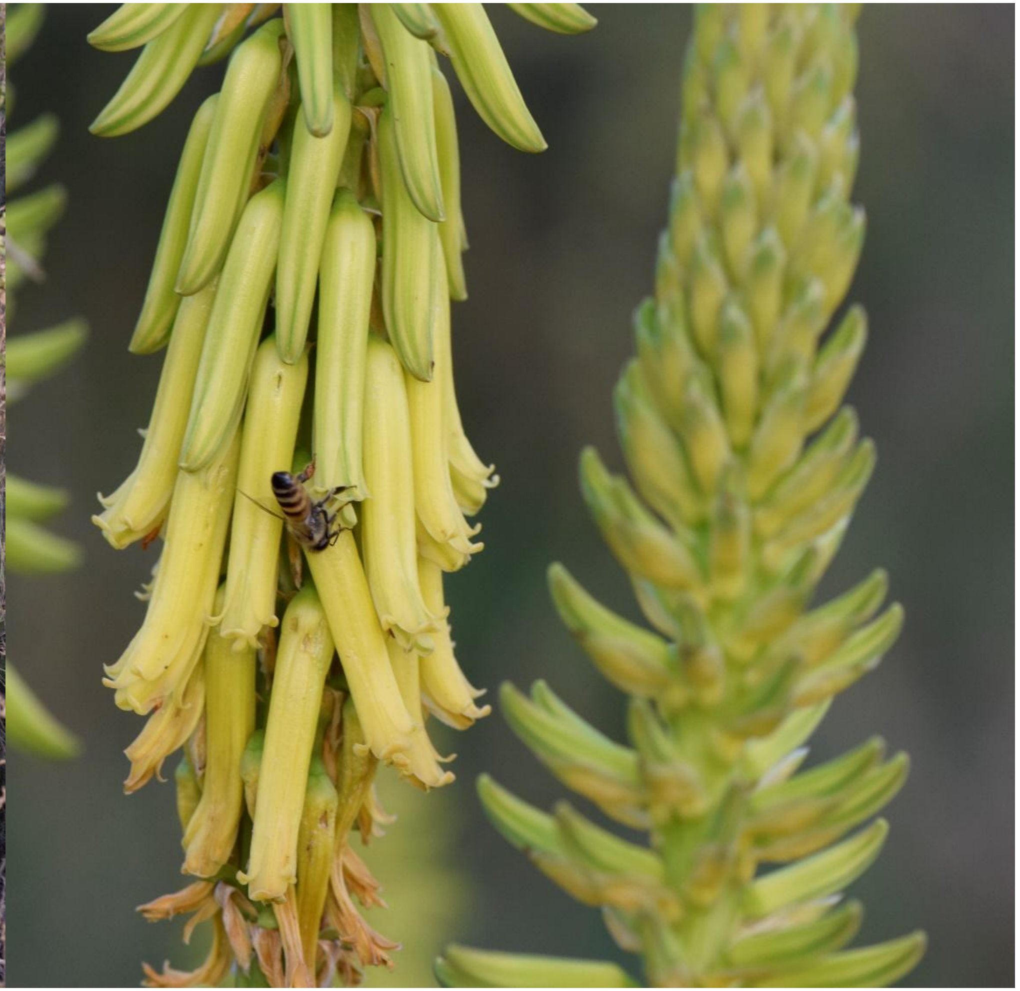

Ammi majus

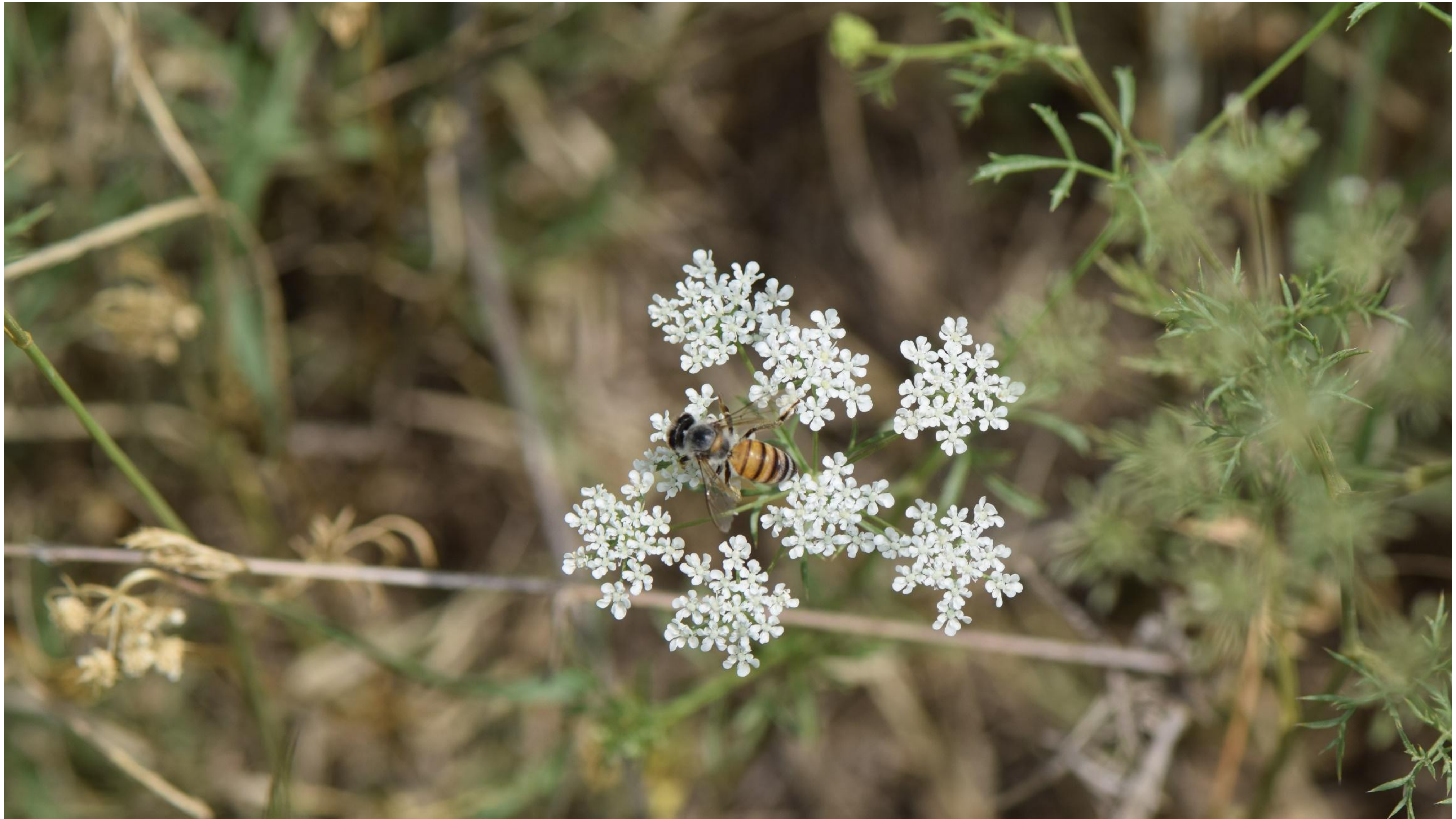

Anarrhinum forsskalii

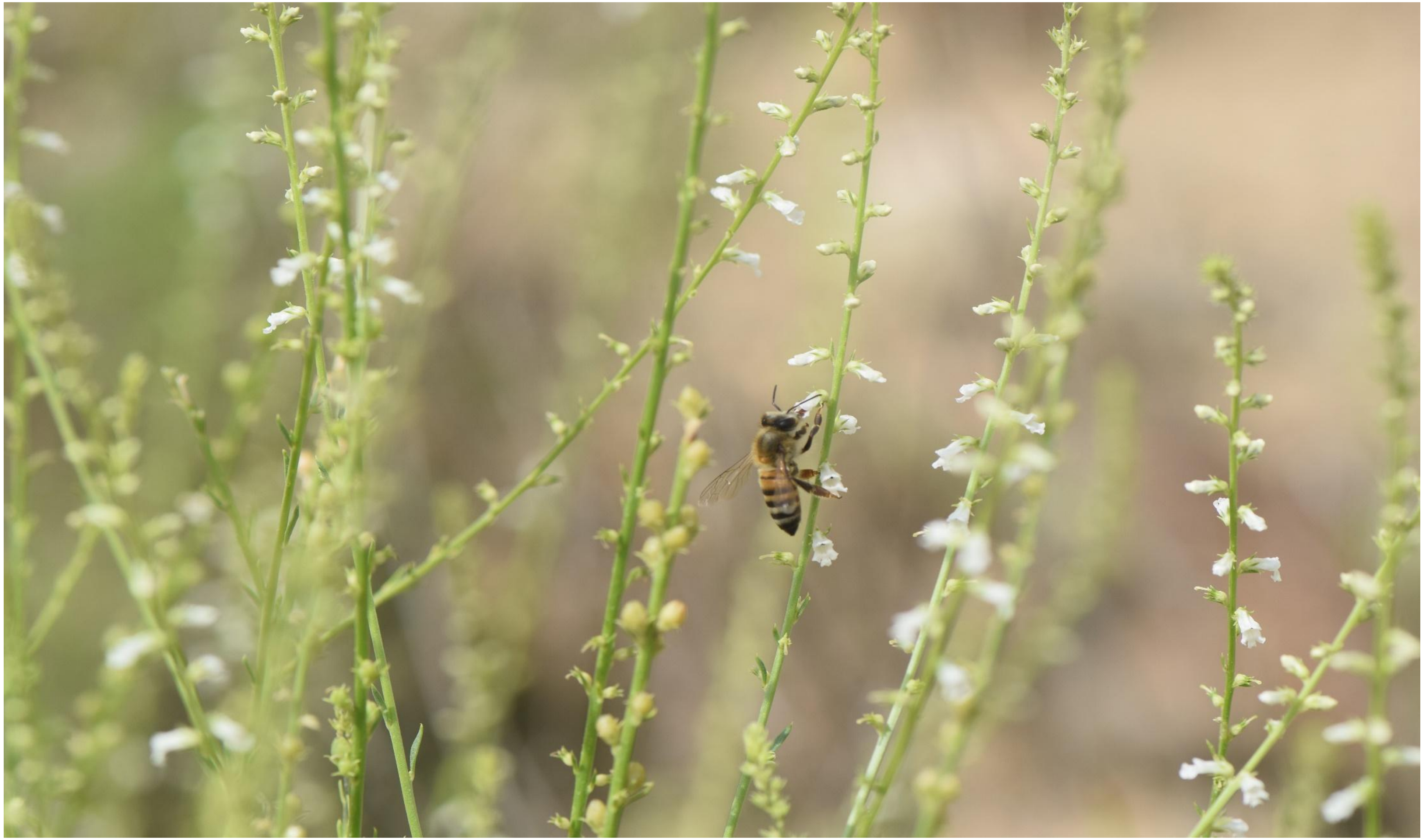

Argemone ochroleuca

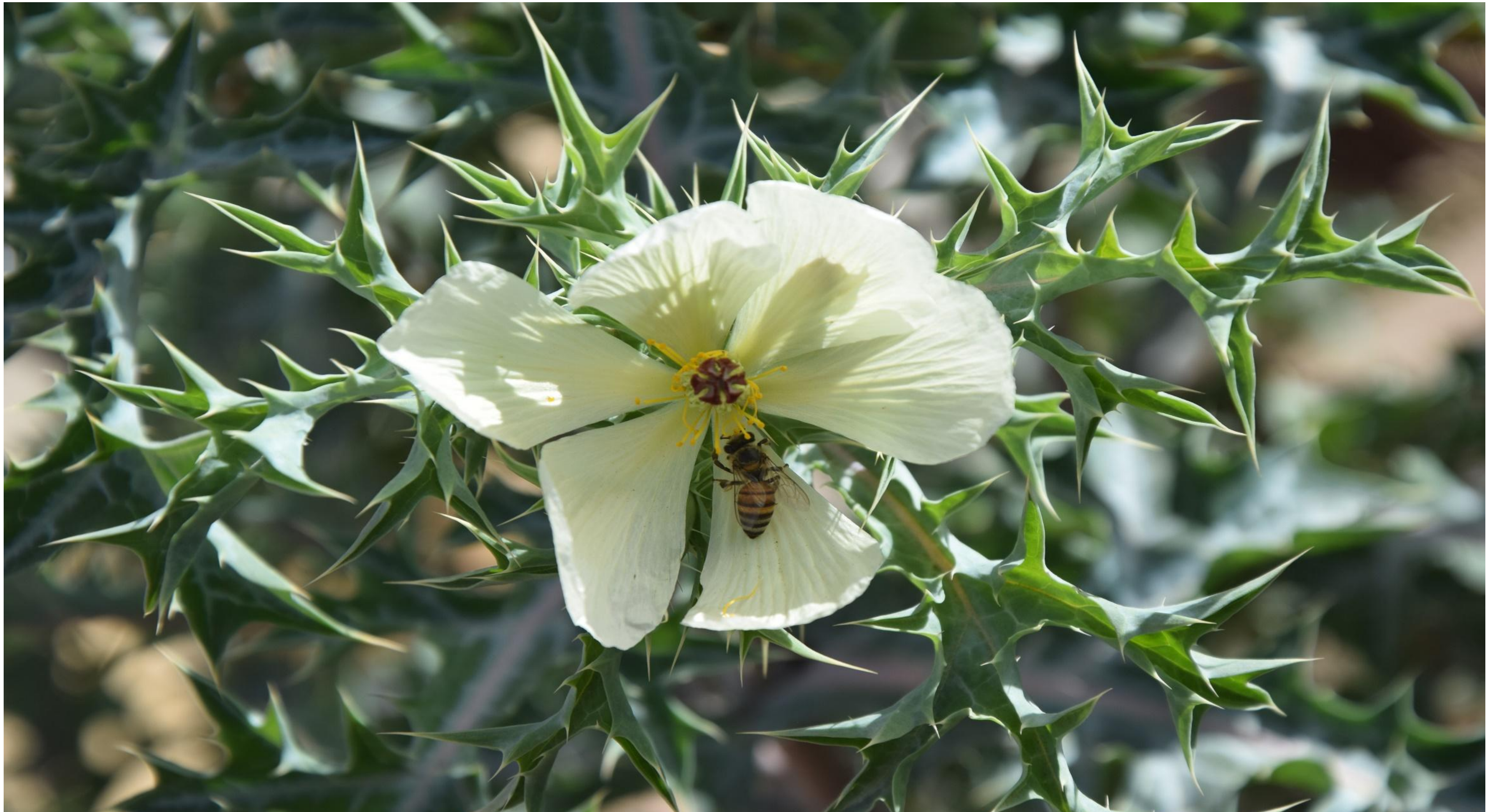

Asphodelus tenuifolius

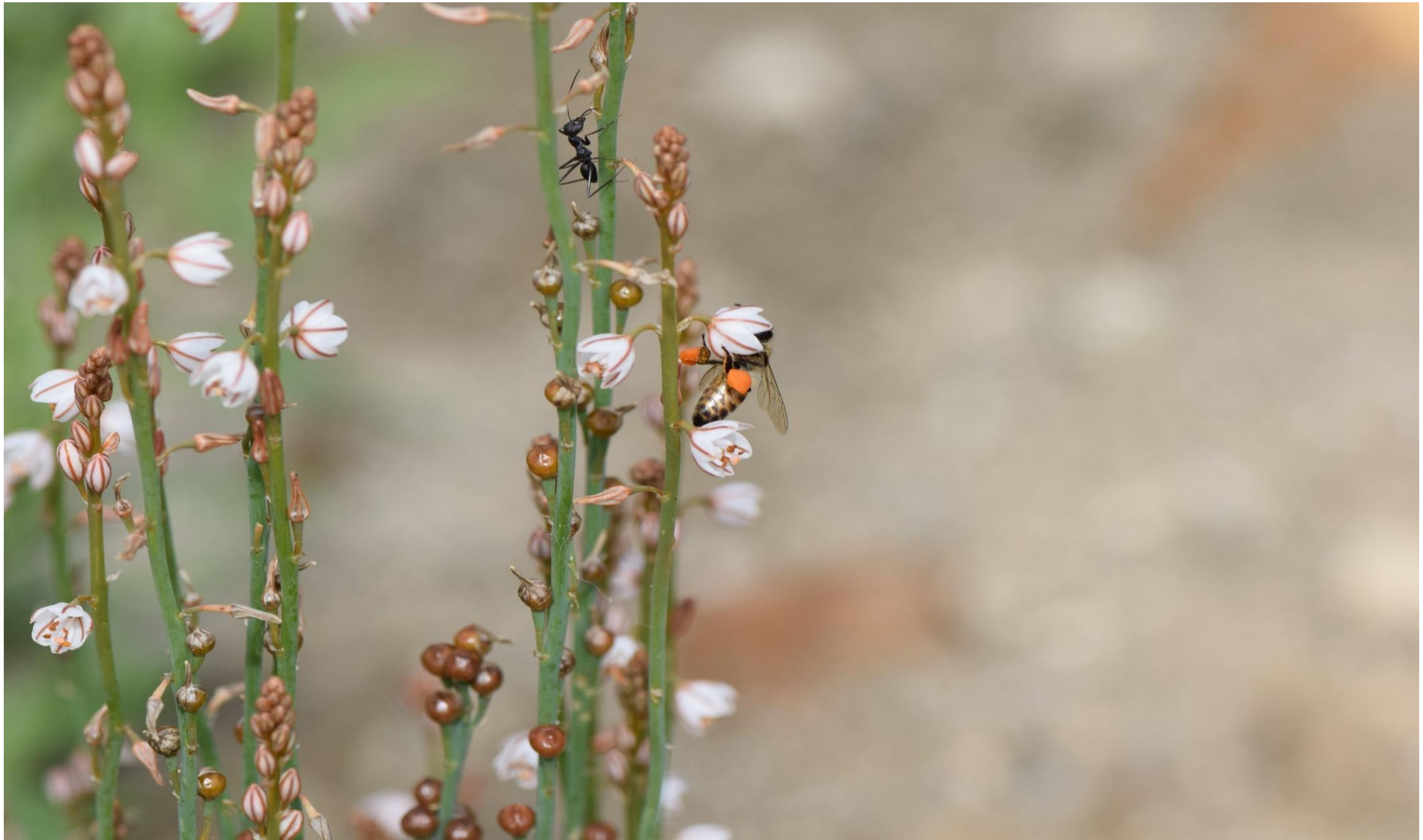

Astragalus abyssinicus

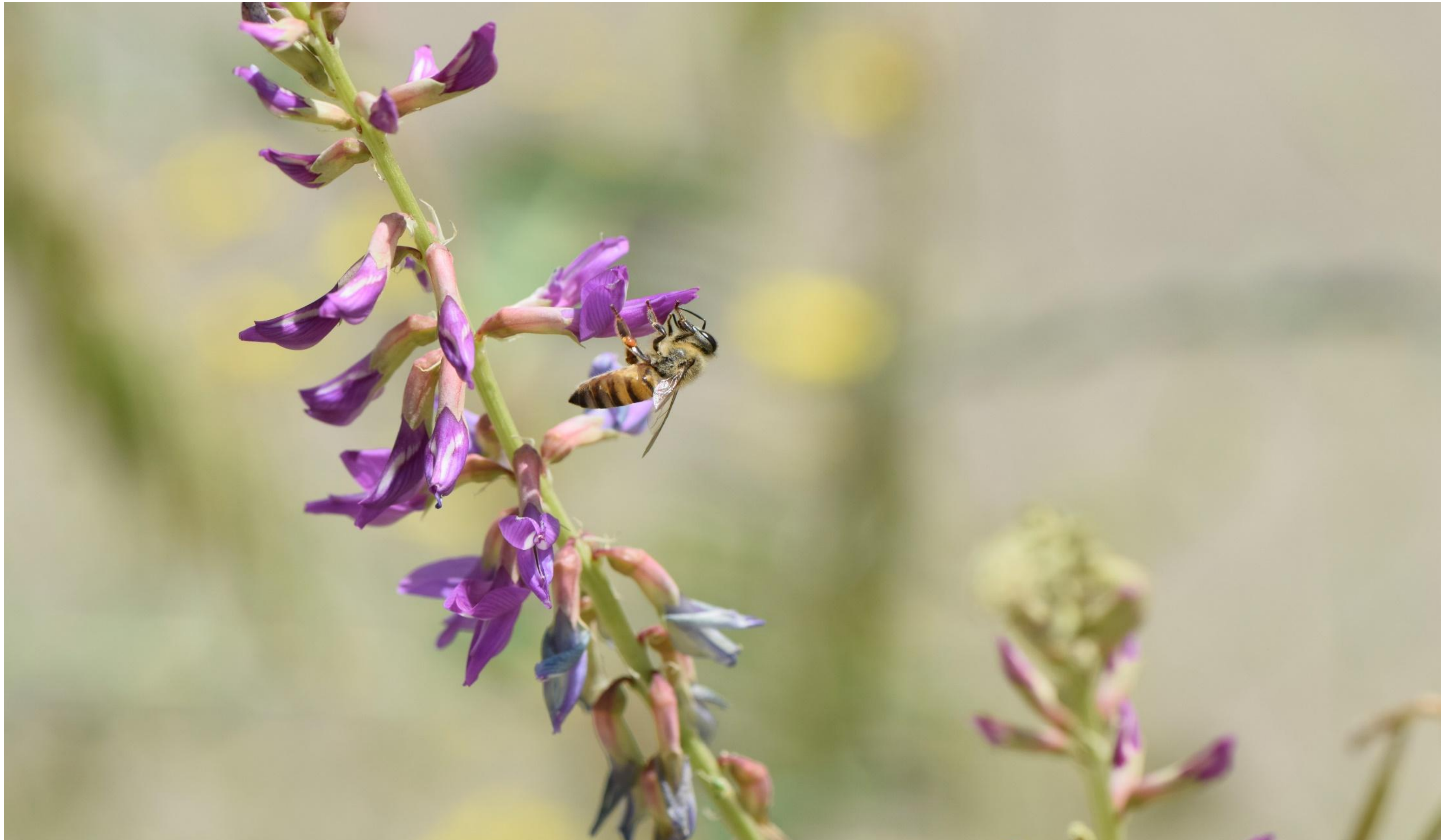

Baccharoides schimperi

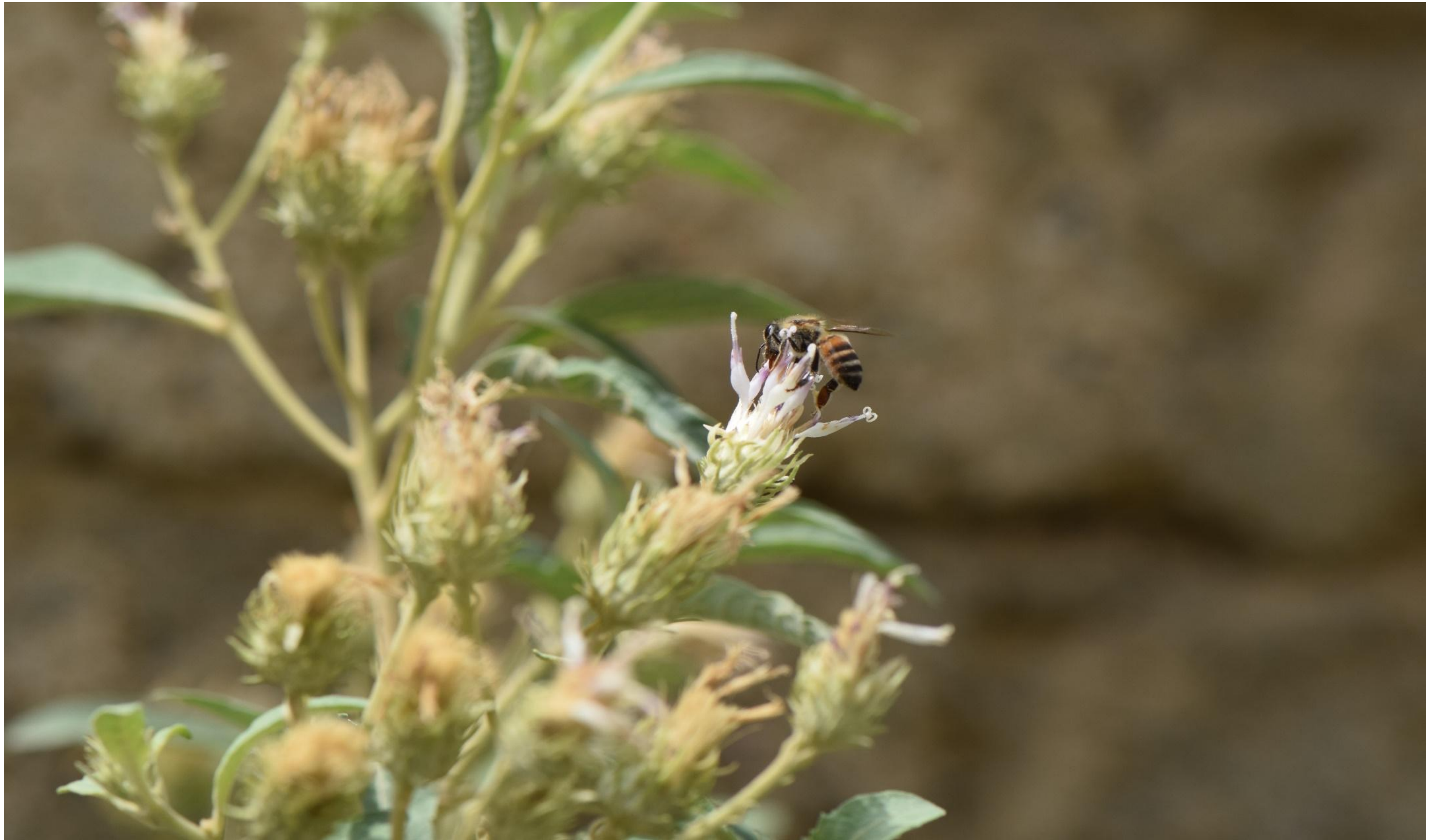

Bacopa monnieri

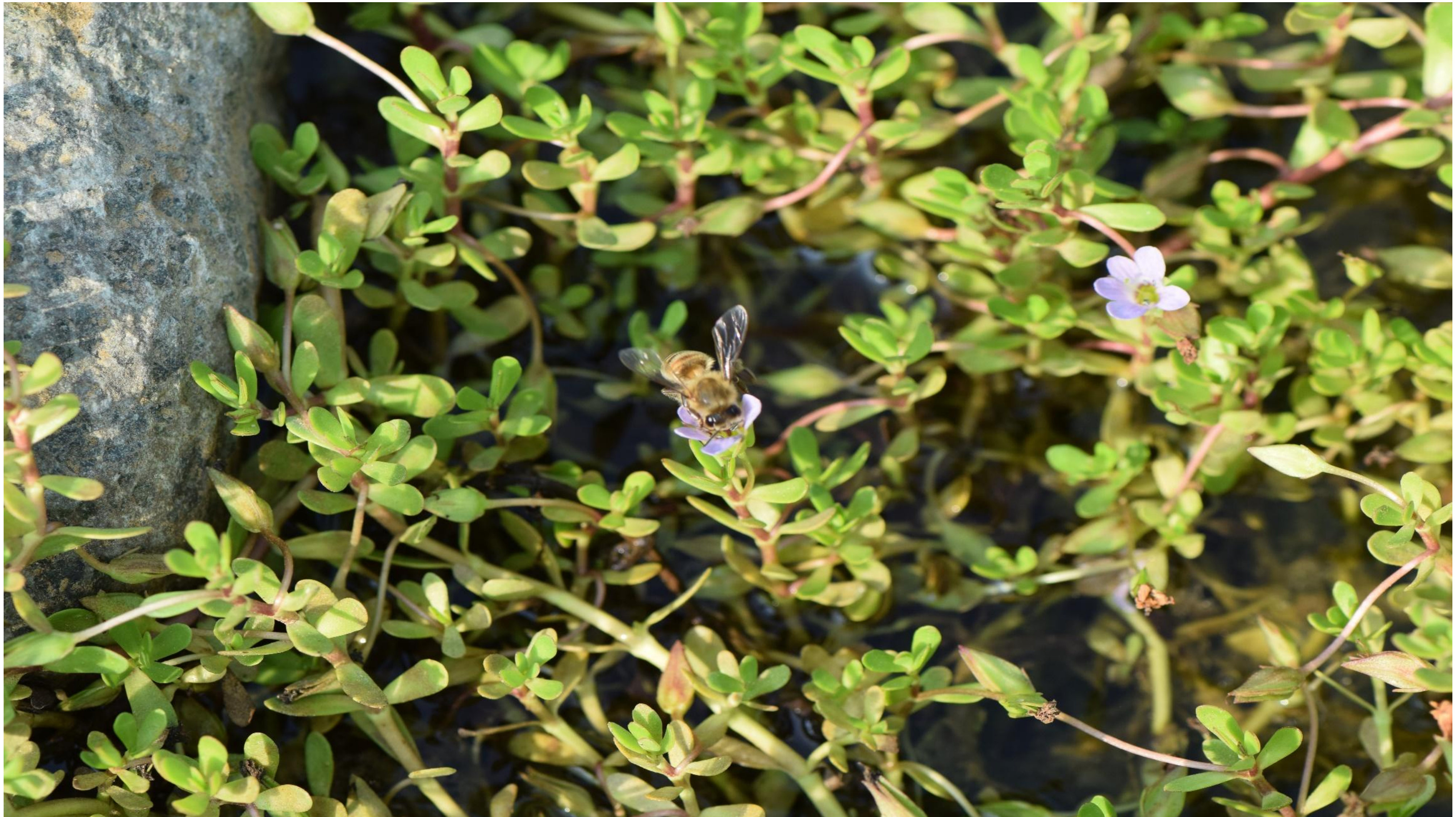

Bidens pilosa

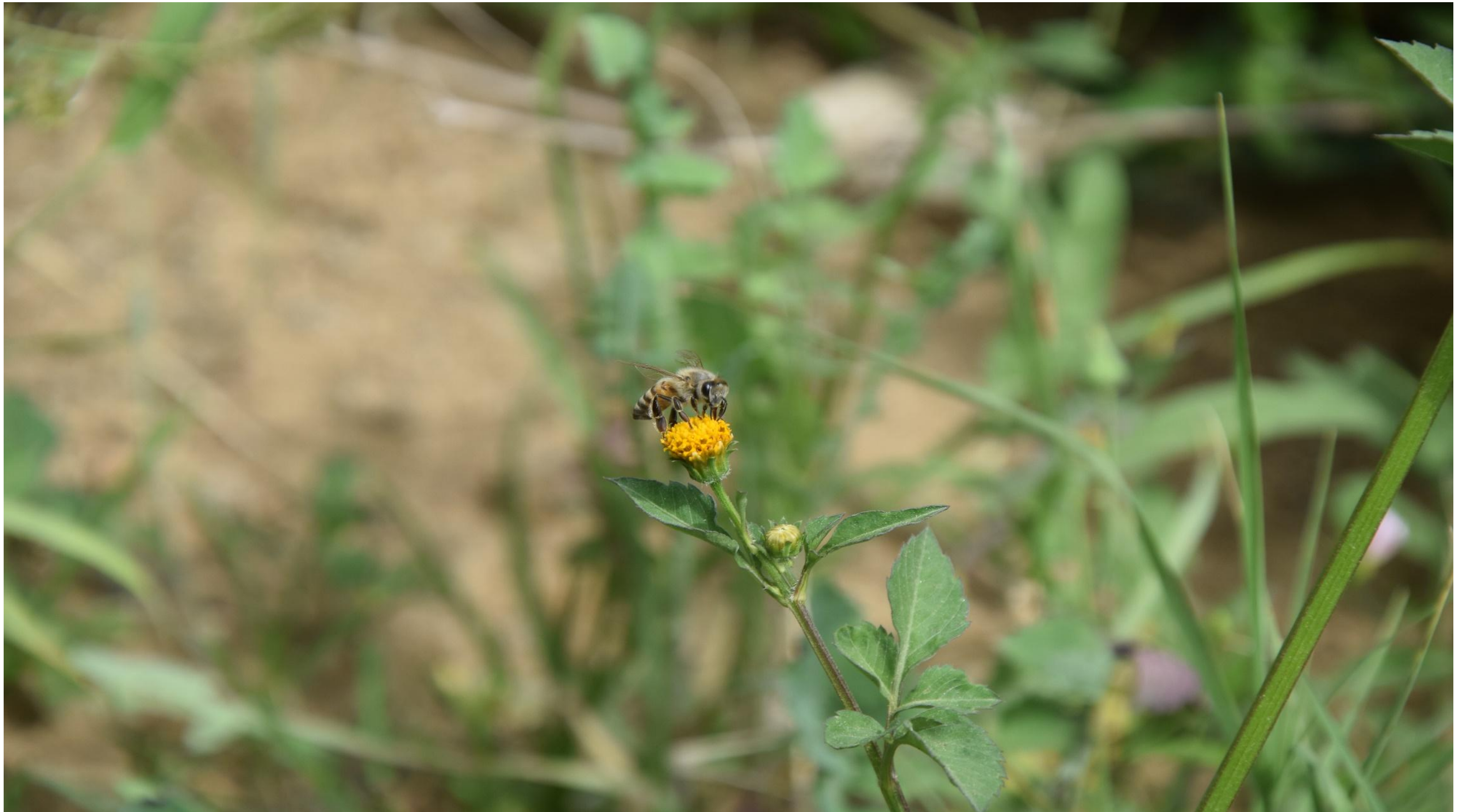

Blepharis edulis

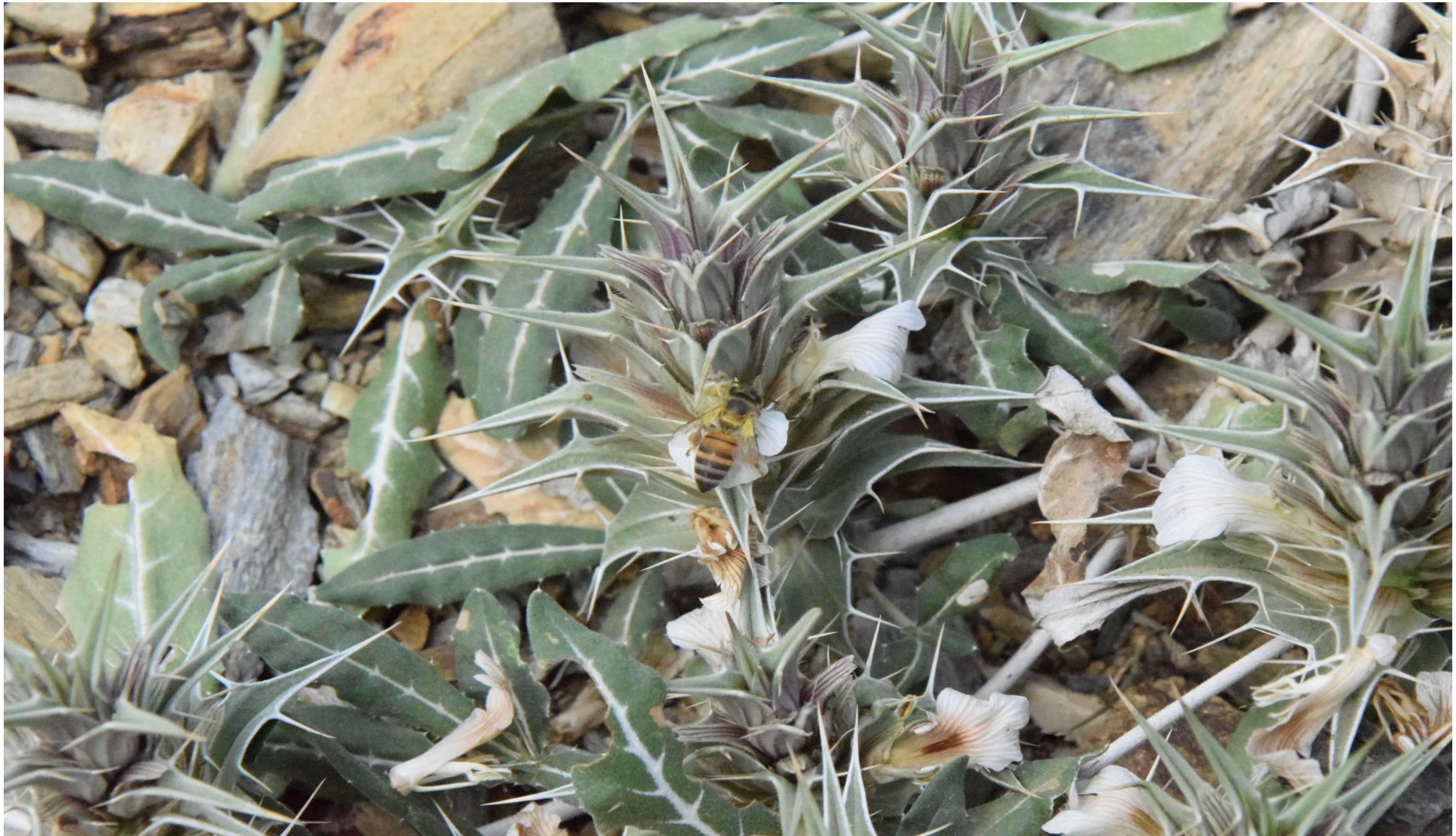

Calatropis procera

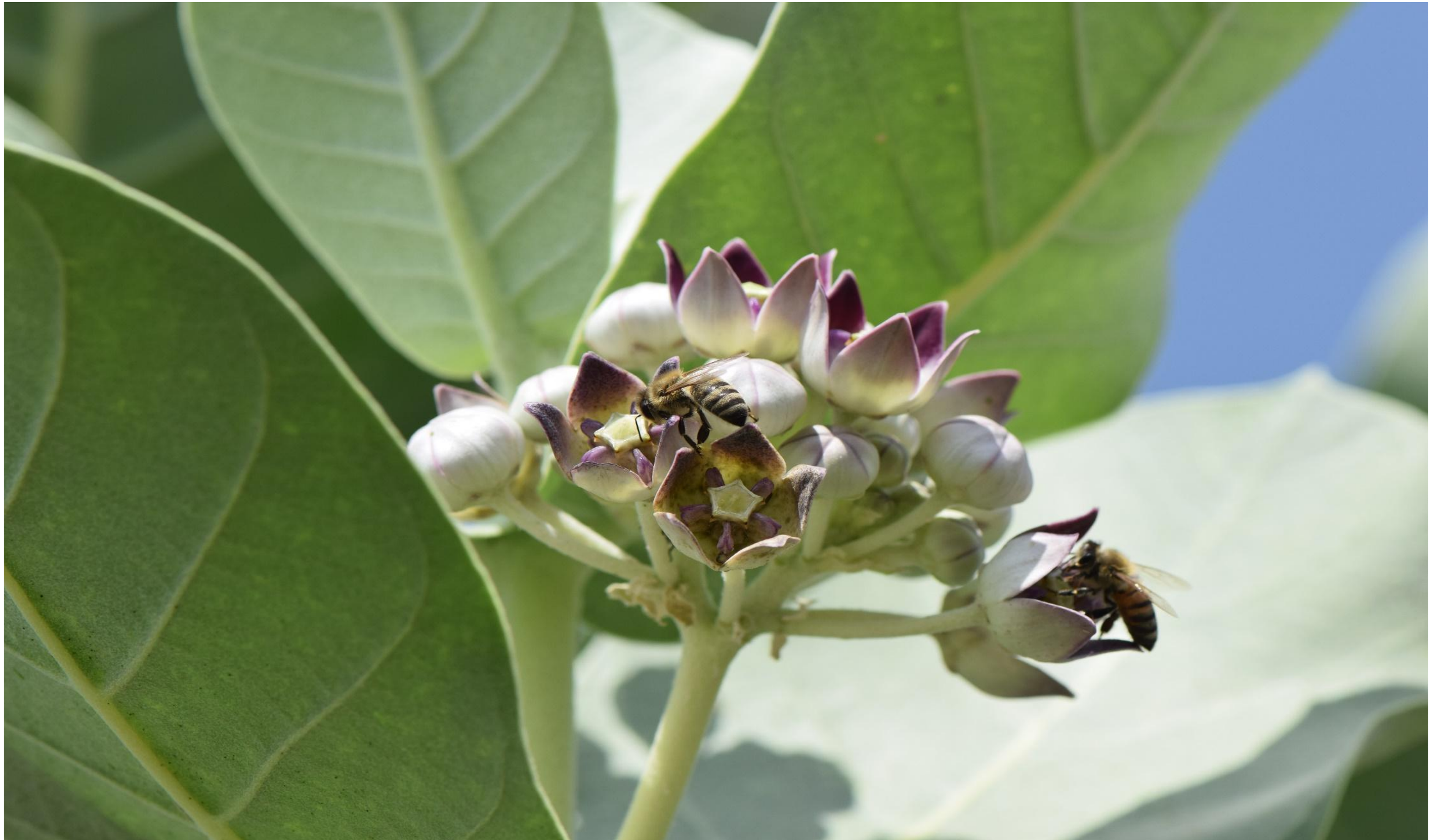

Capparis cartilaginea

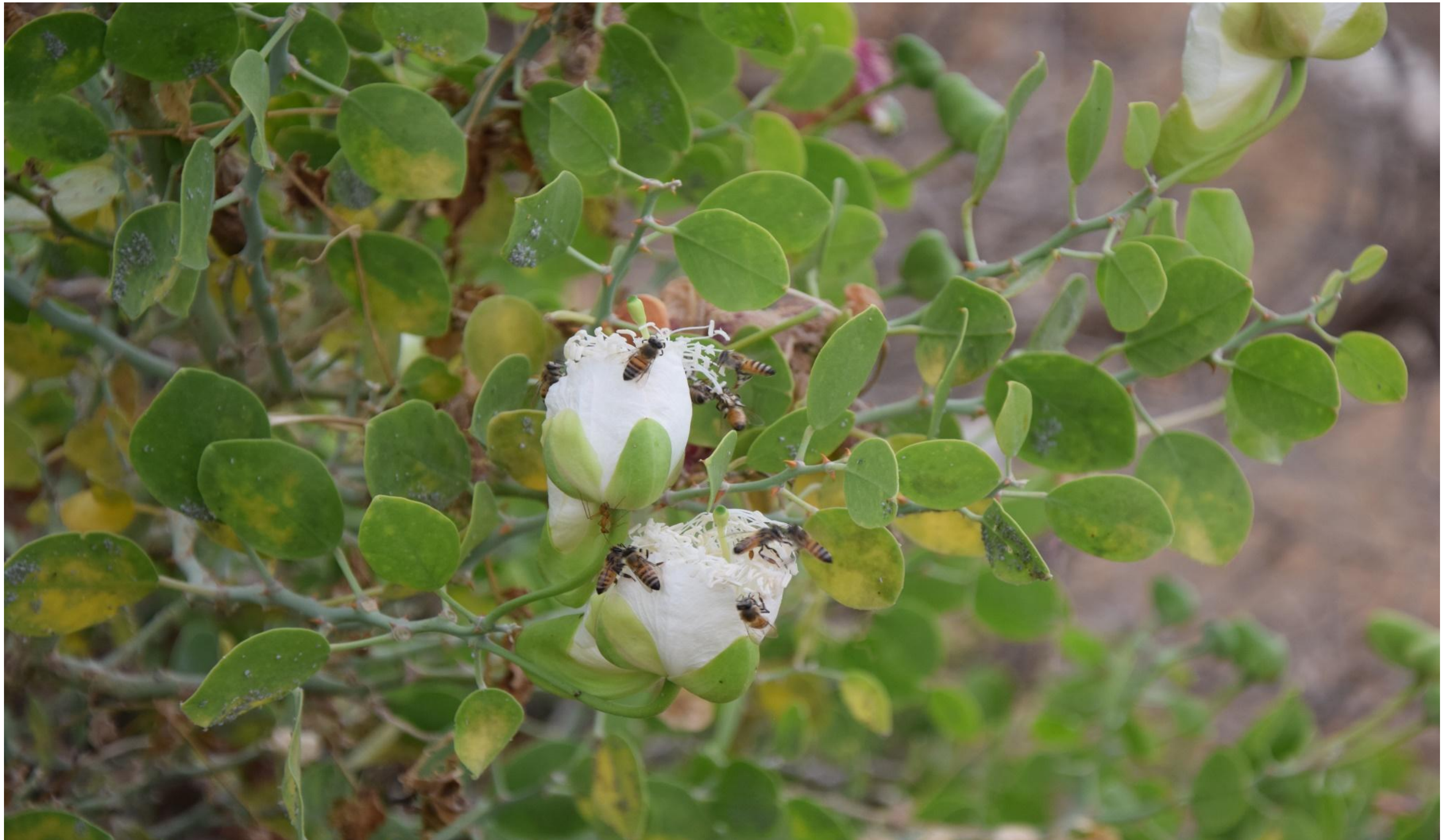

Caylosia hexigina

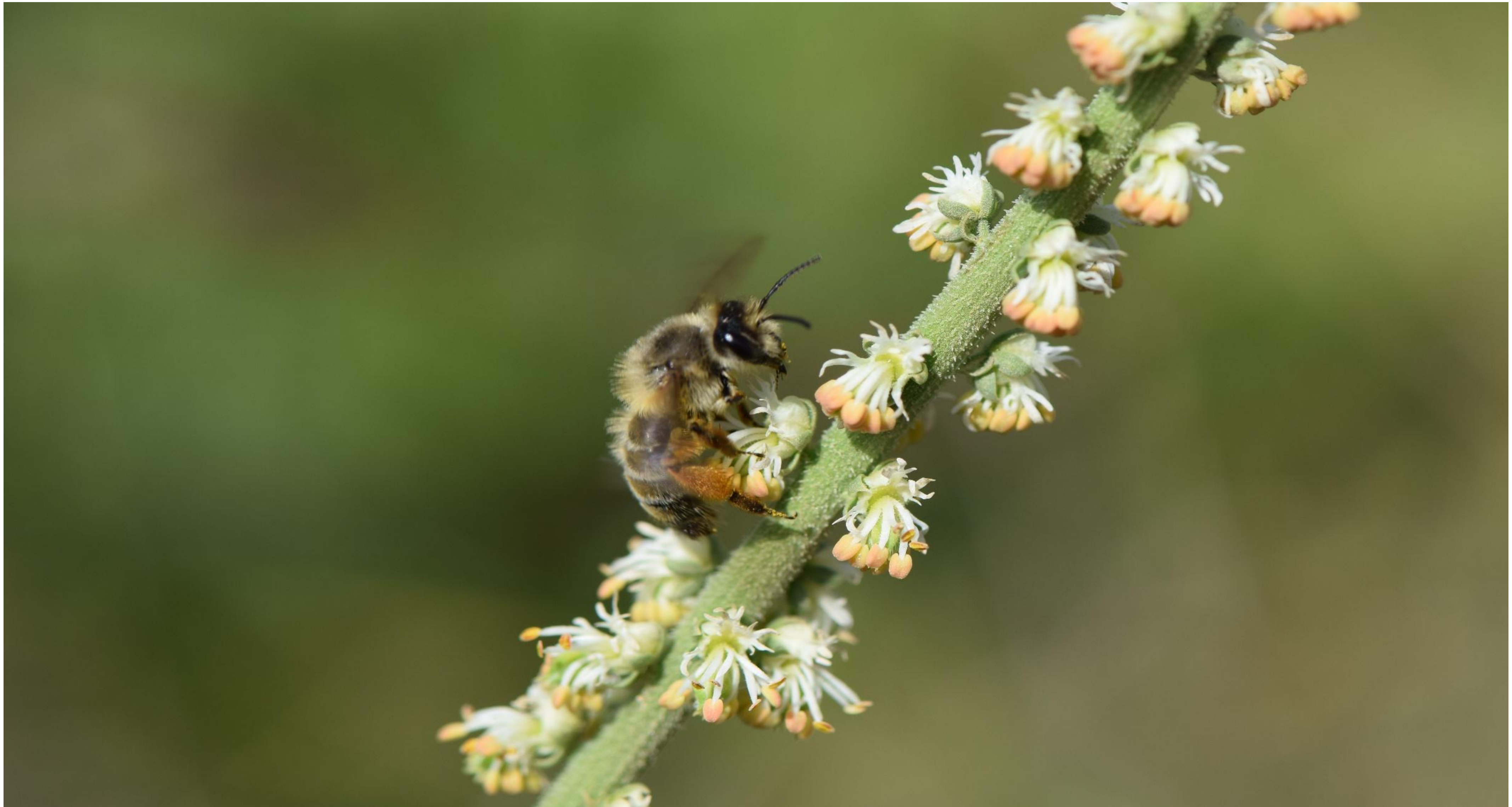

Cenchrus ciliaris

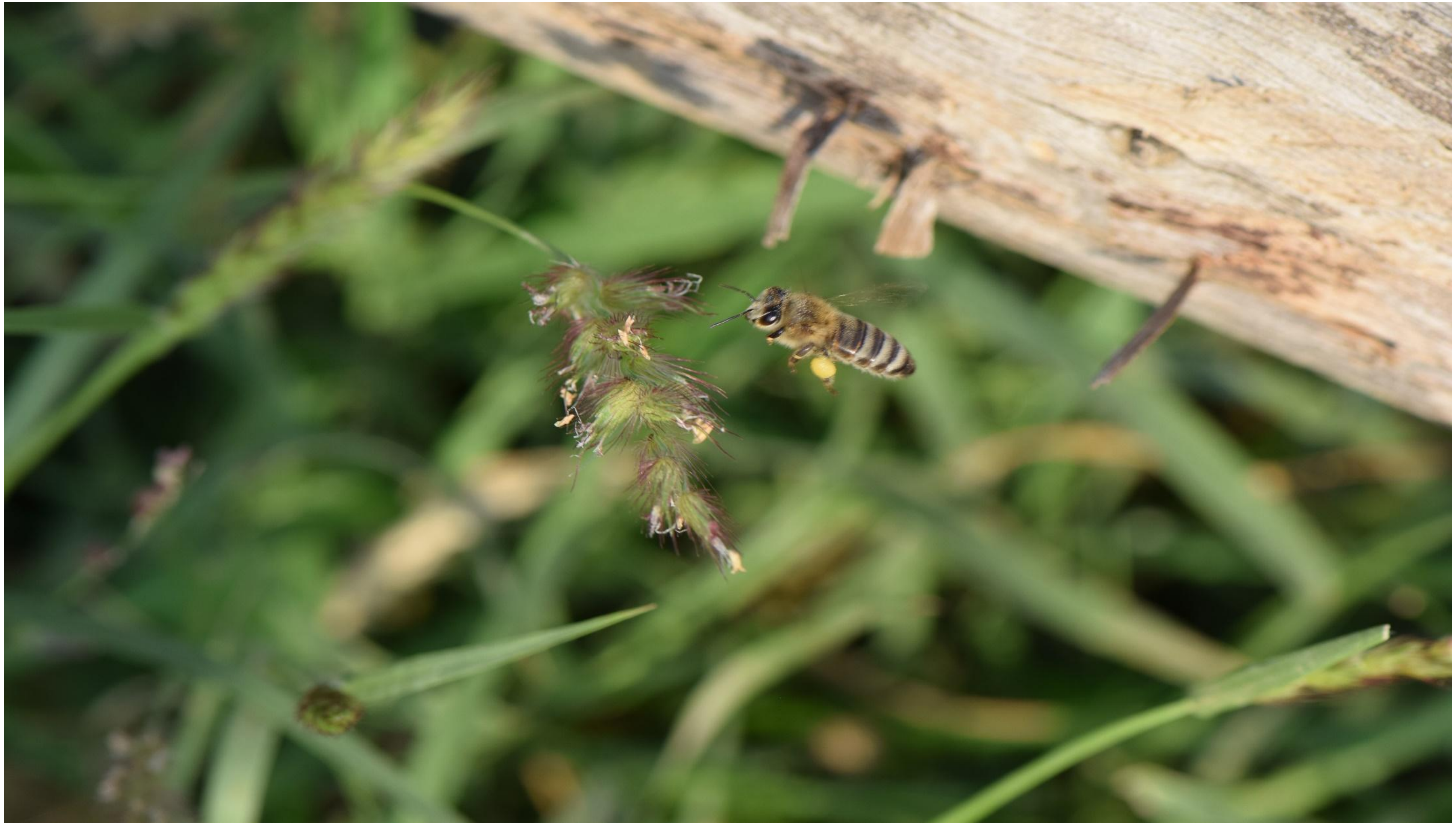

Centuarea sinaica

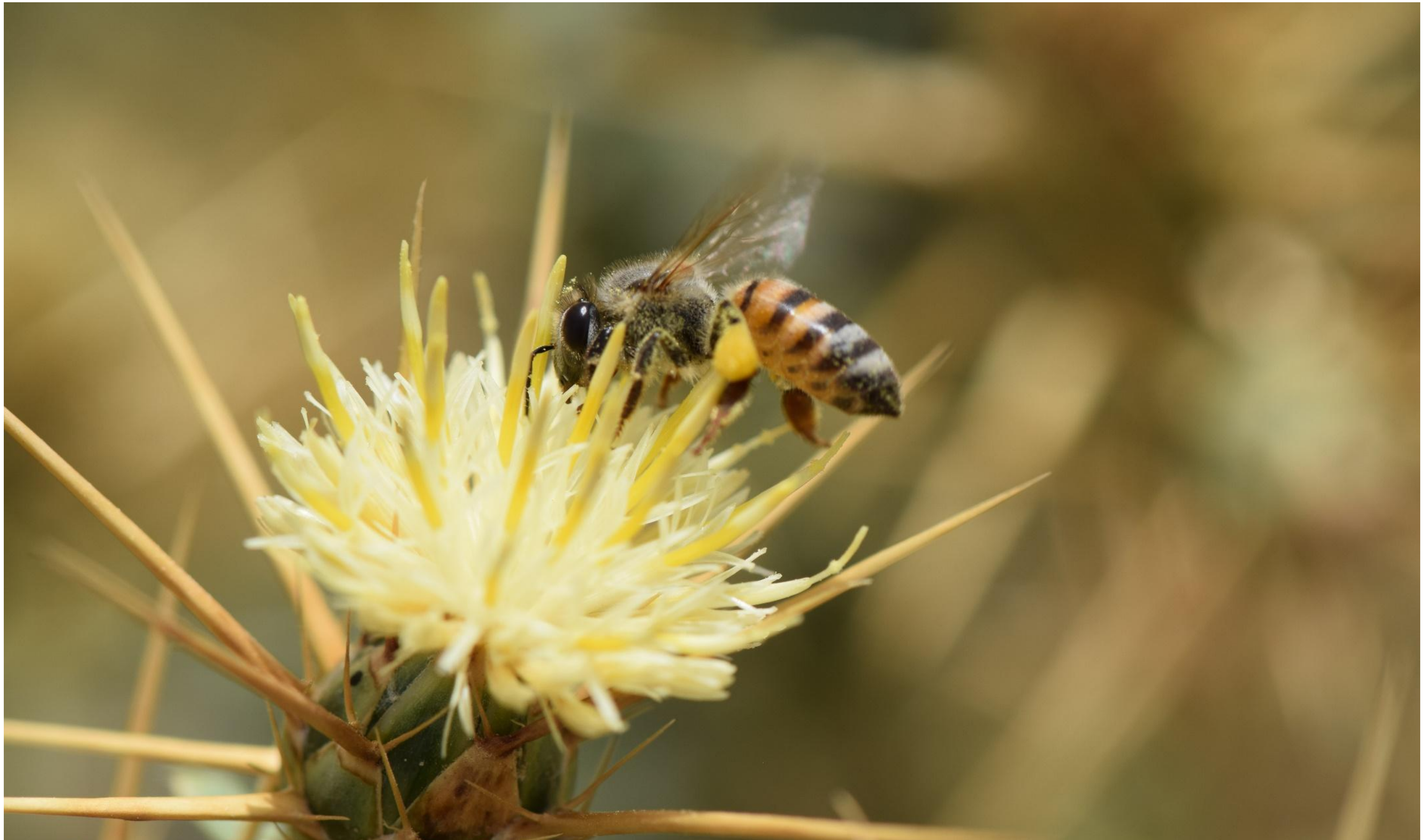

Cichorium intybus

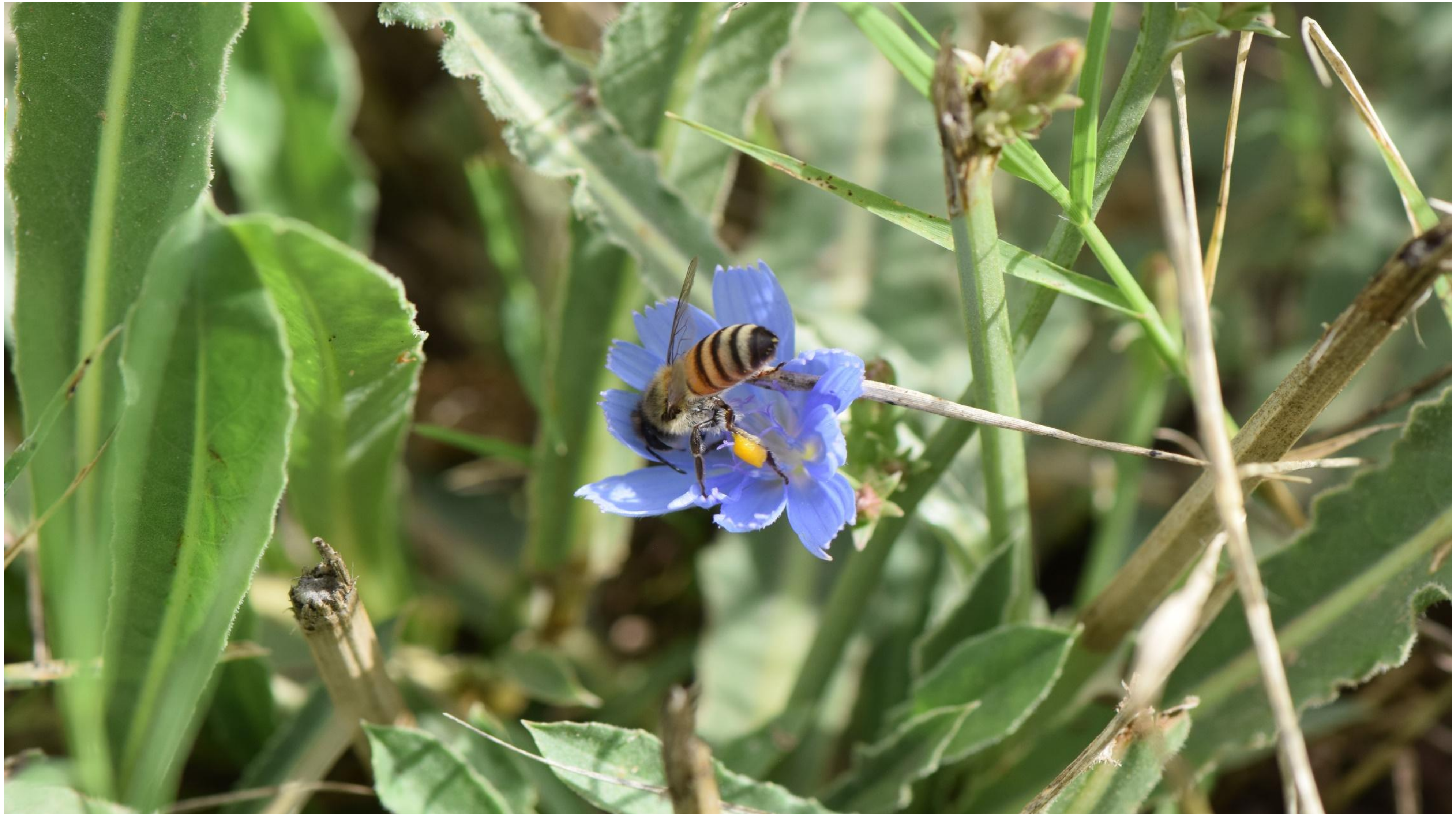

Clematis hirsuta

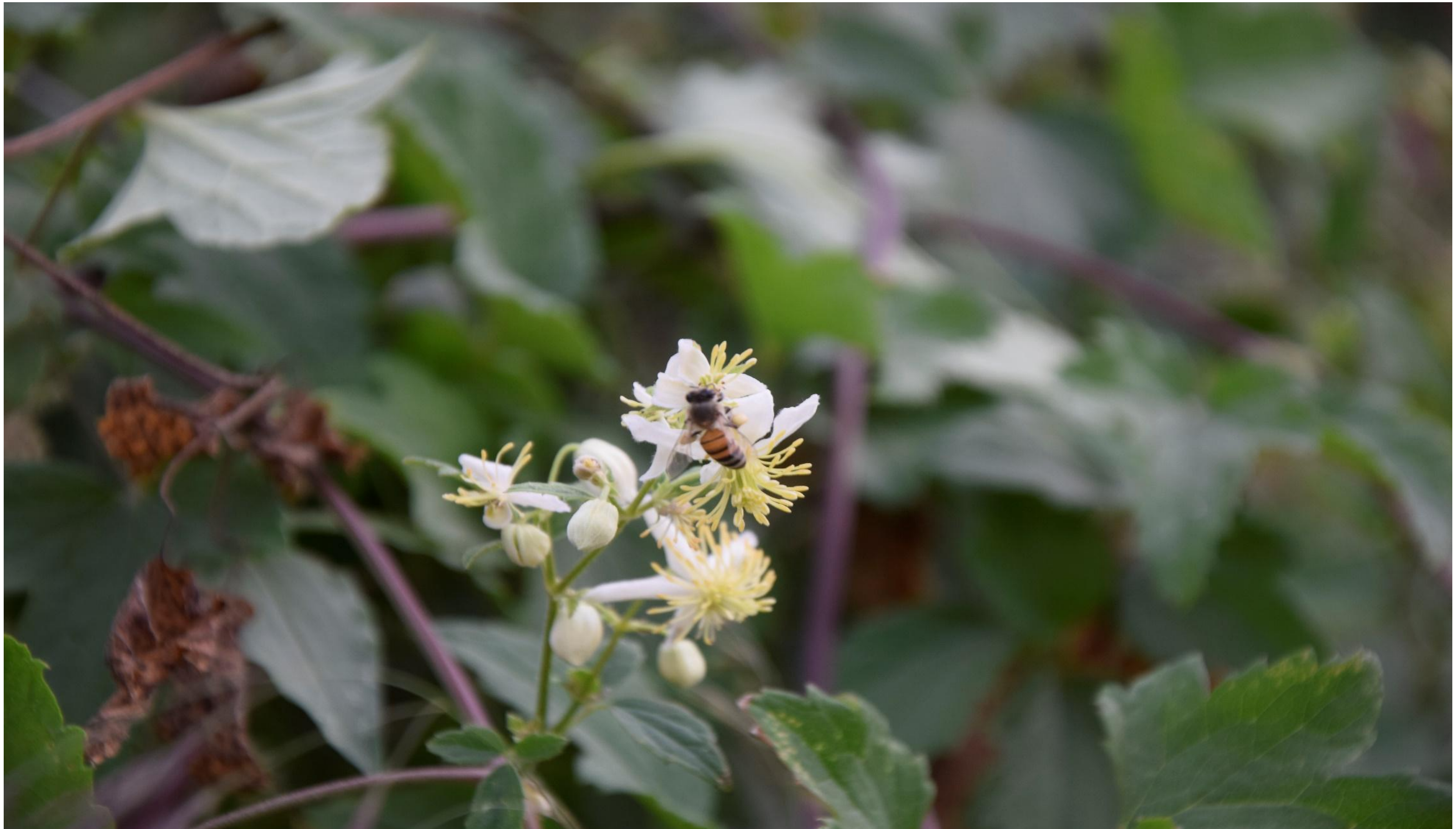

Cleome pallida

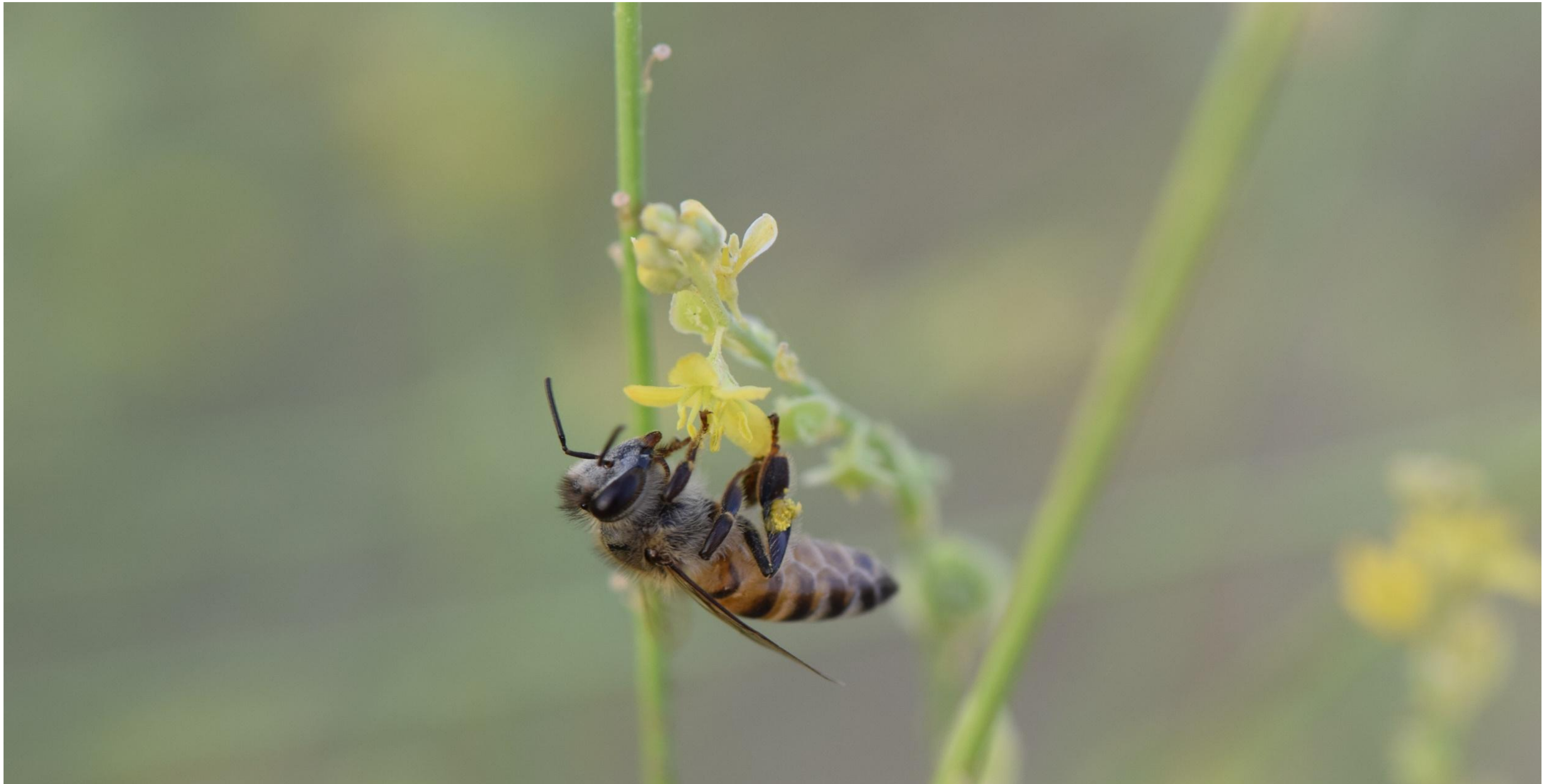

Cleome chrysantha

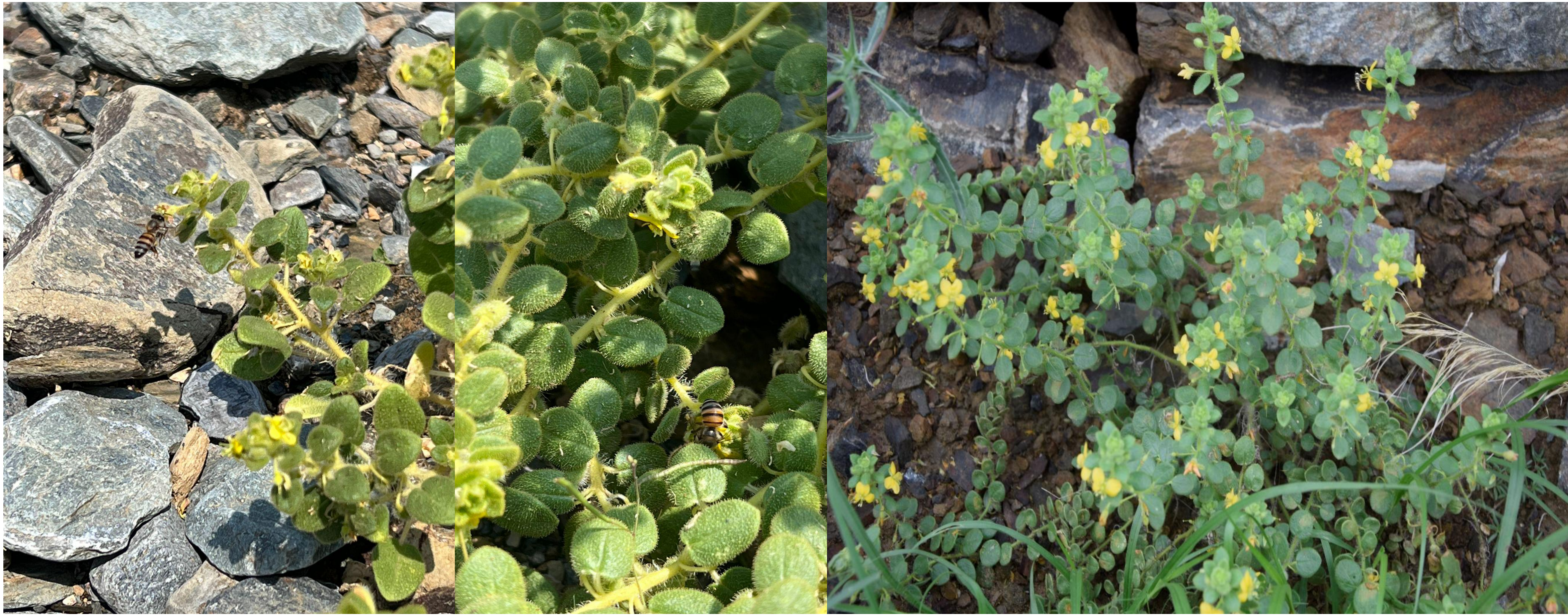

Brassica tournefortii

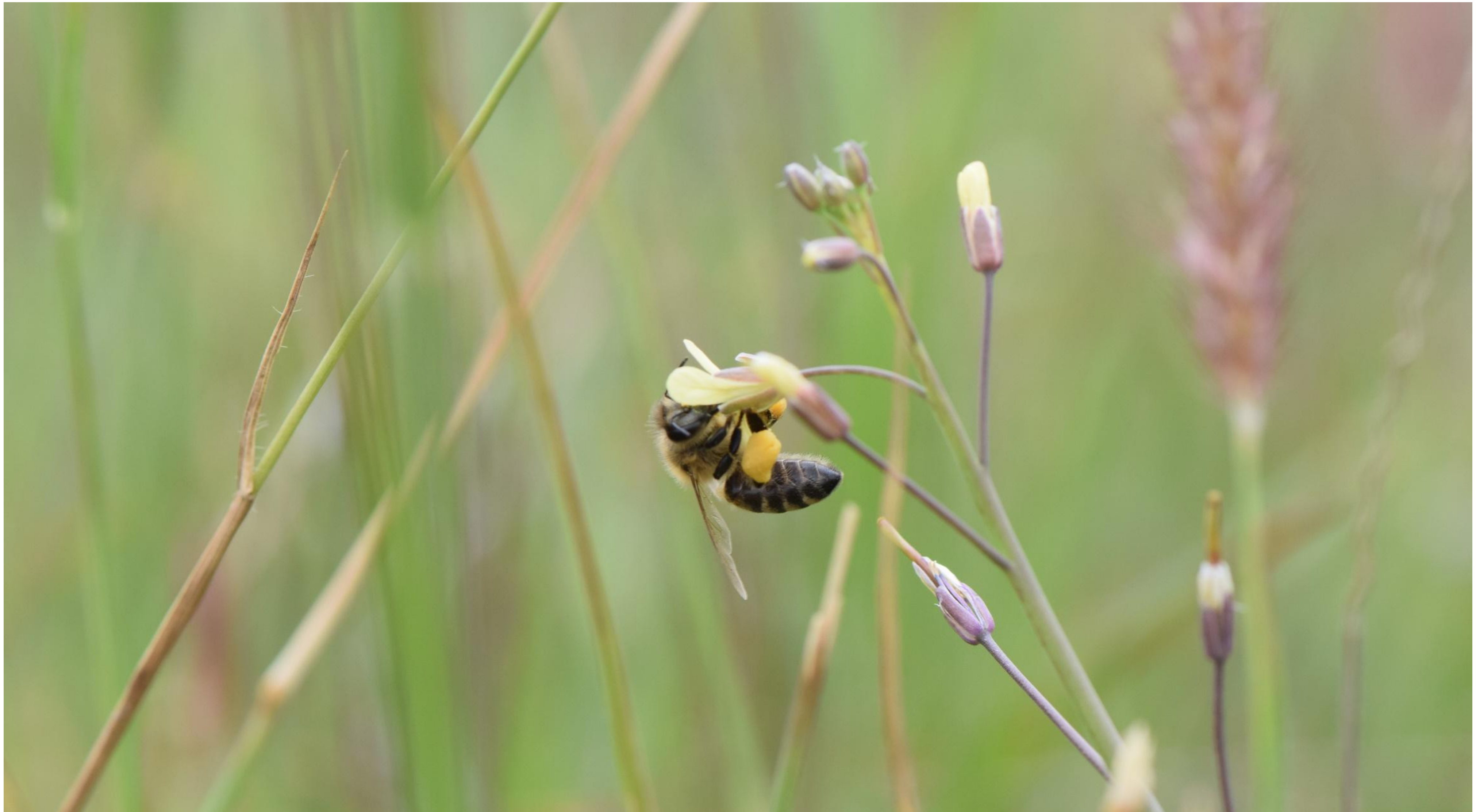

Convolvulus arvensis

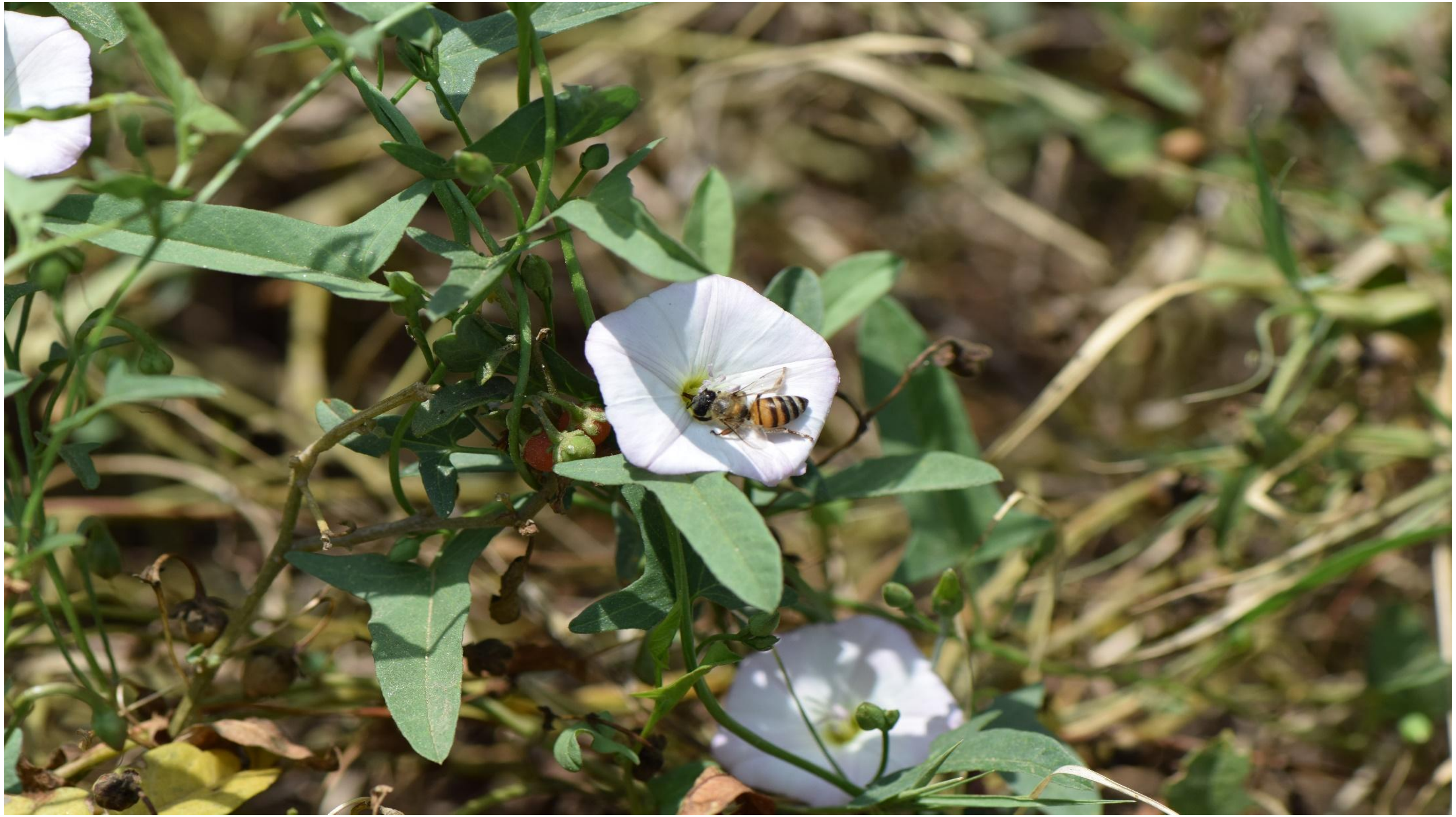

Cynanchum viminale

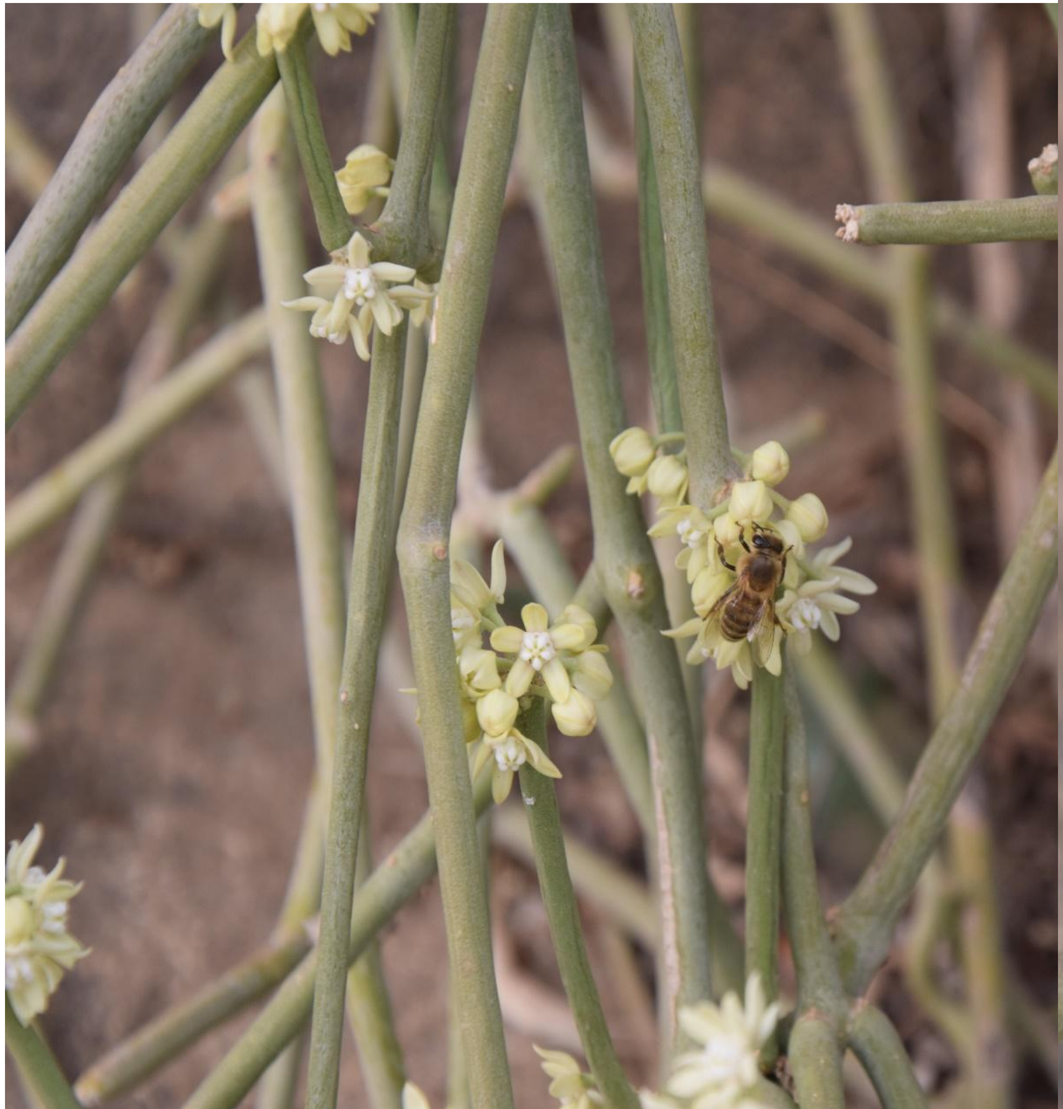
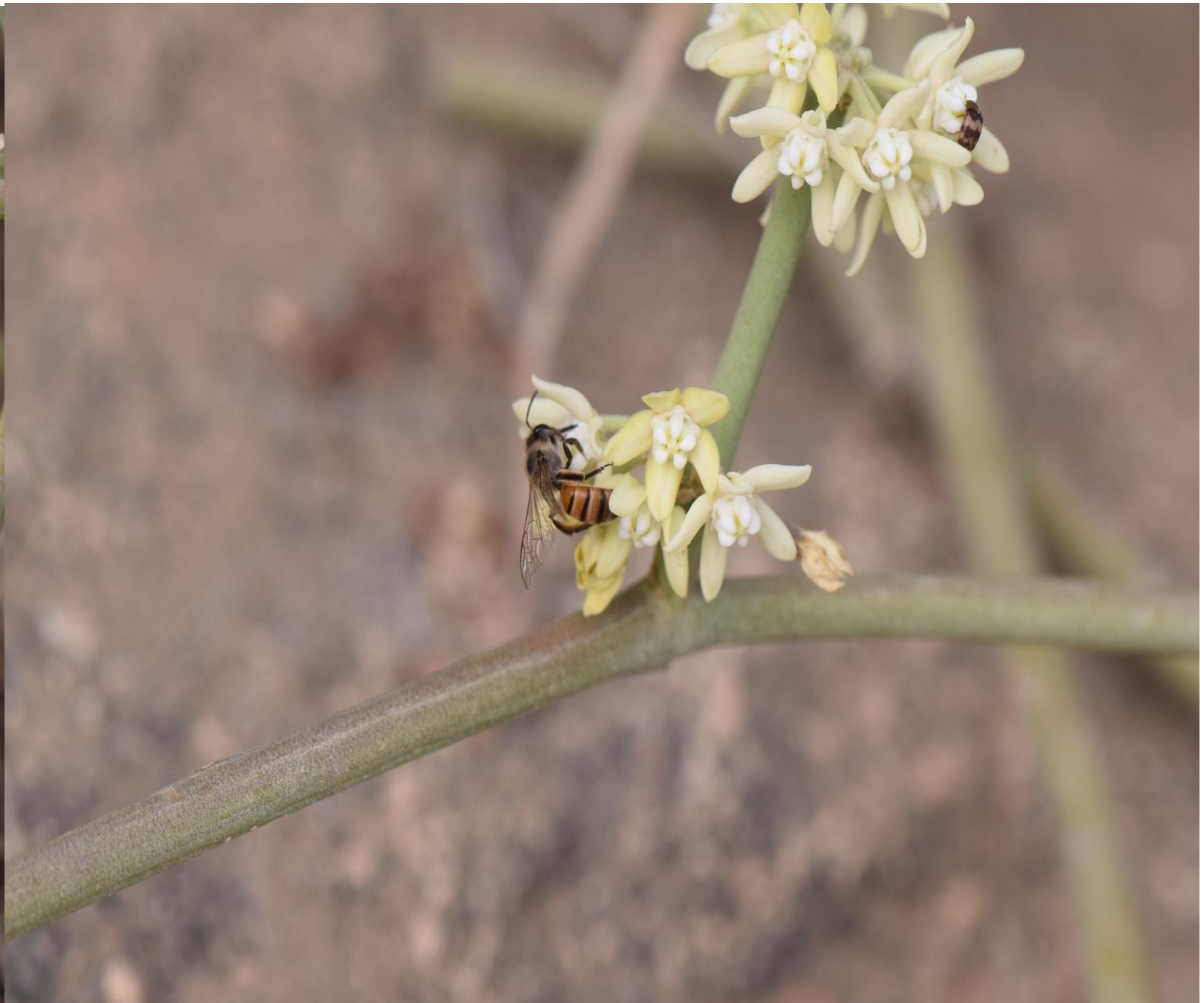

Cynodon dactylon

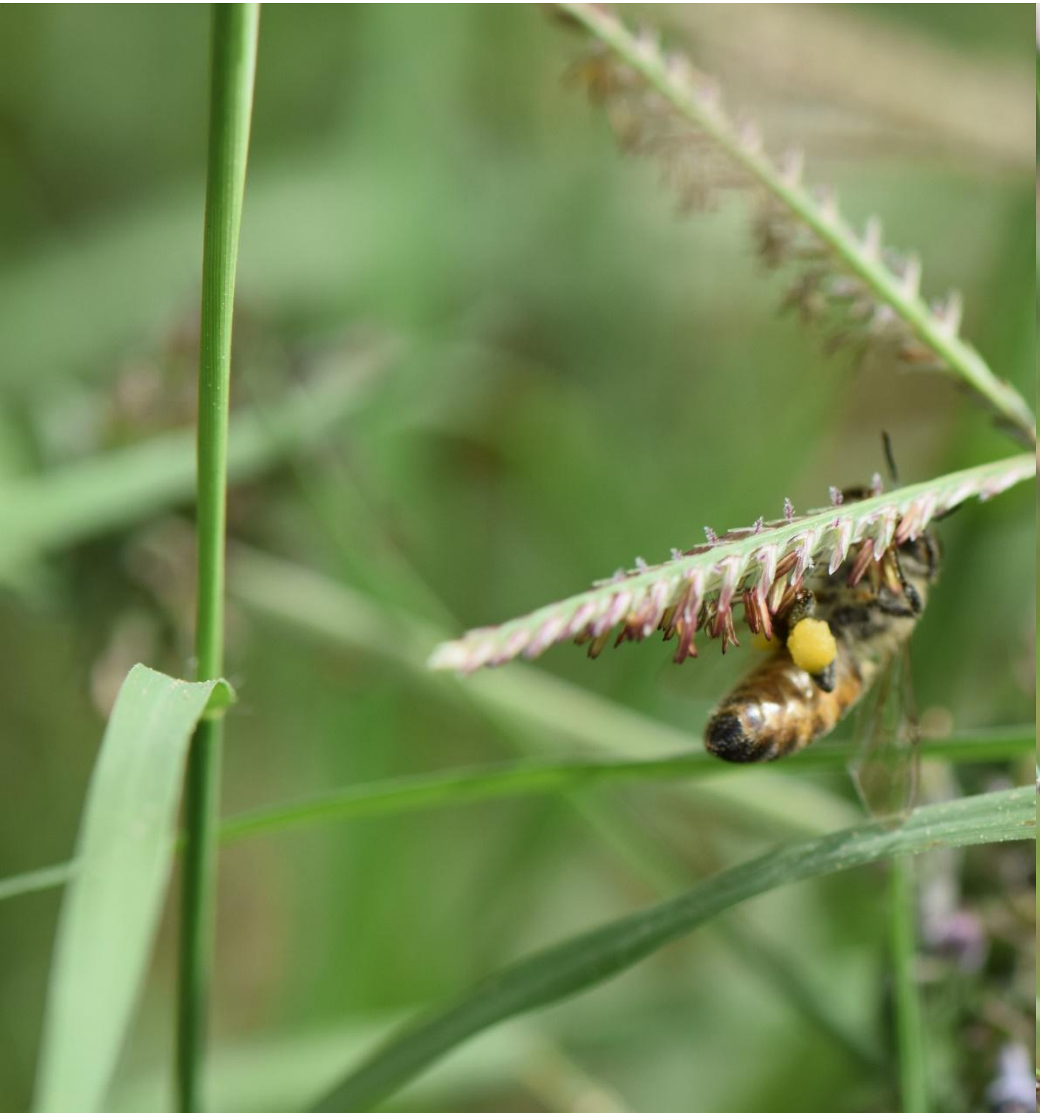
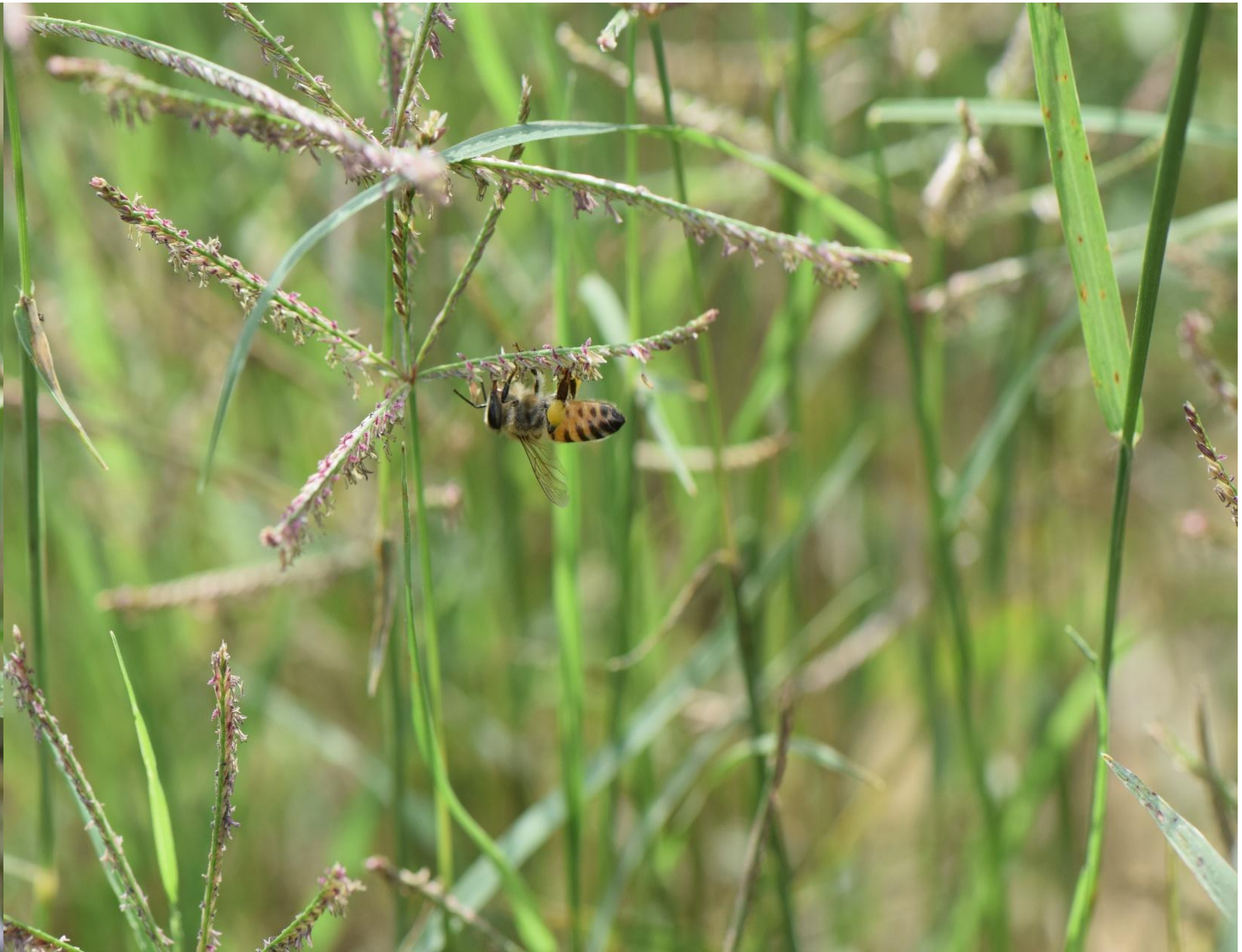

Delonix elata

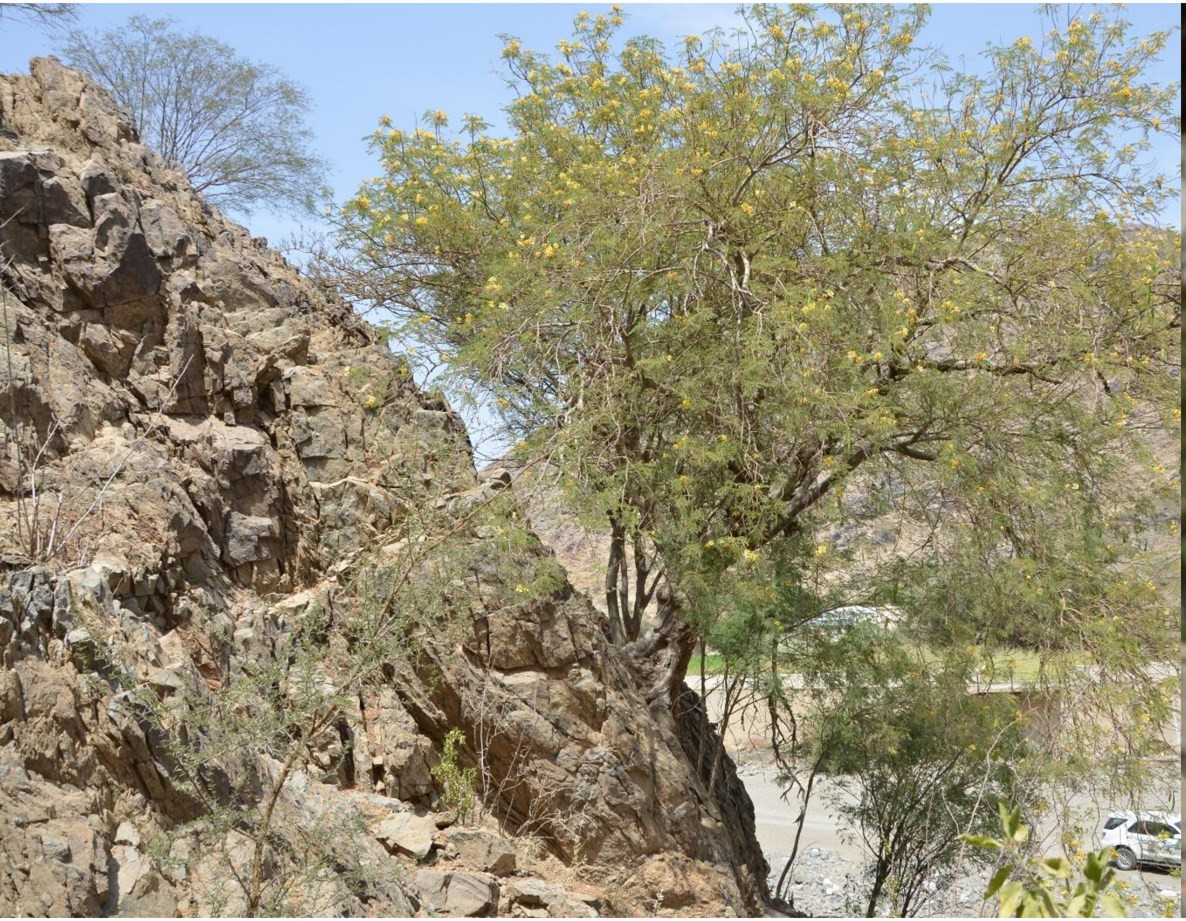

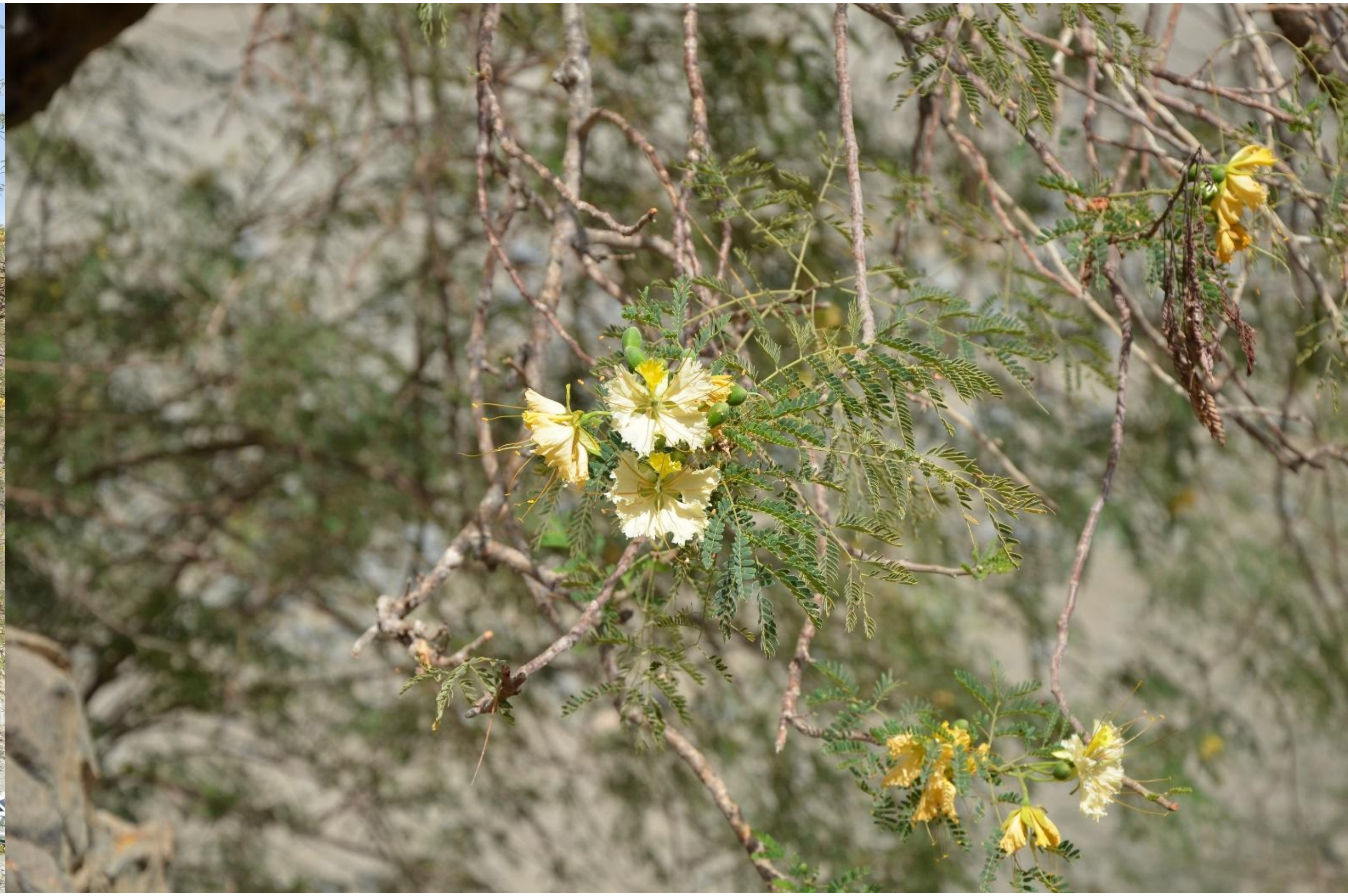

Dodonaea viscosa

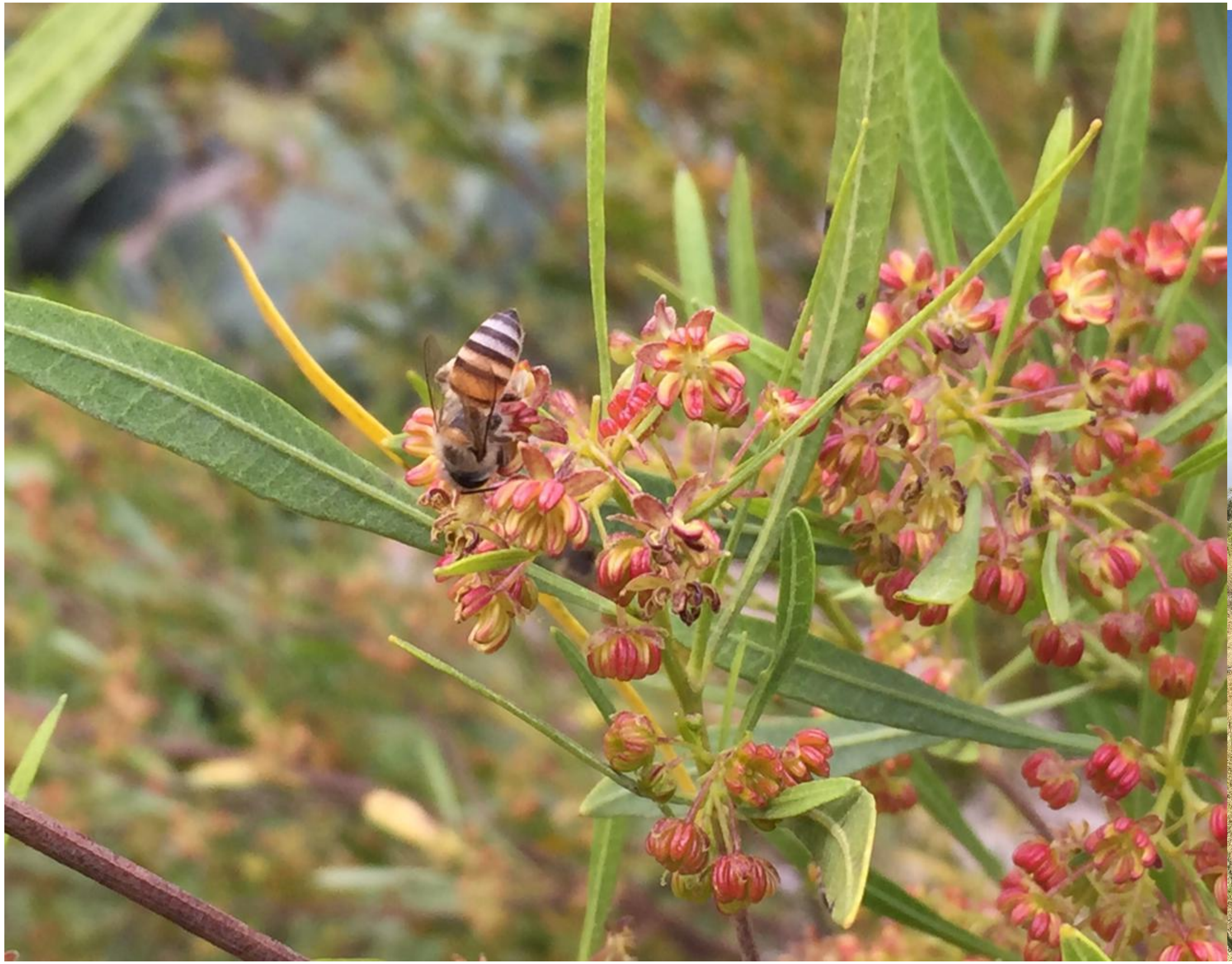

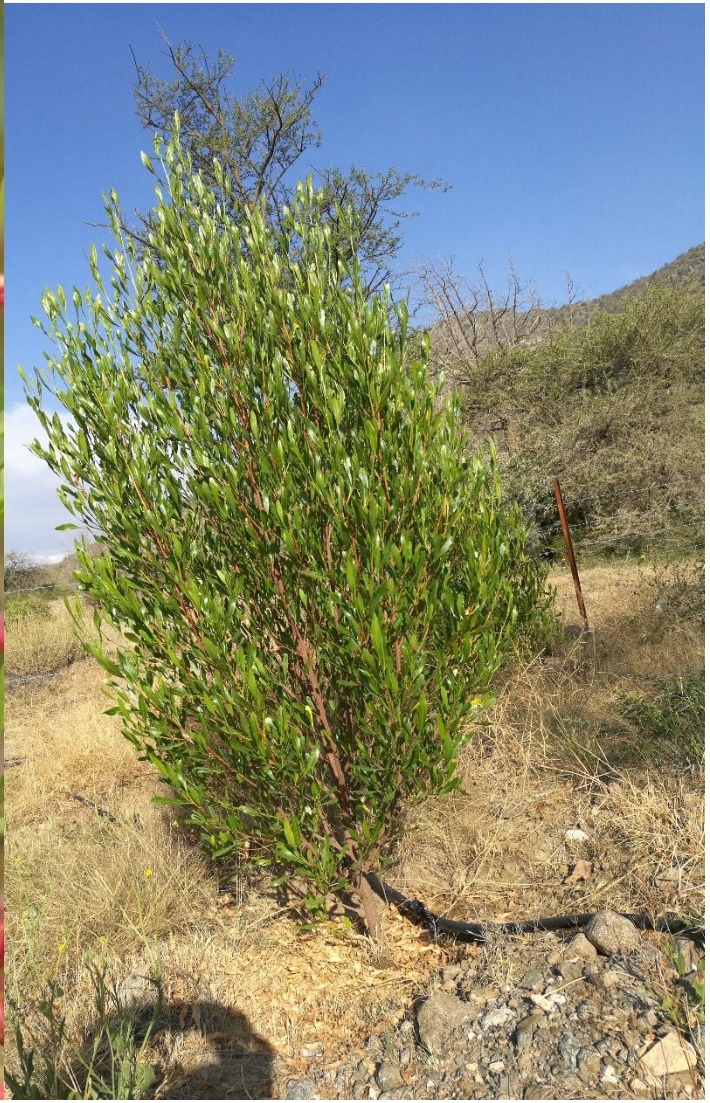

Echinops sp

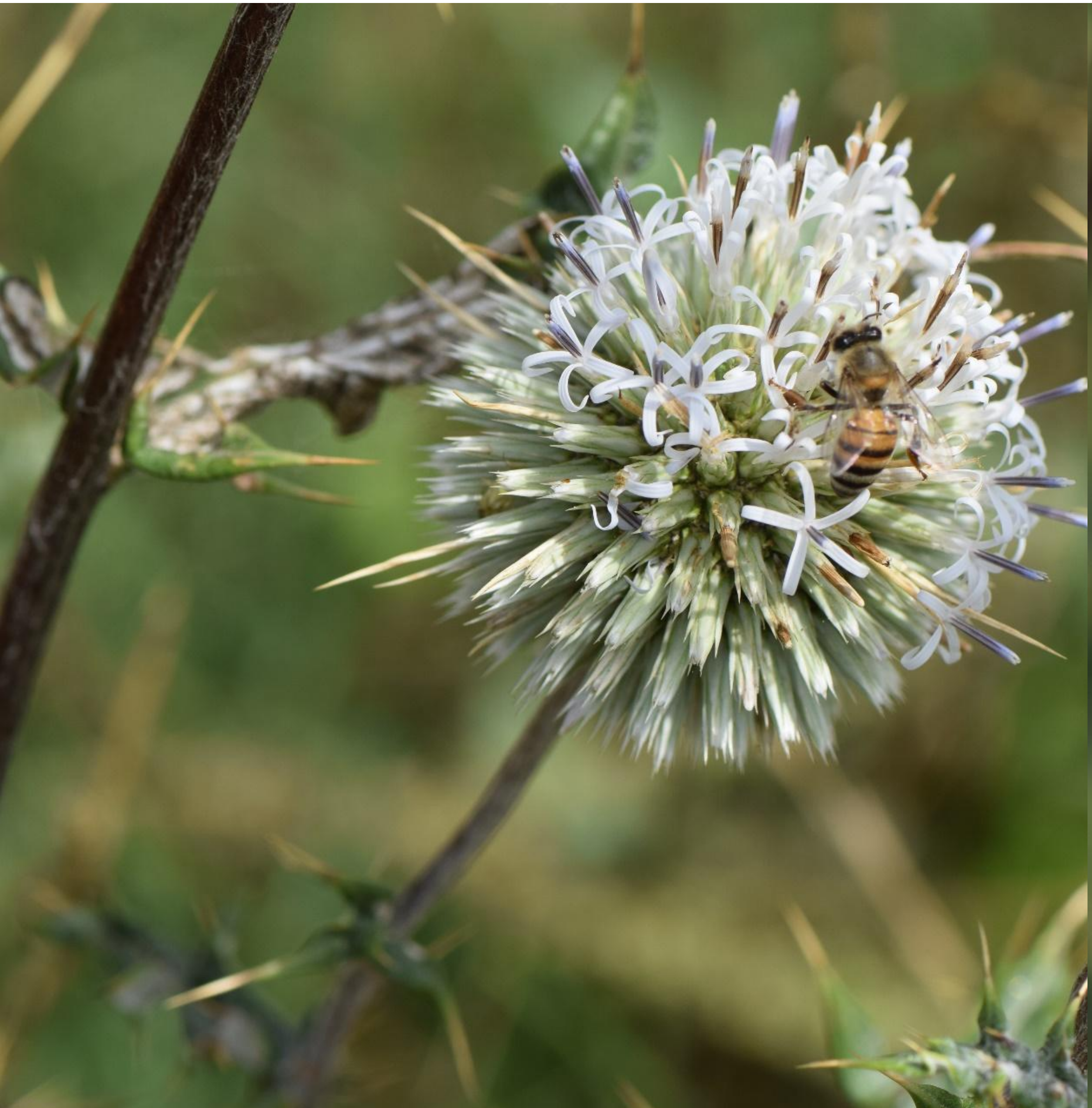

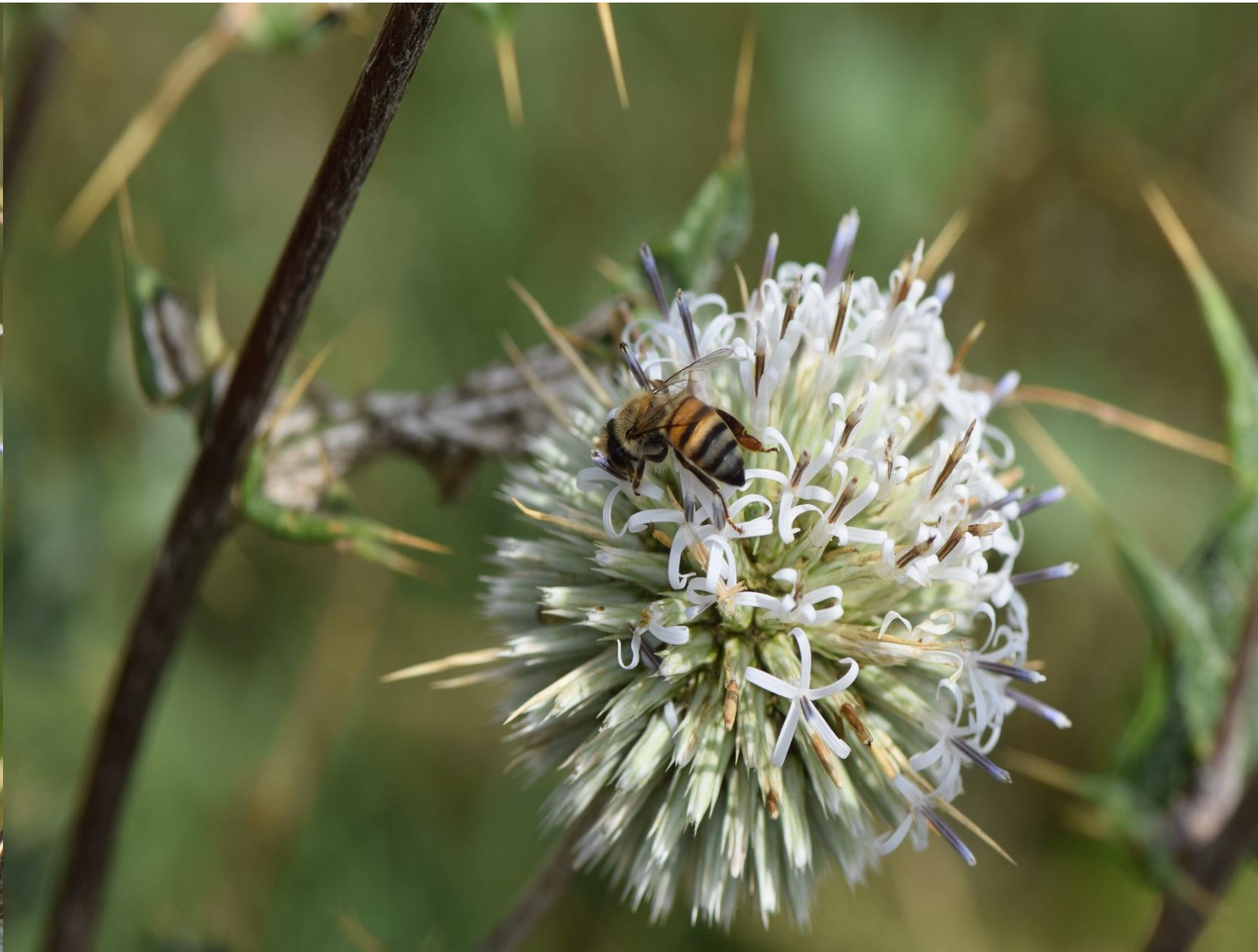

Echium rauwolfii

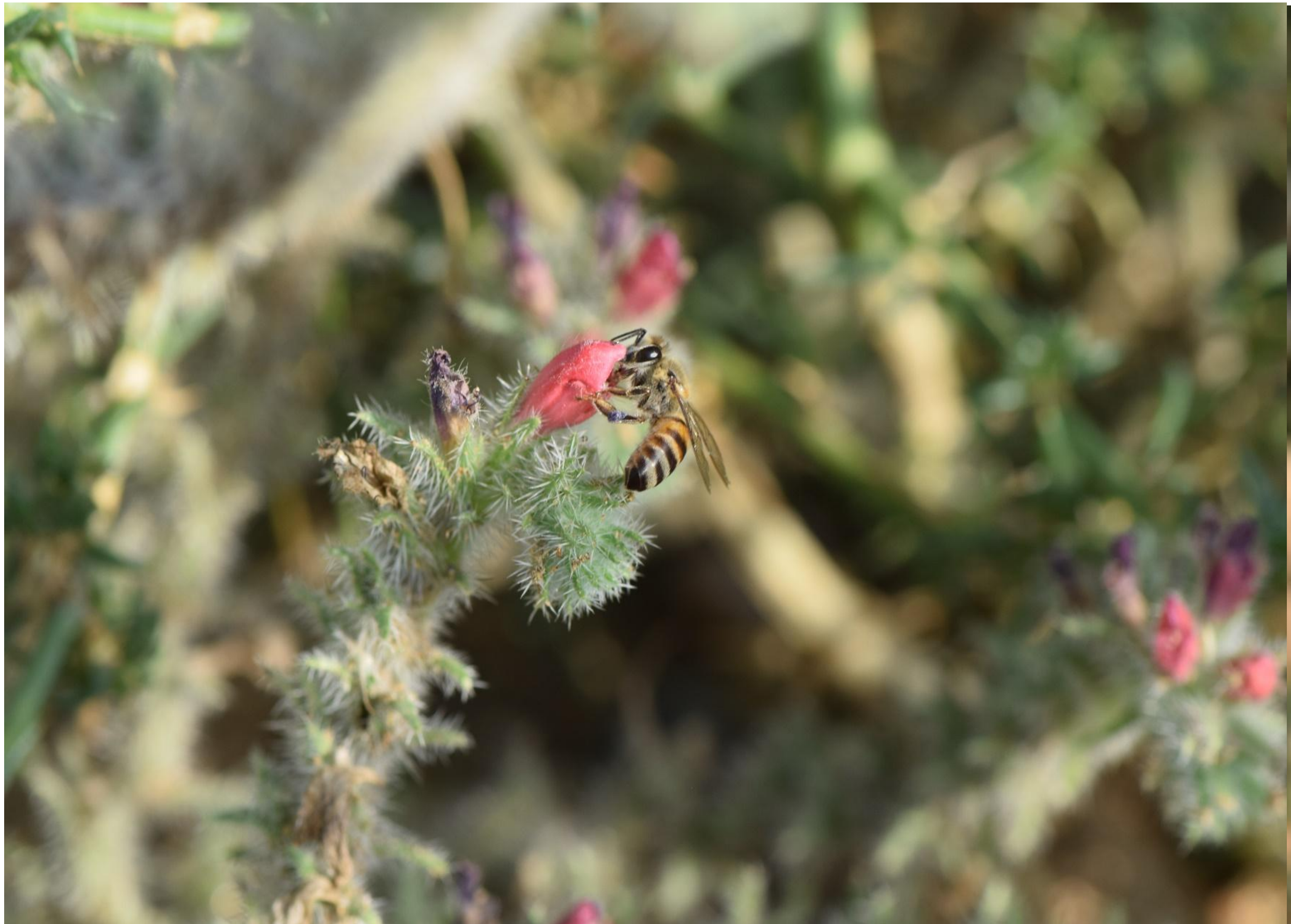

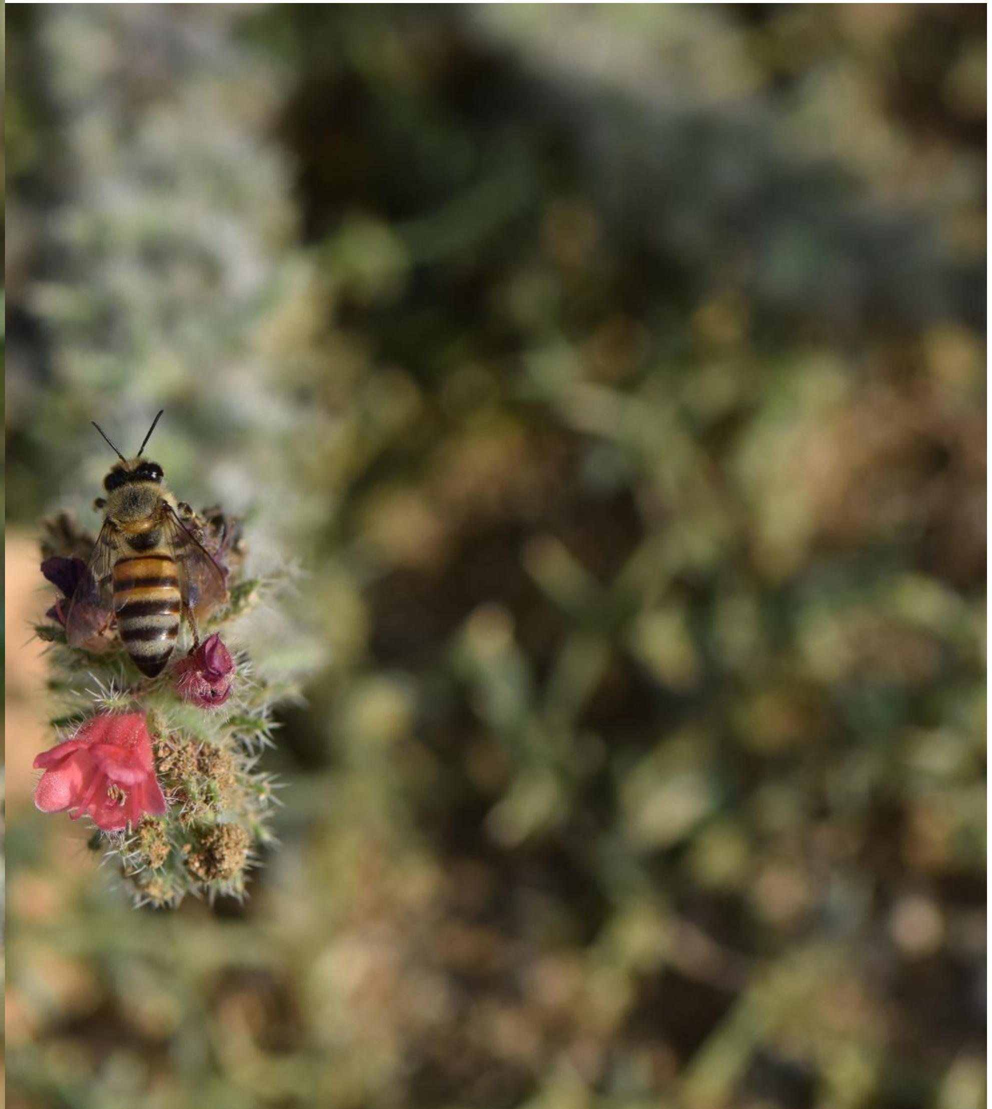

Eruca sativa

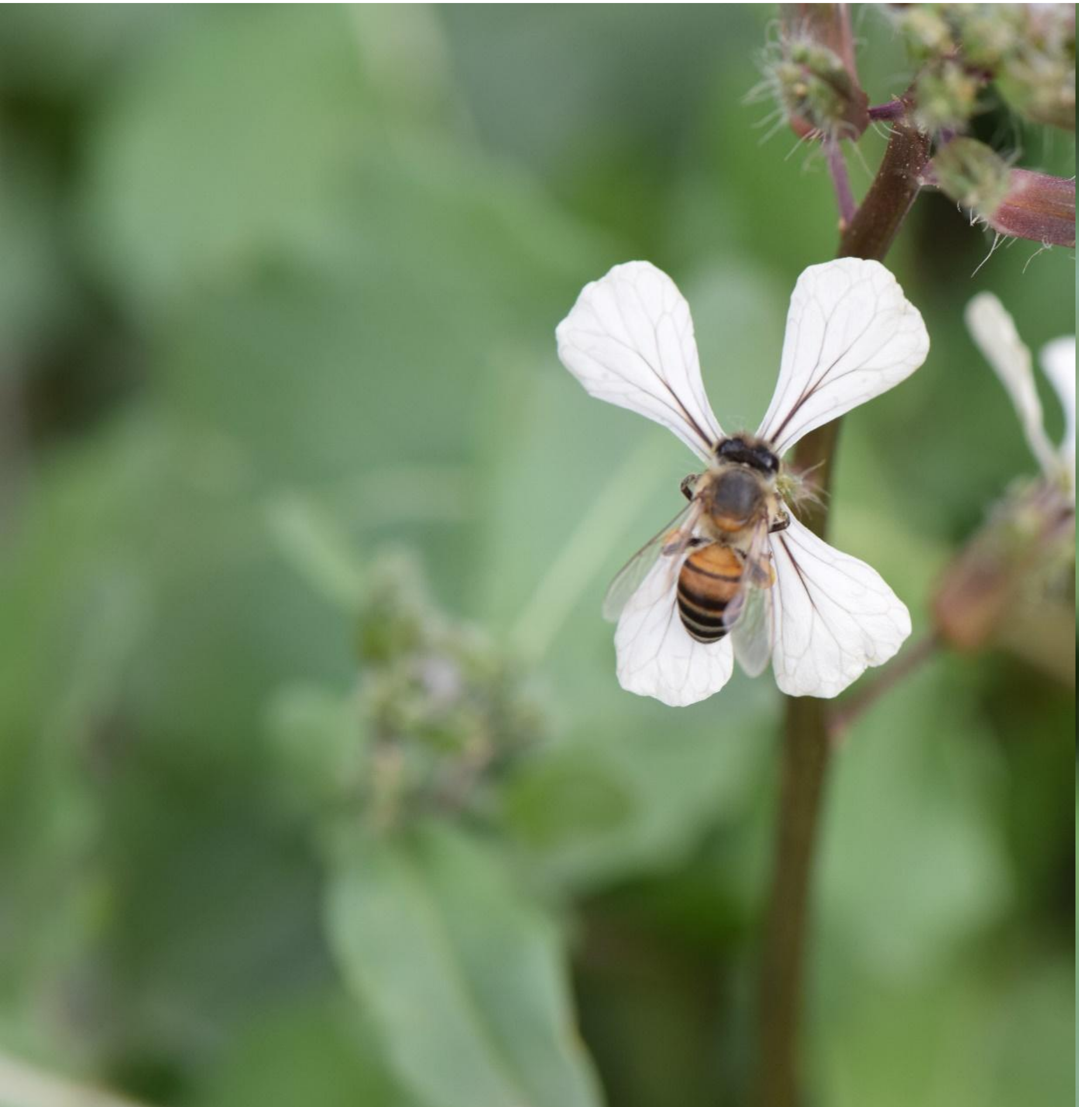

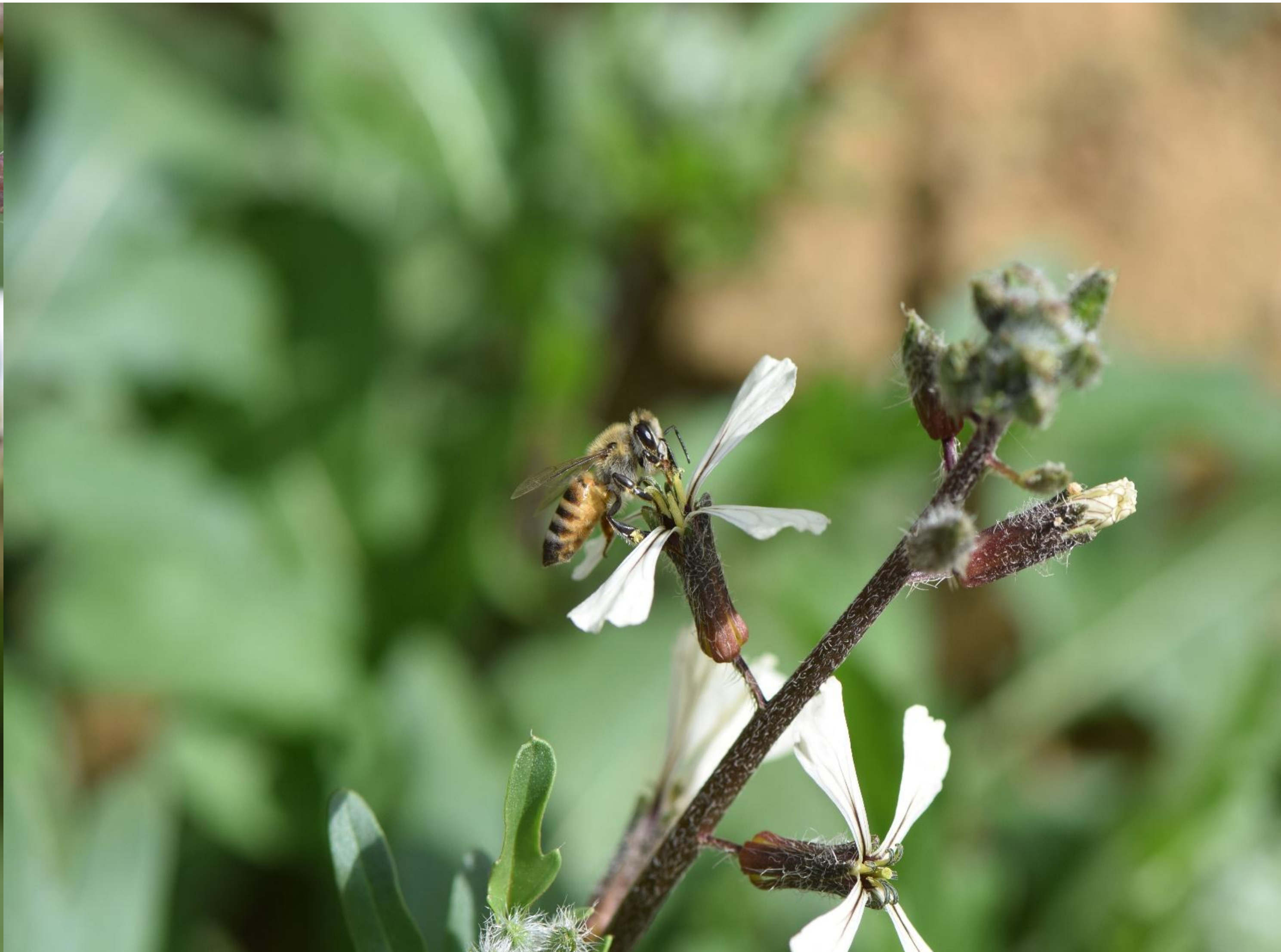

Euphorbia cuneata

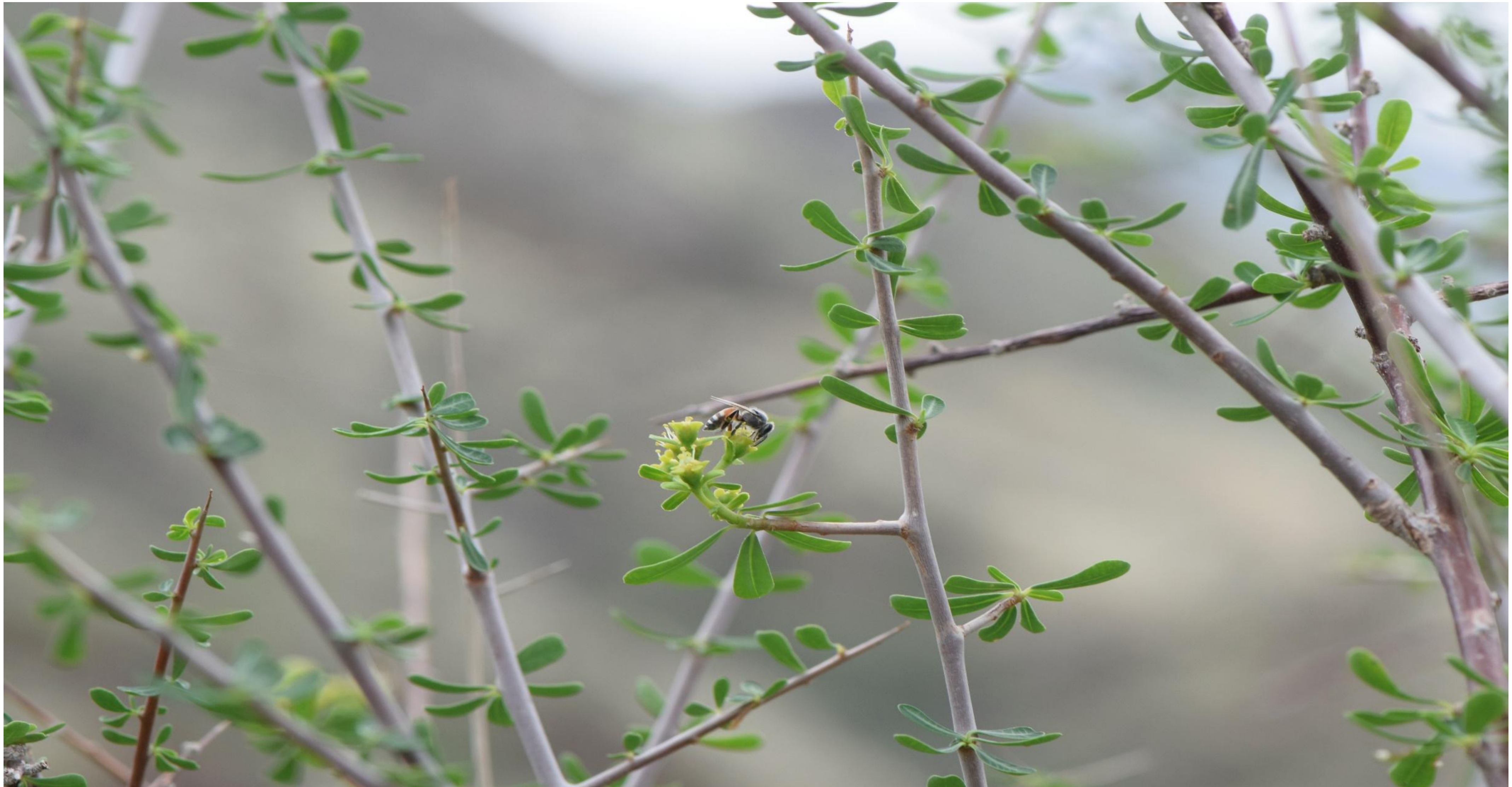

Euphorbia inarticulate

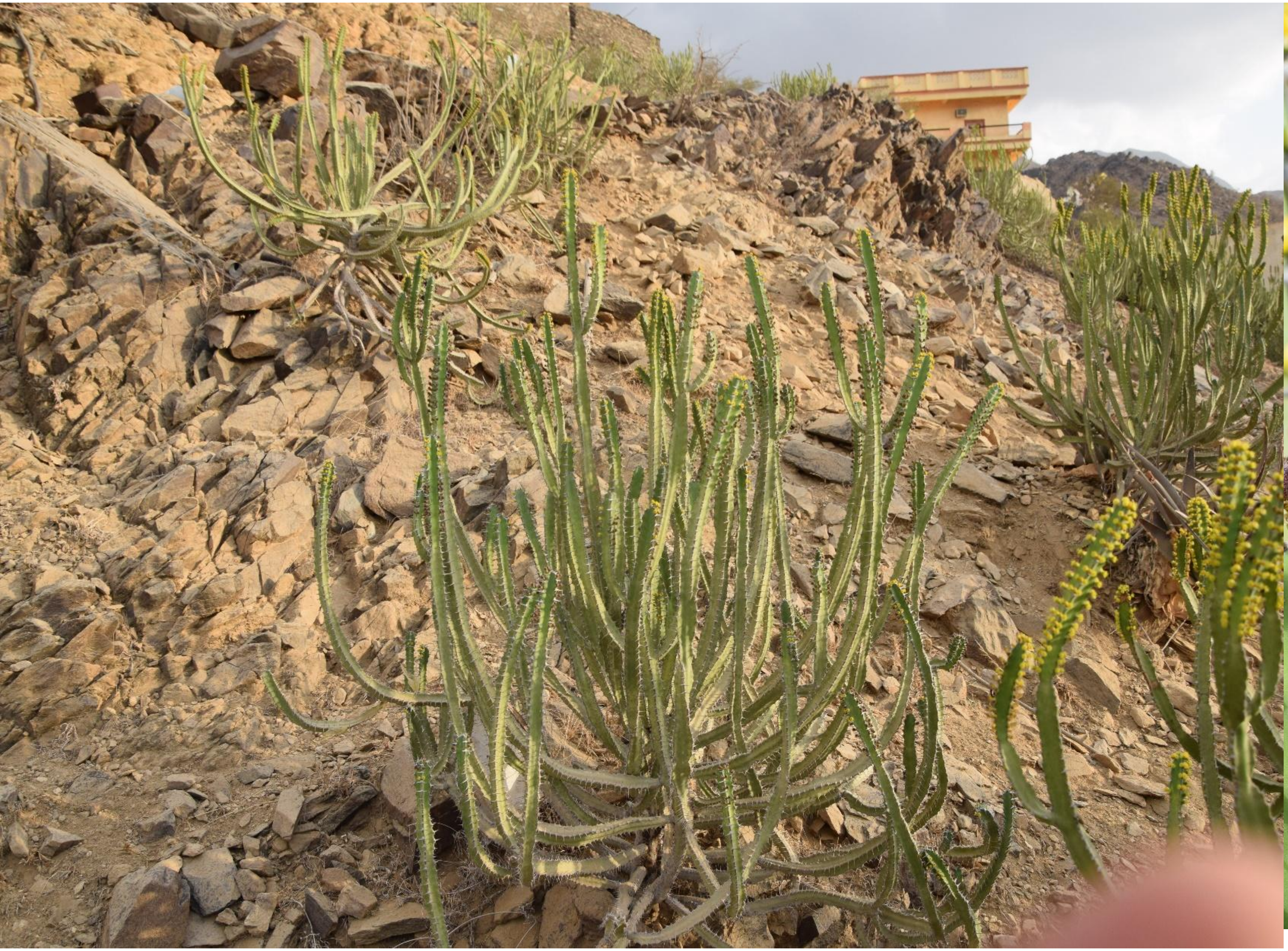

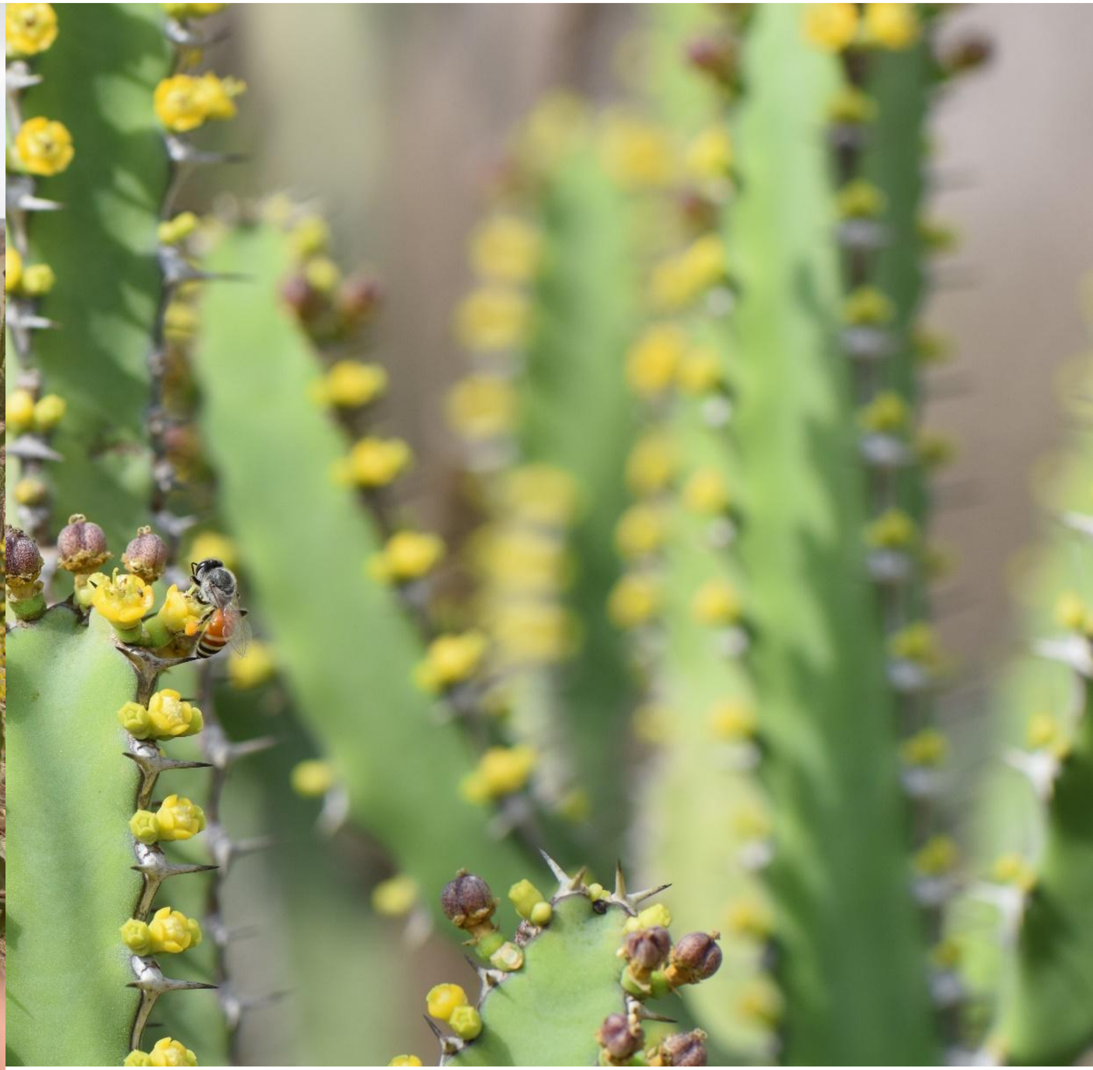

Foeniculum vulgare

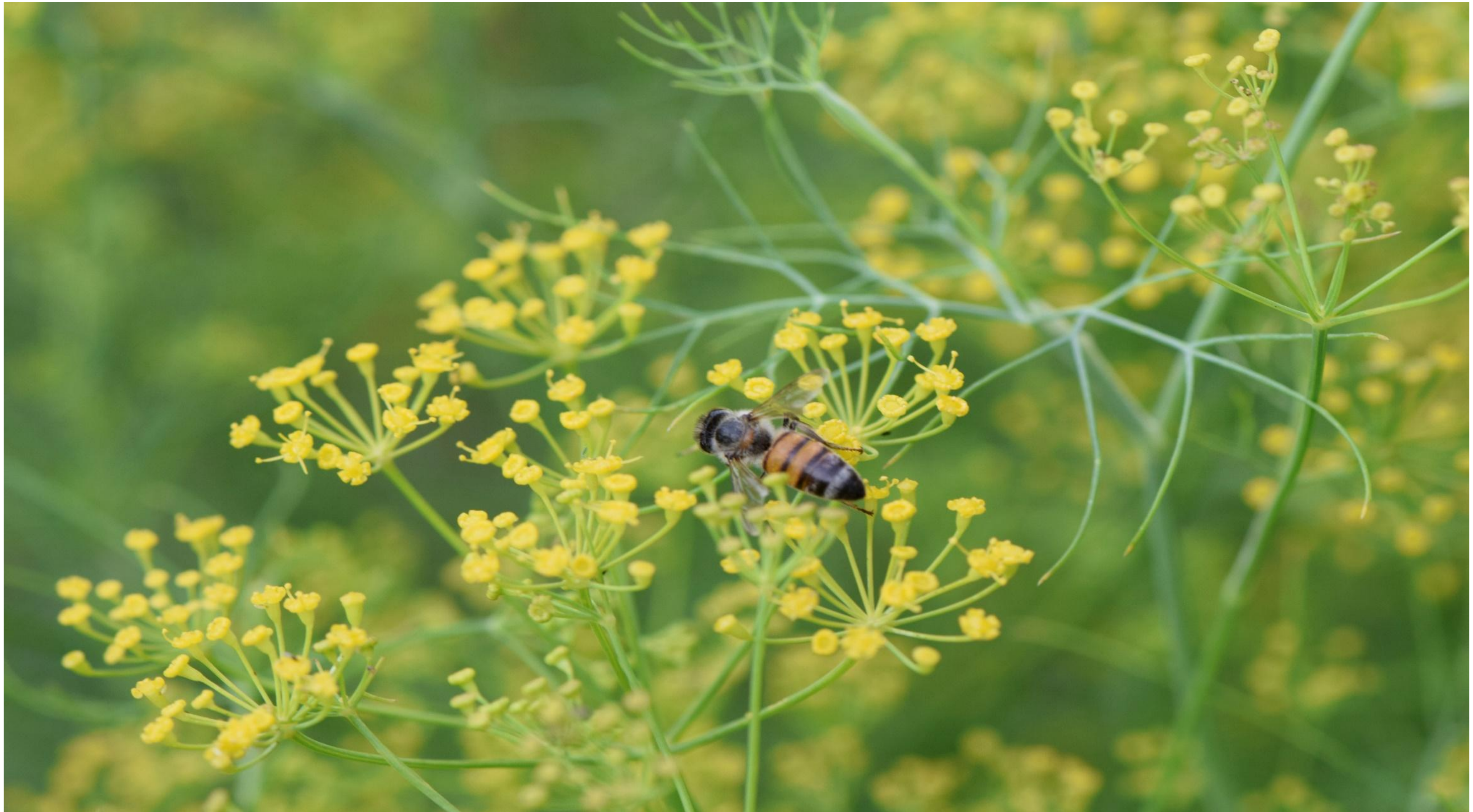

Glinus lotoides var. virens

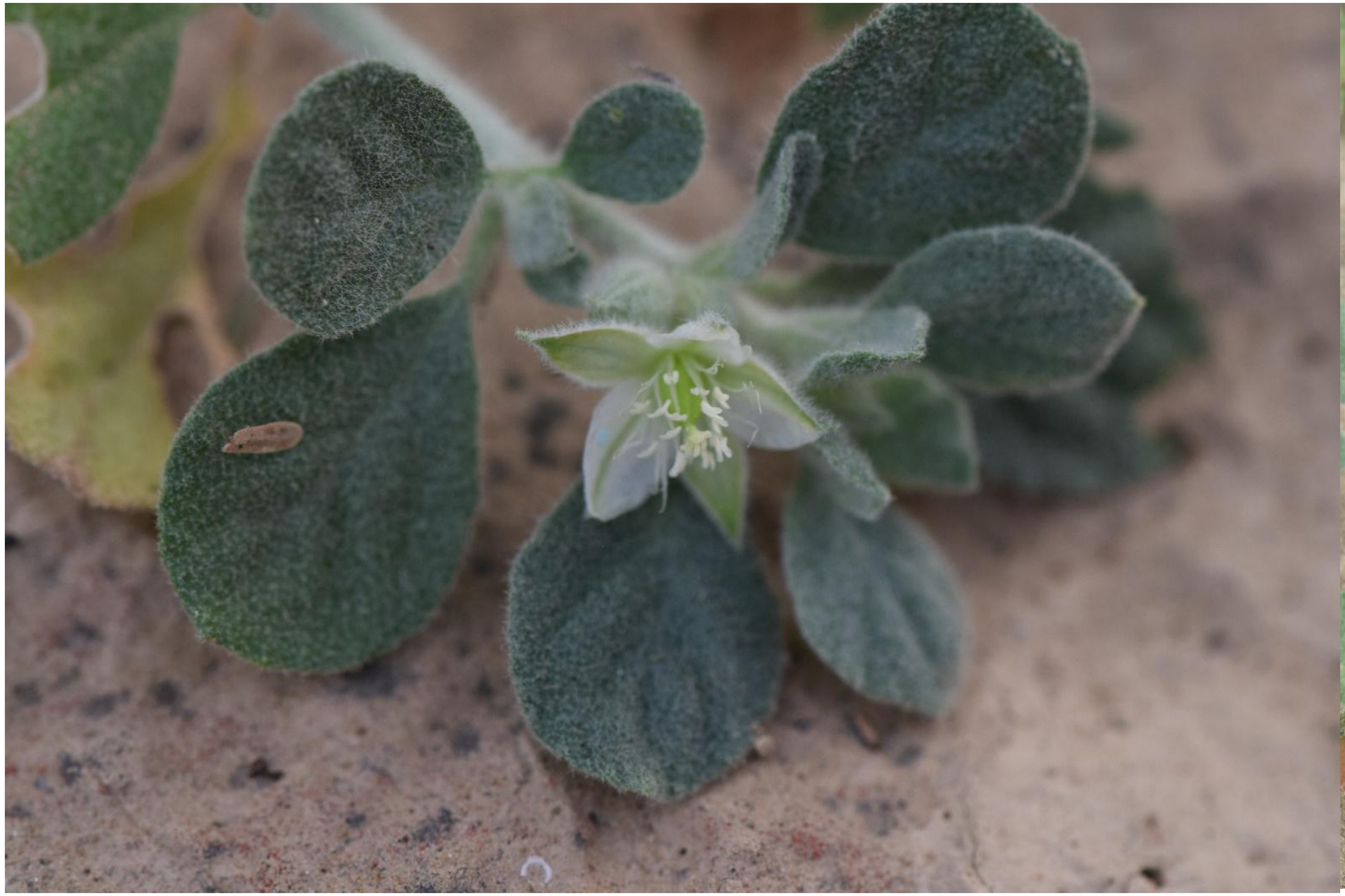

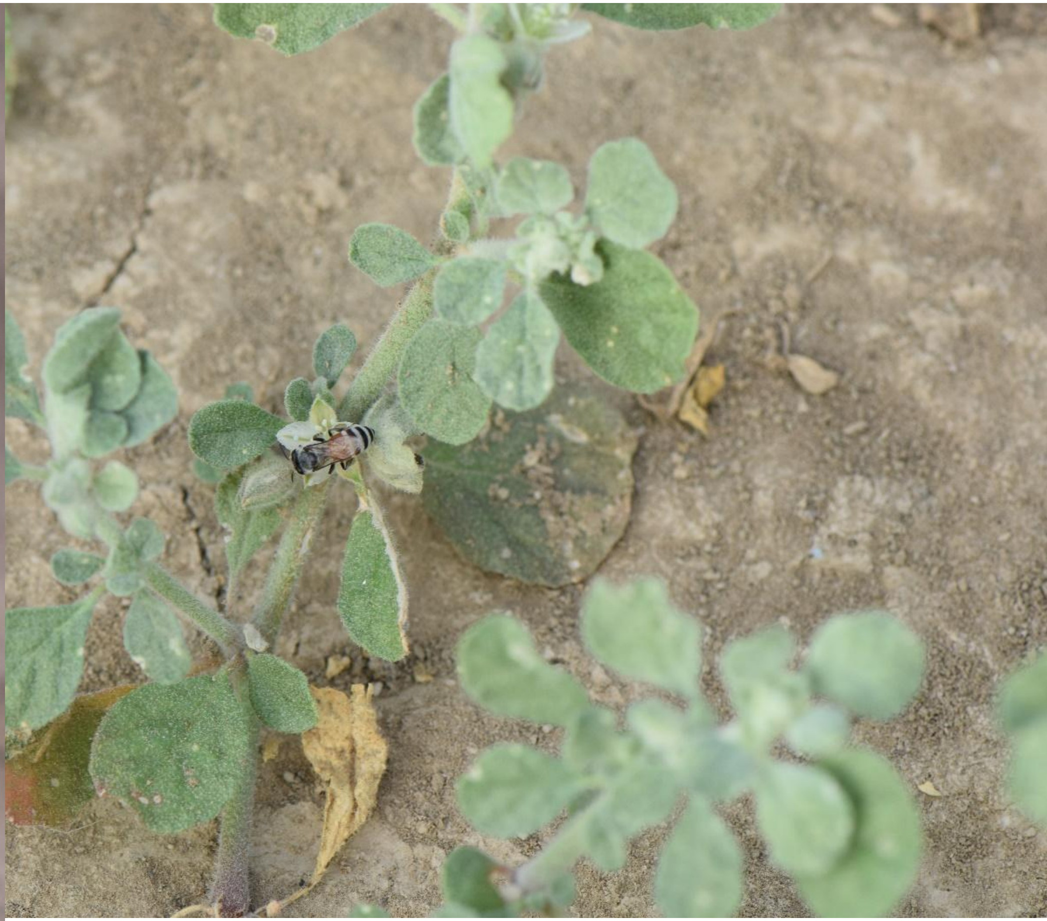

Grewia tenax

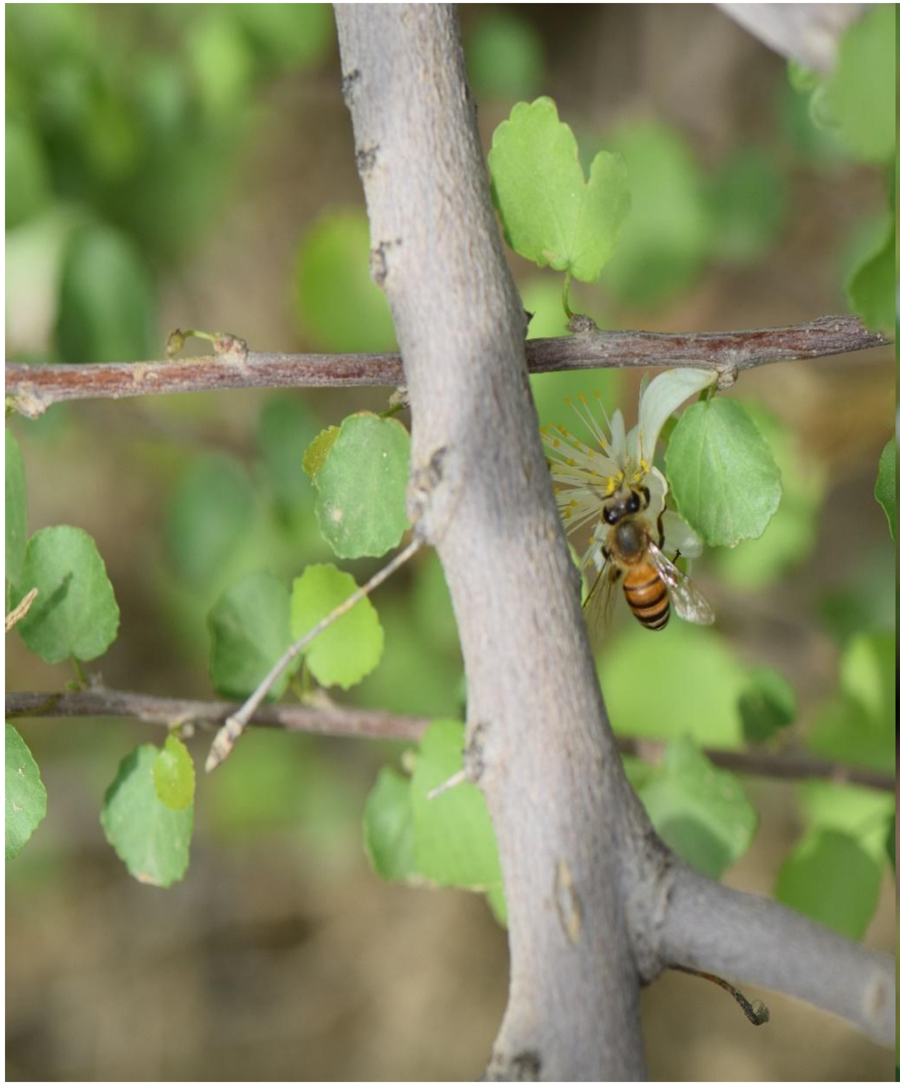

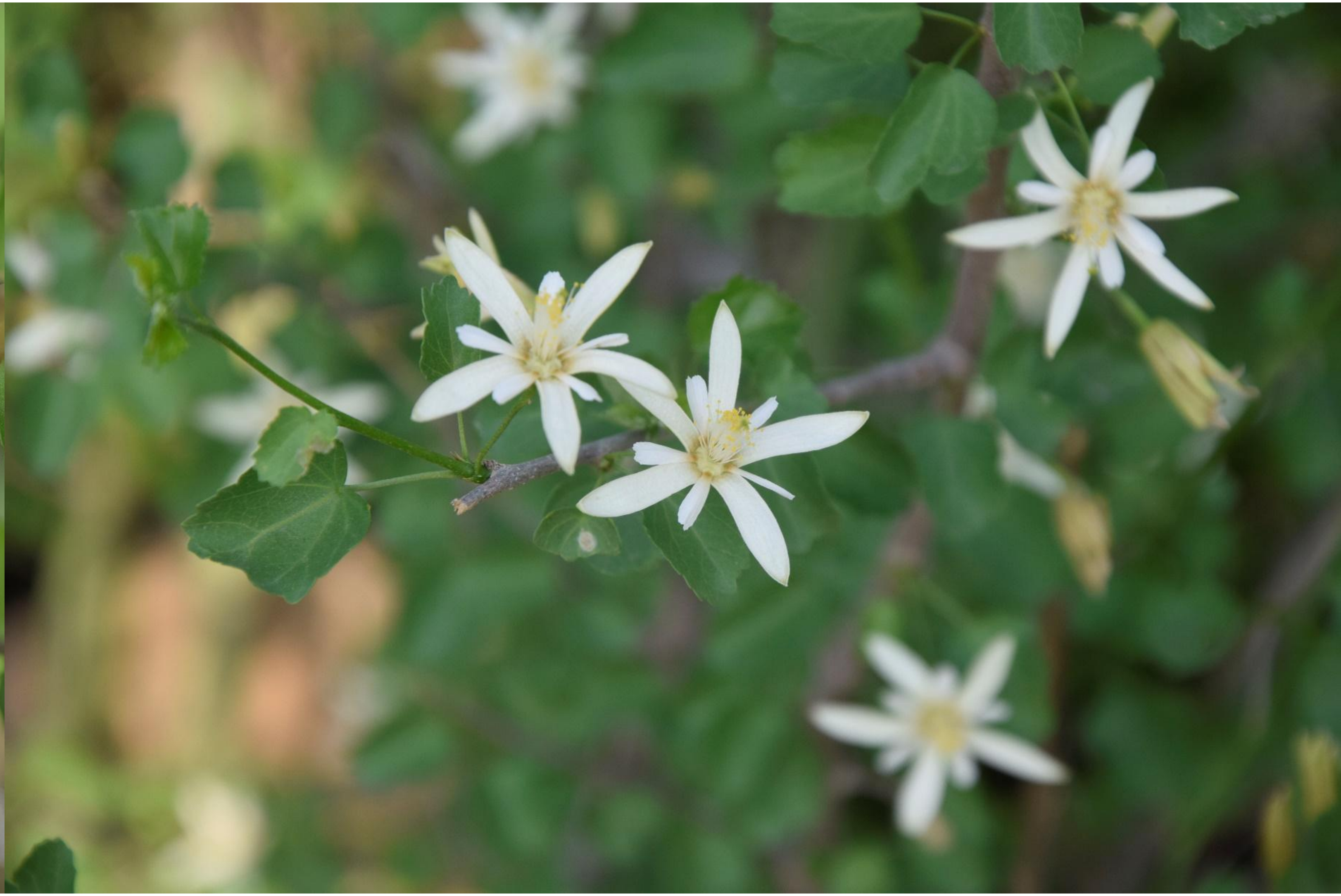

Heliotropium arbainens

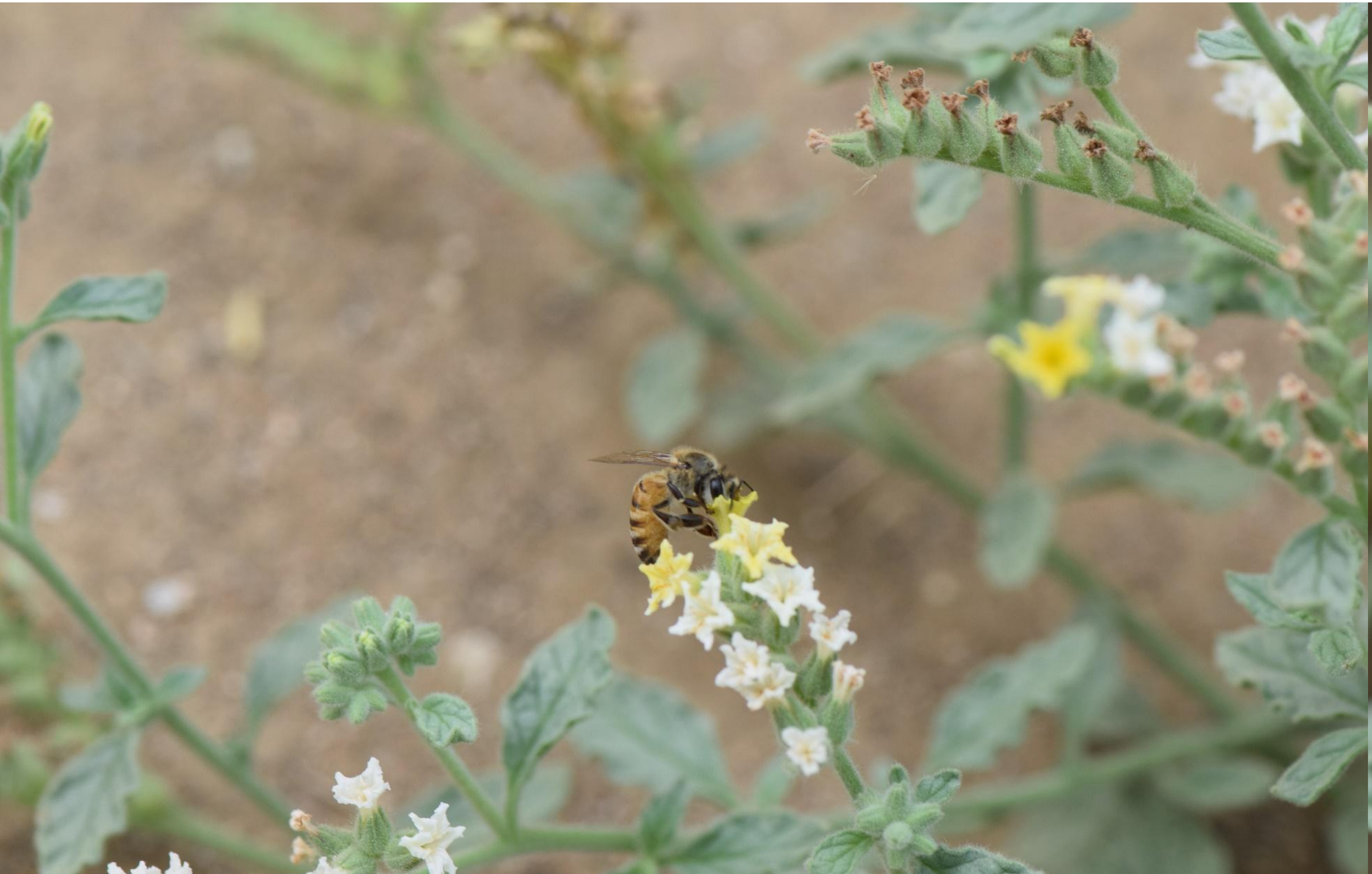

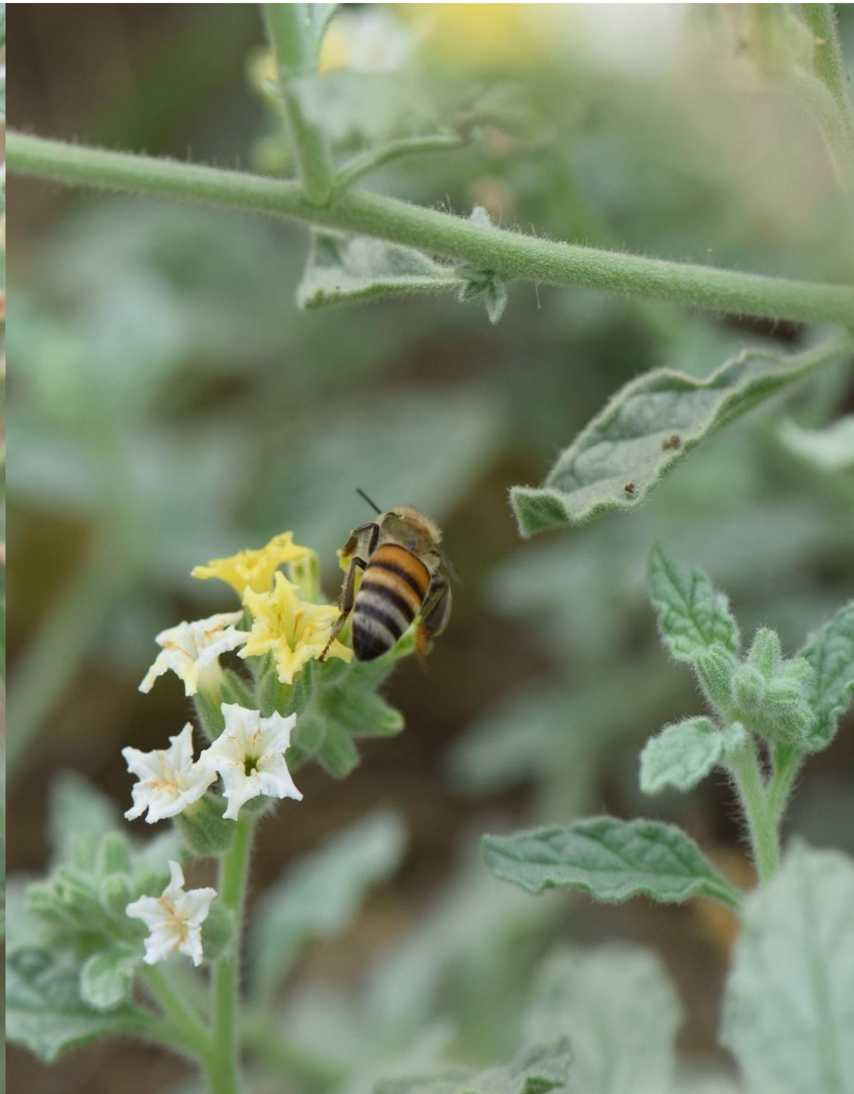

Heliotropium pterocarpum

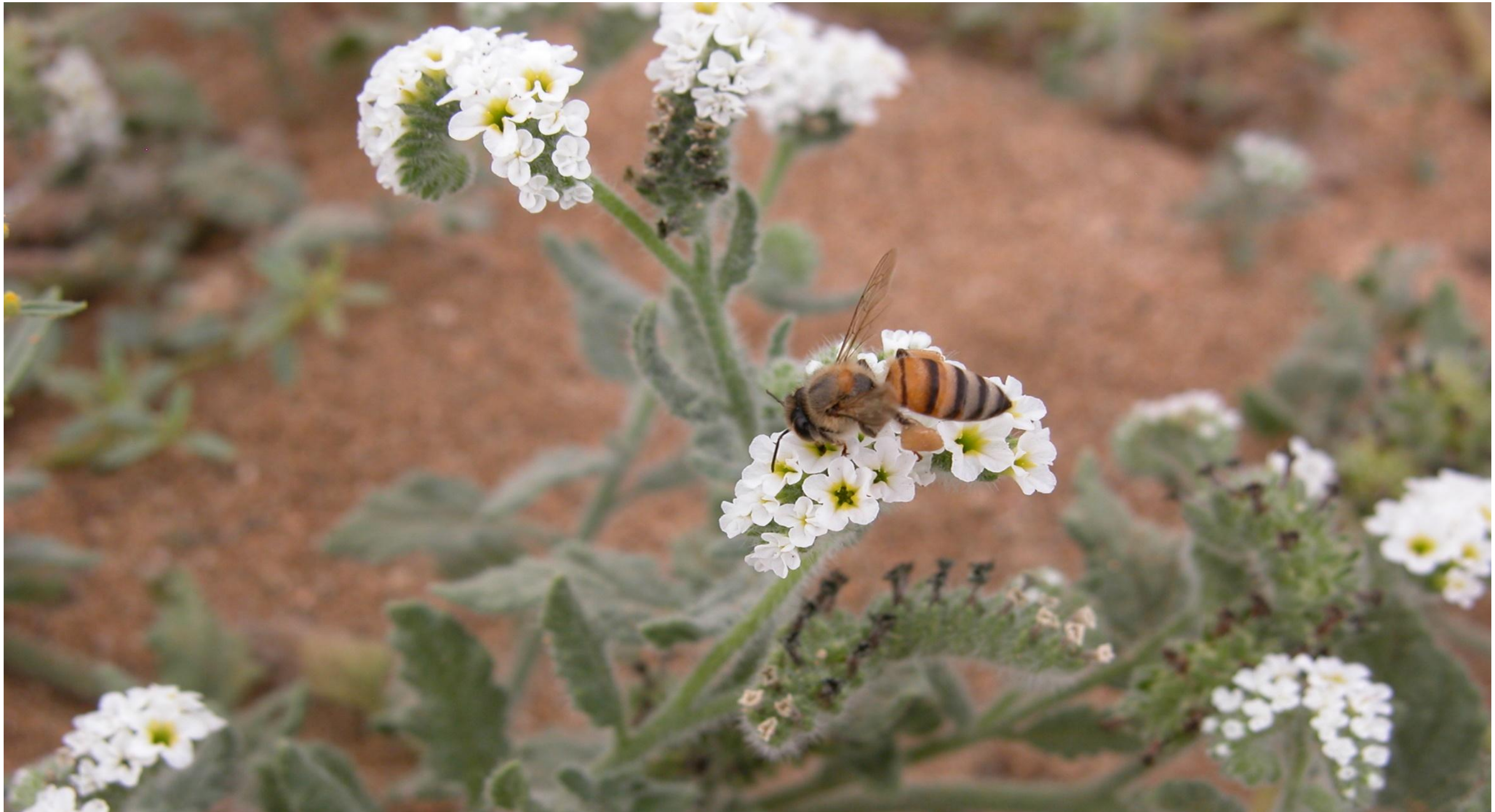

Hypoestes forskaolii

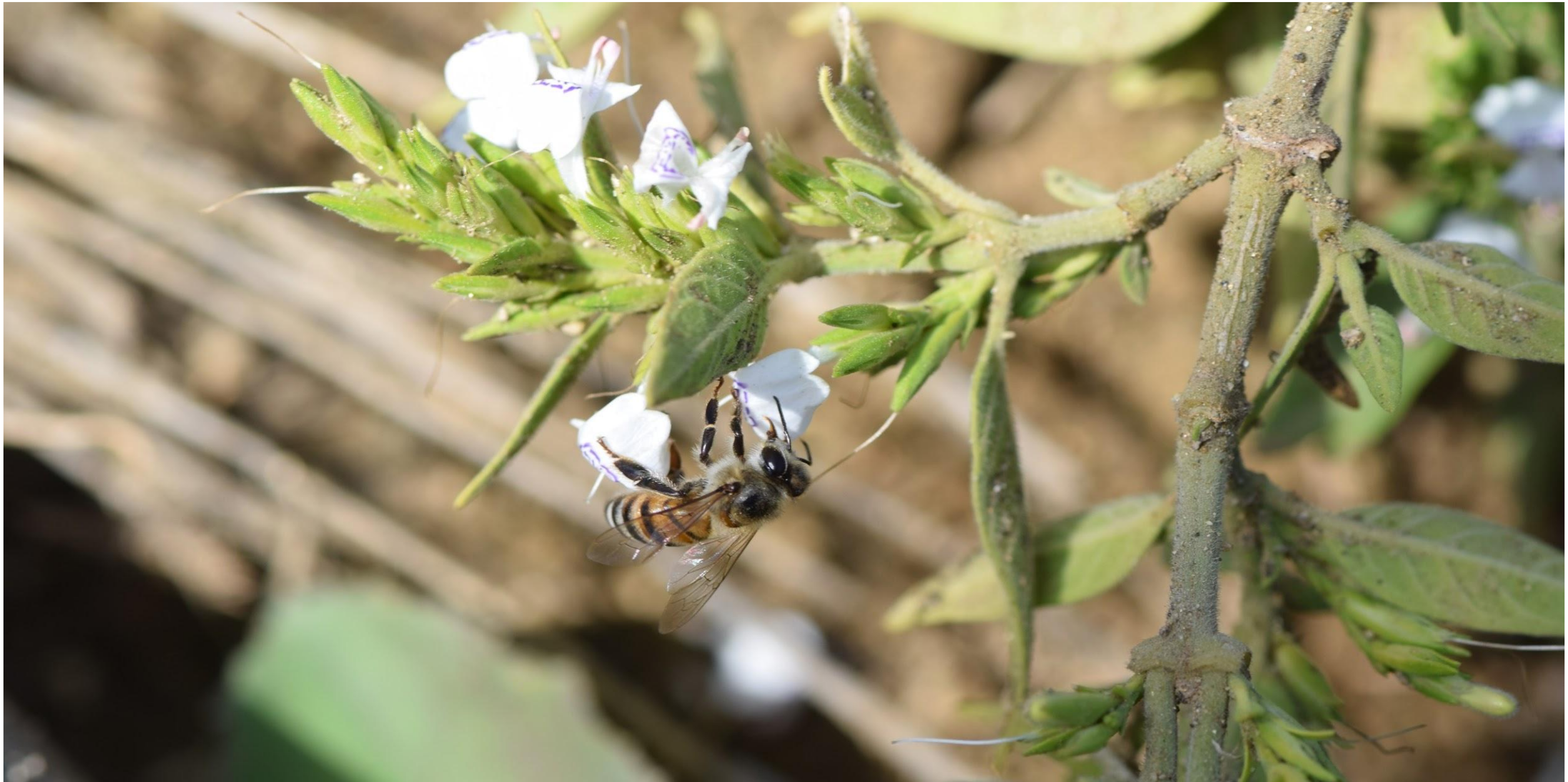

Justicia flava

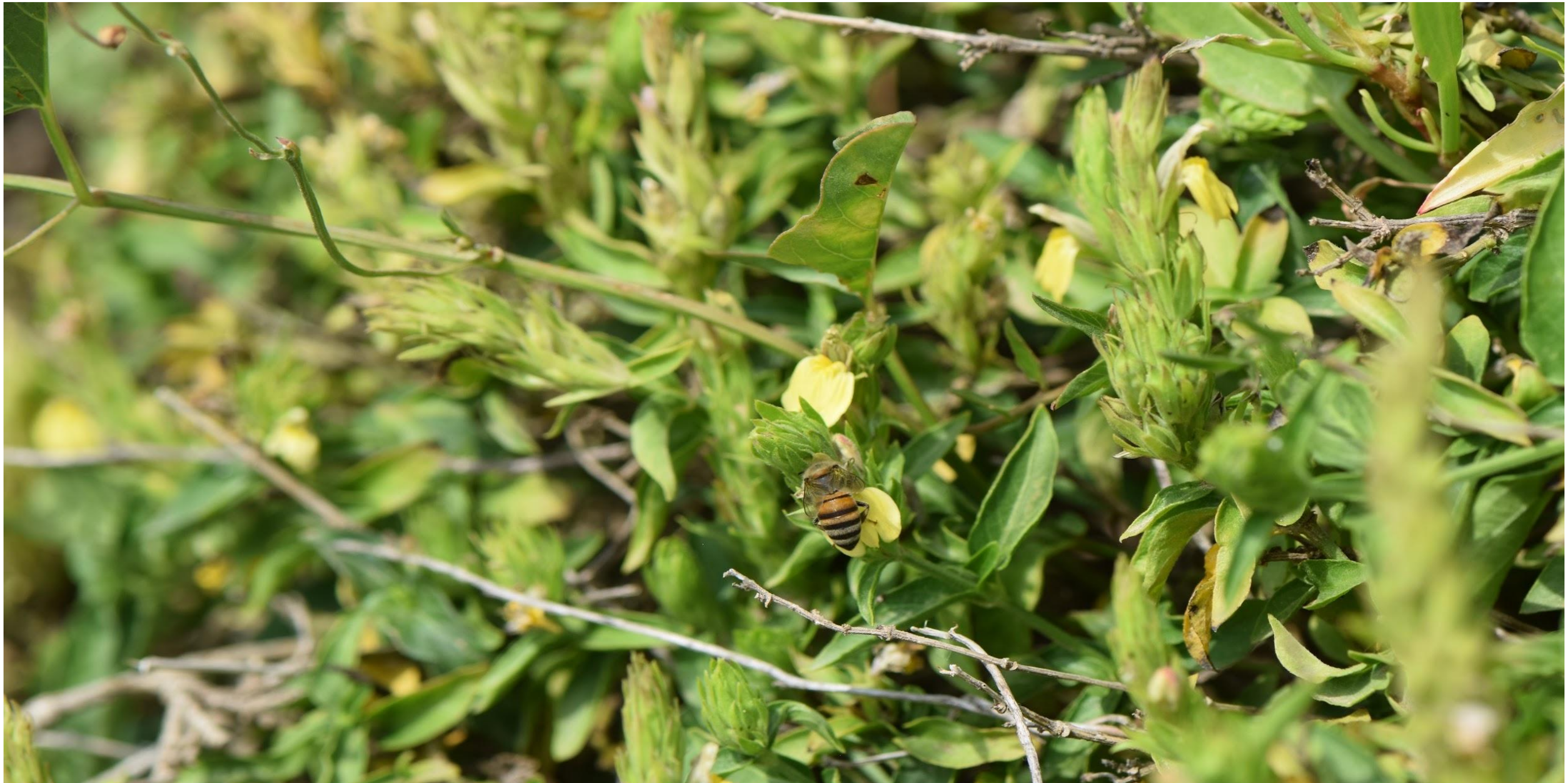

Lactuca serriola

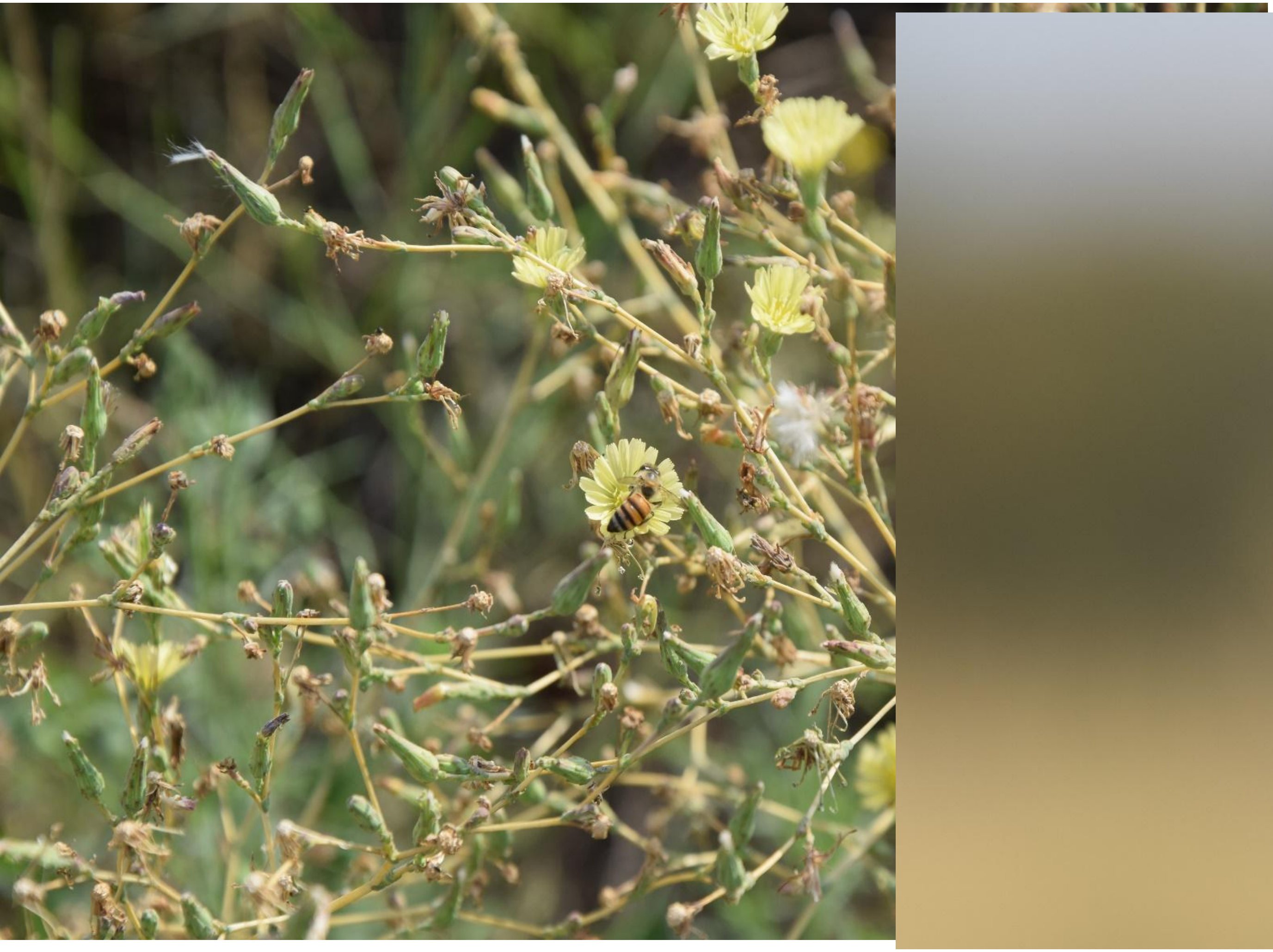

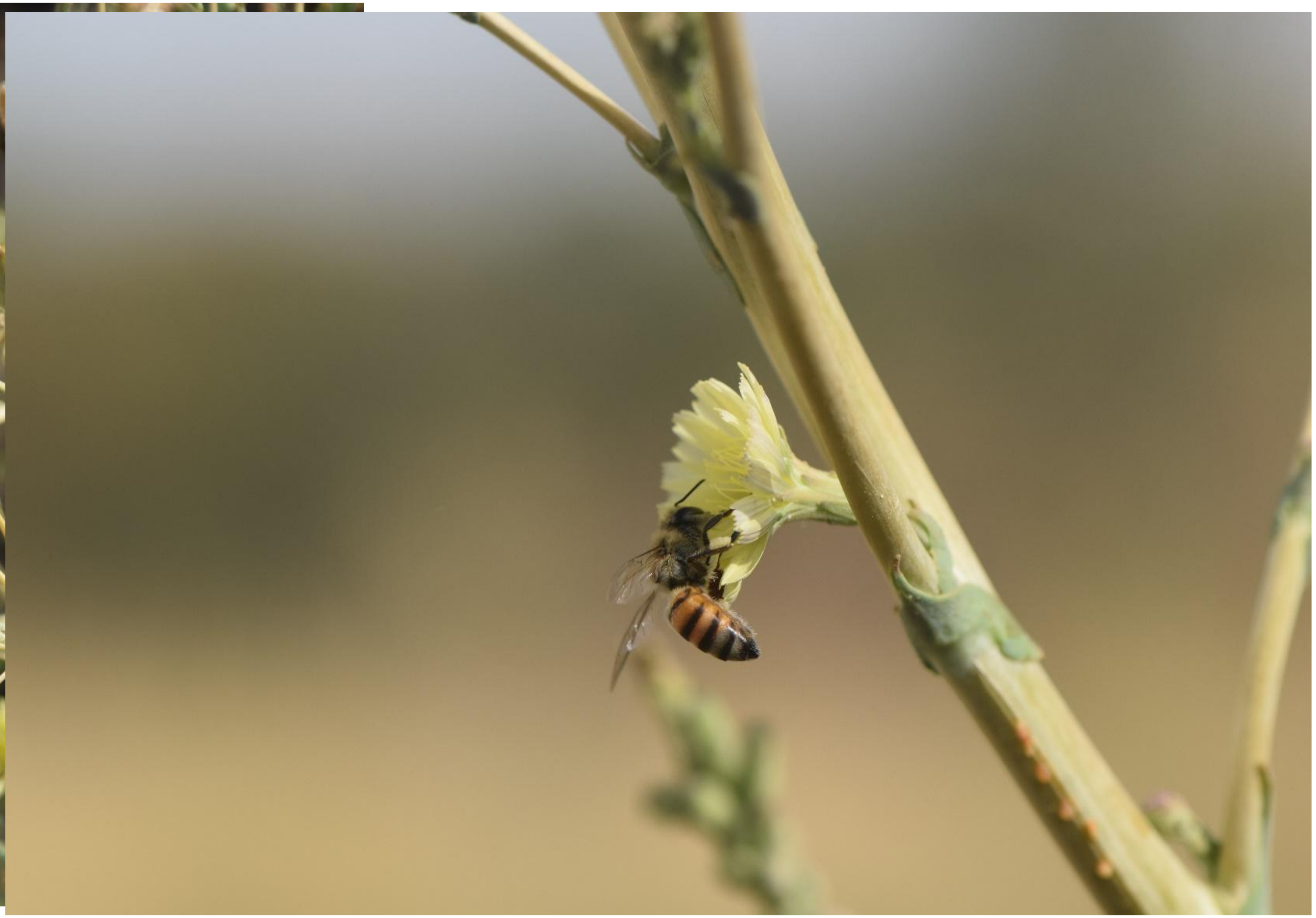

Lavandula dentate

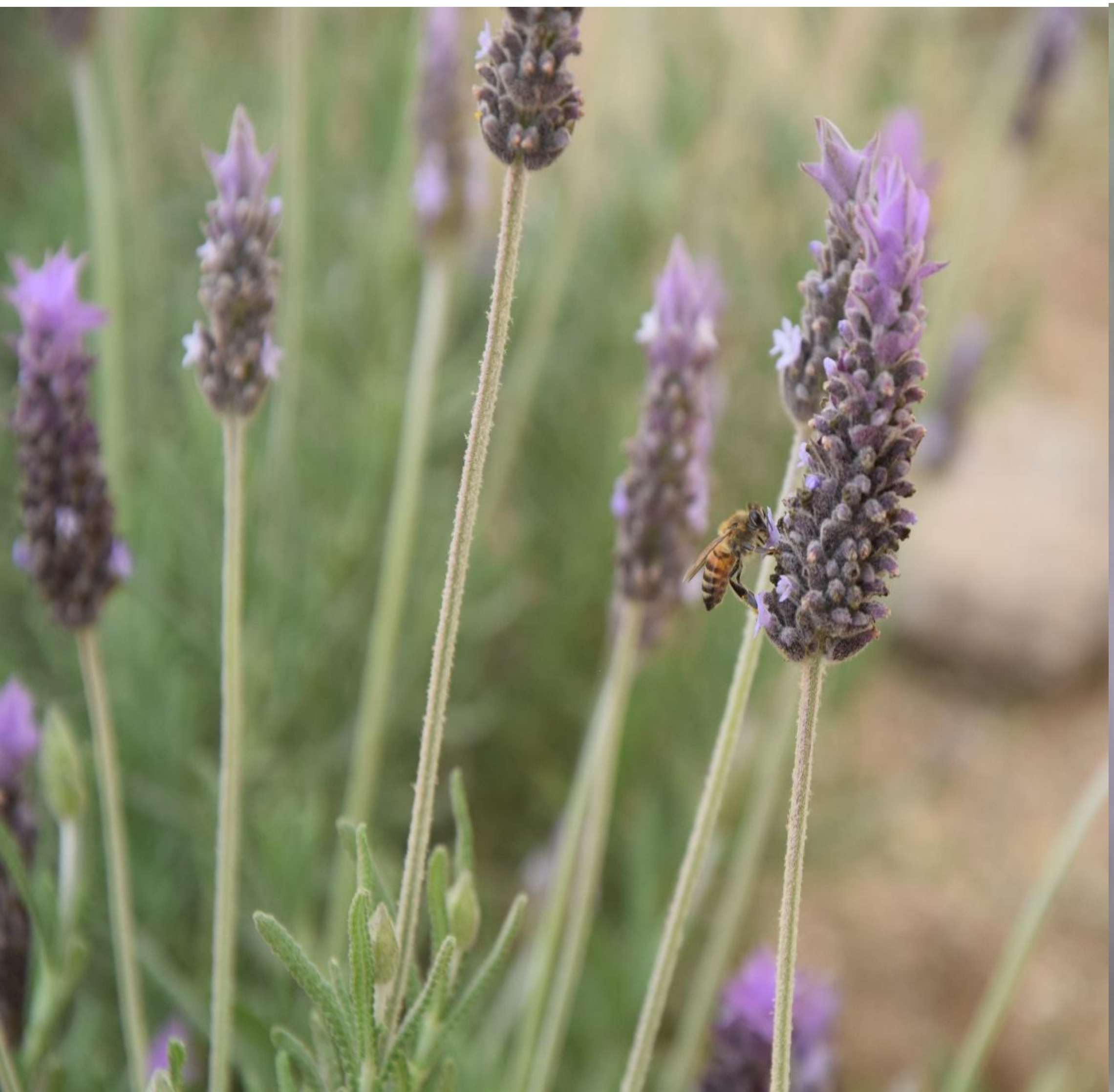

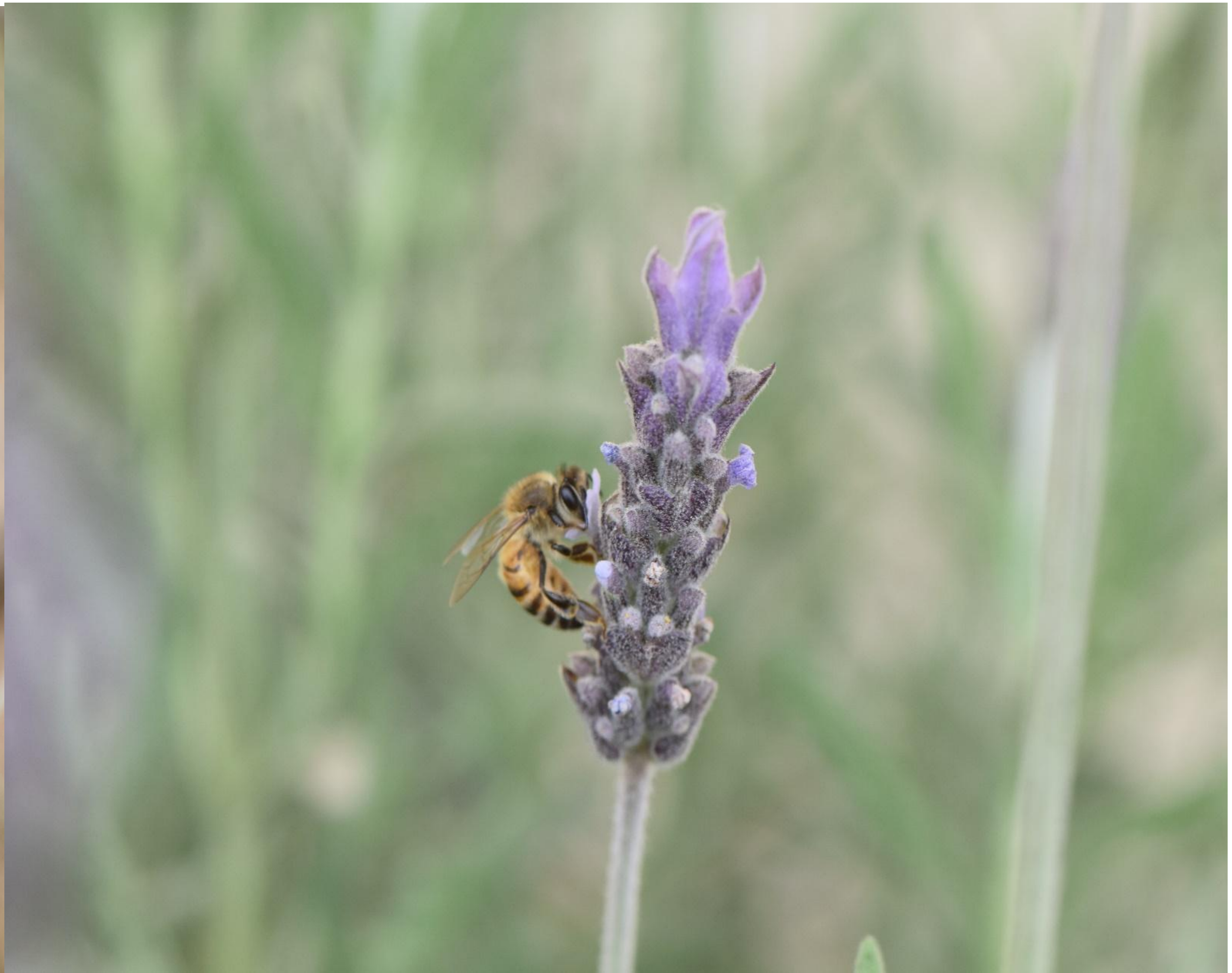

Lavandula pubescens

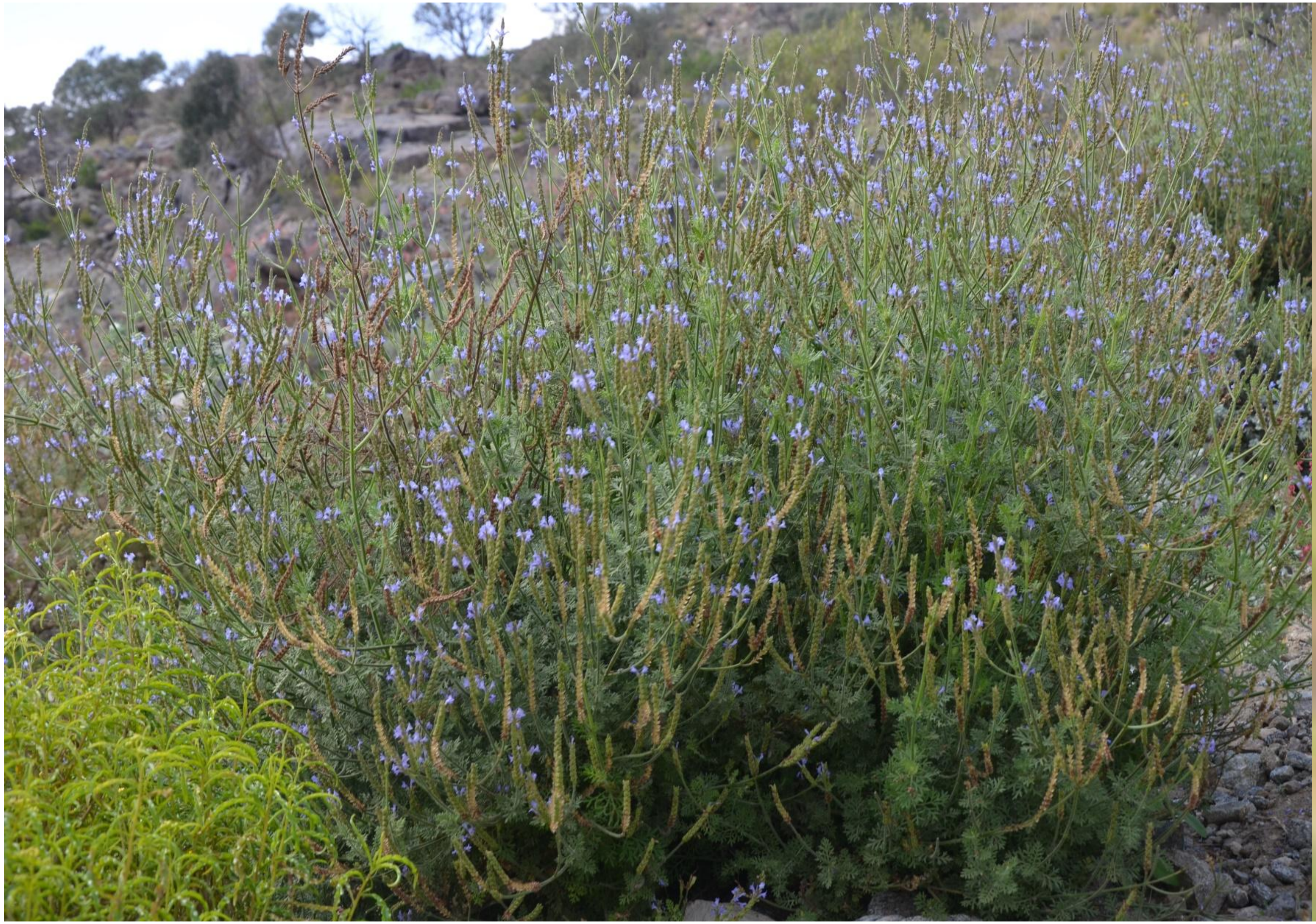

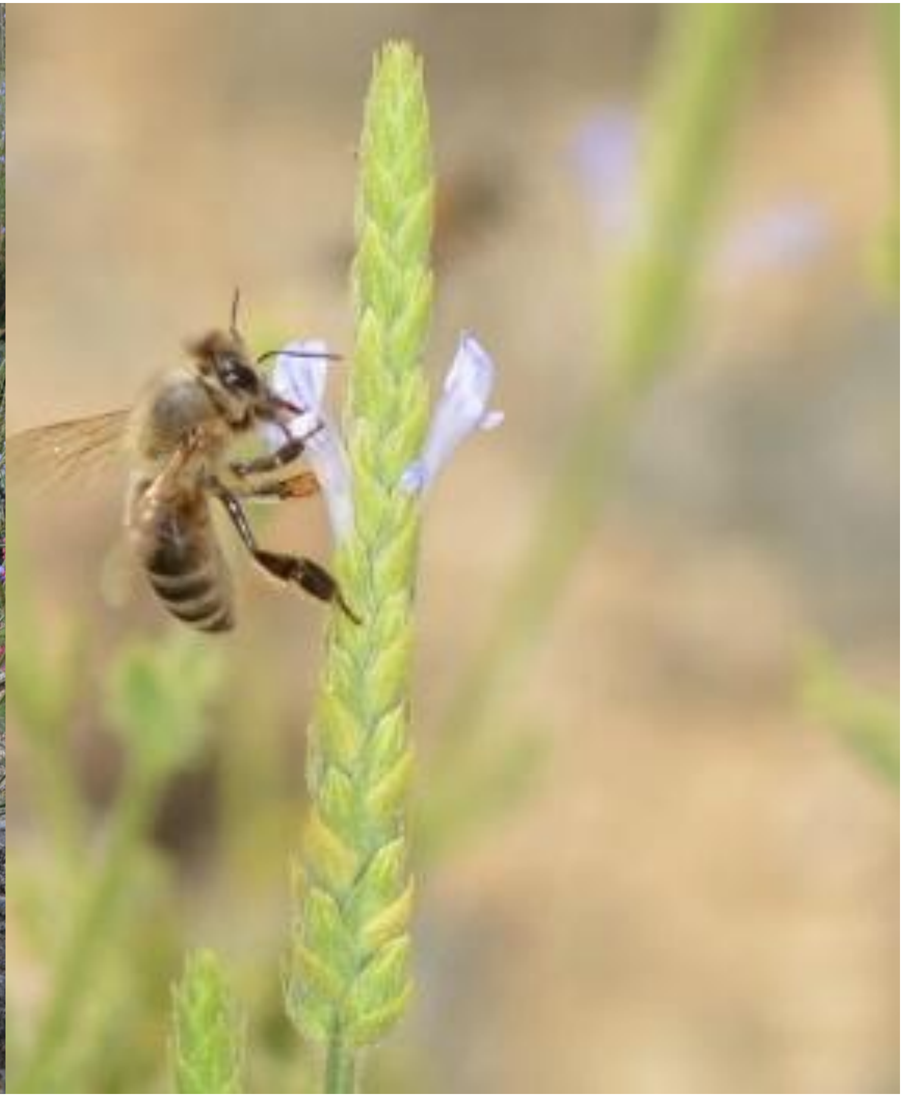

Leptadenia pyrotechnica

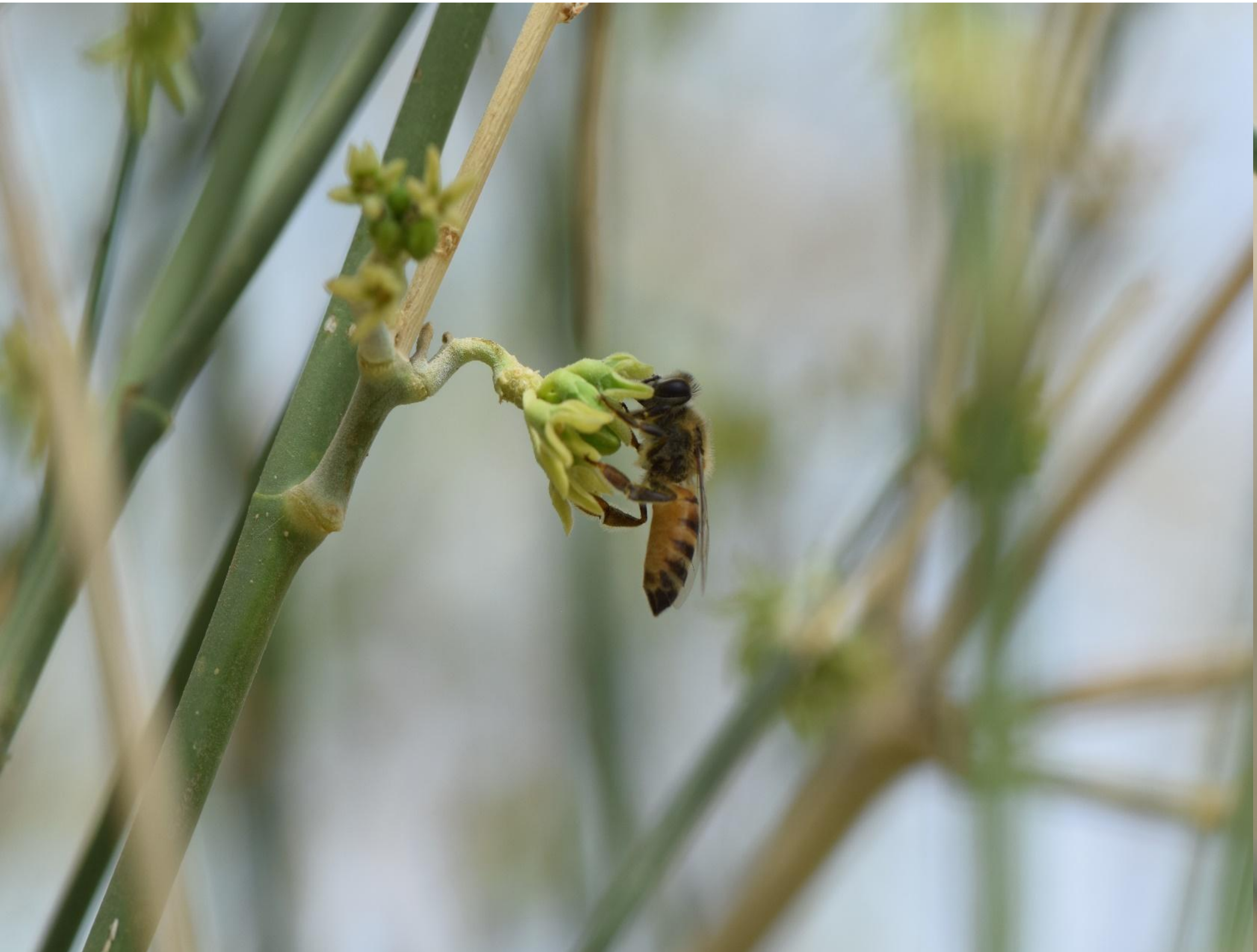

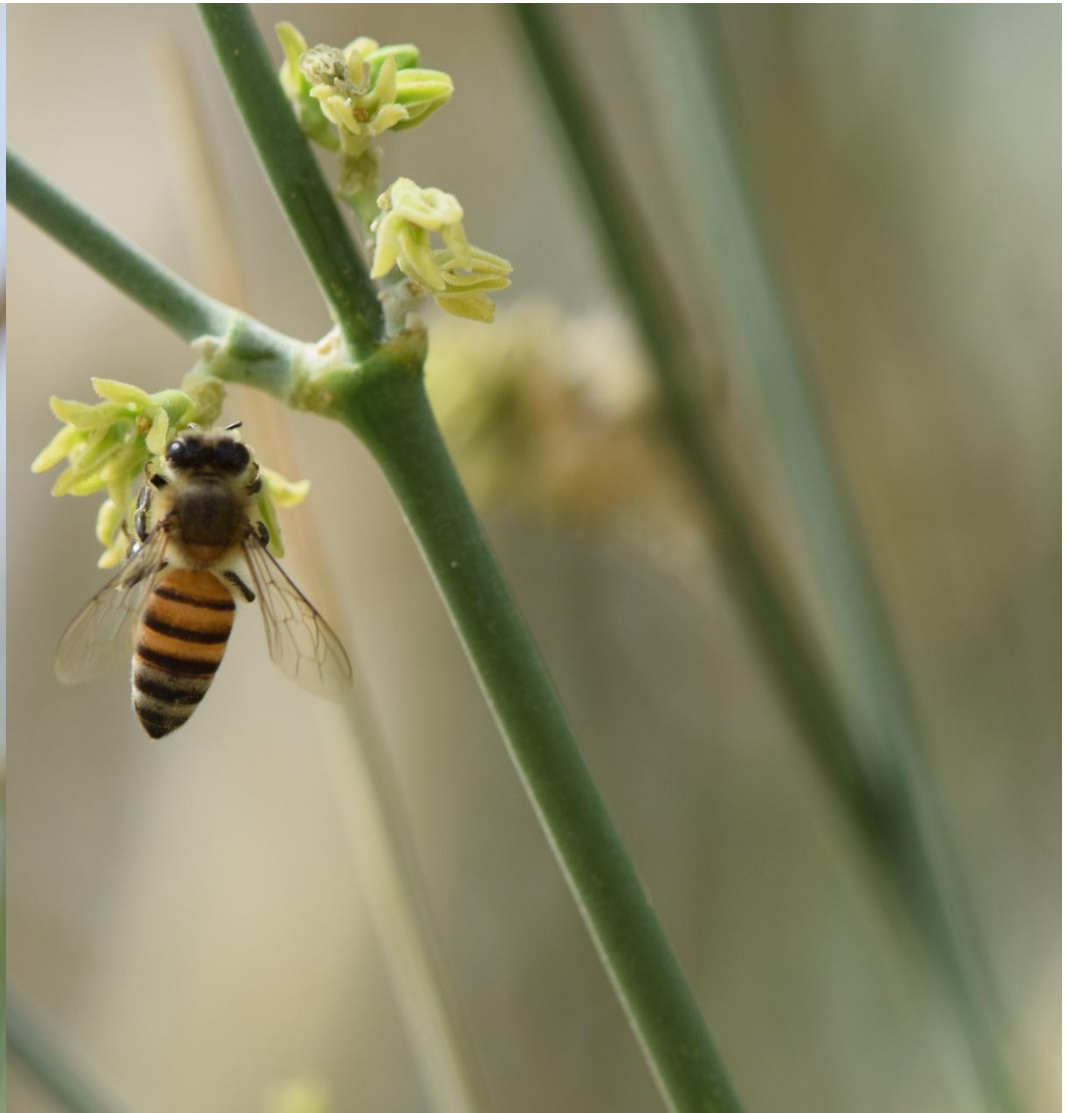

Lotus quinatus

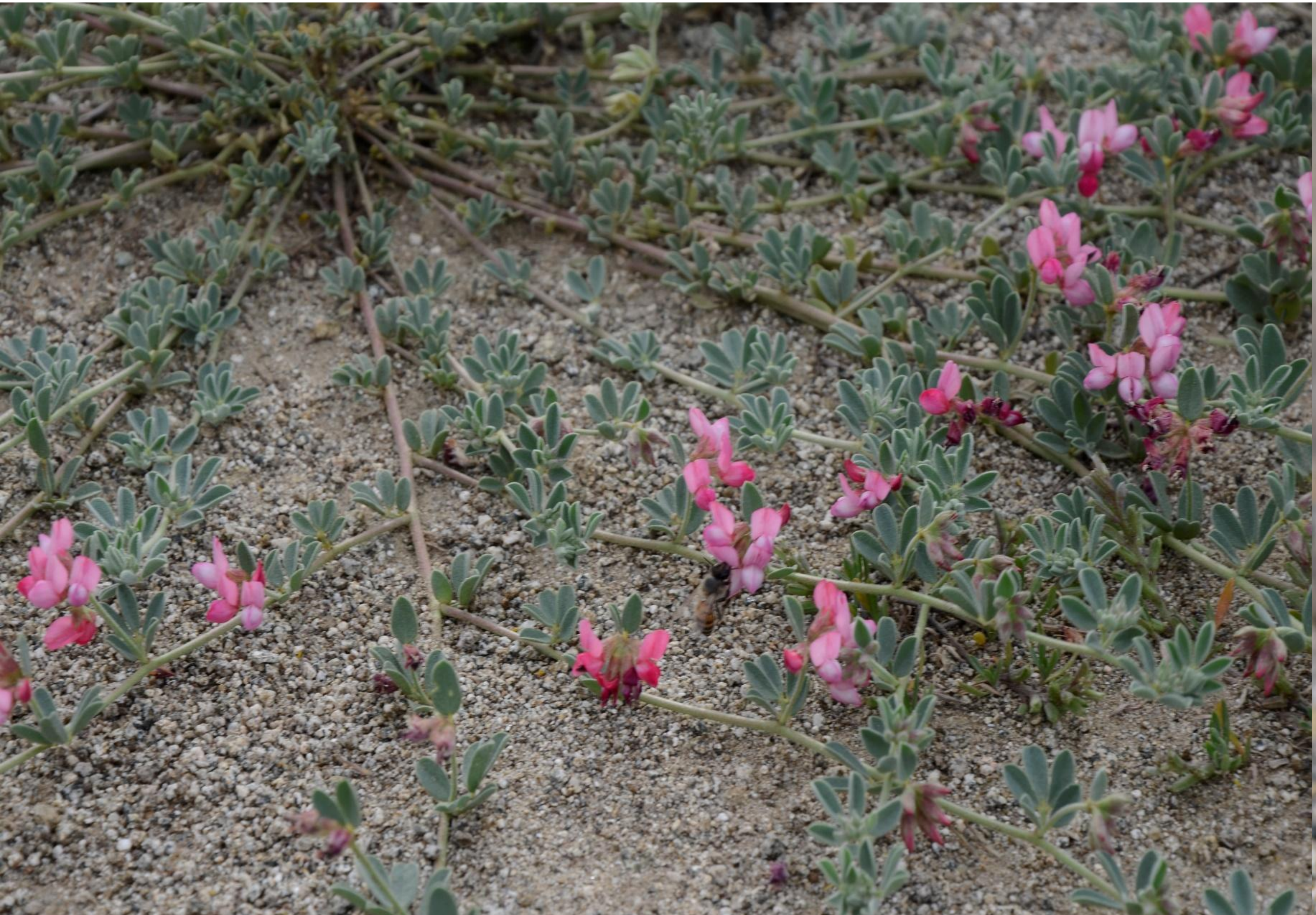

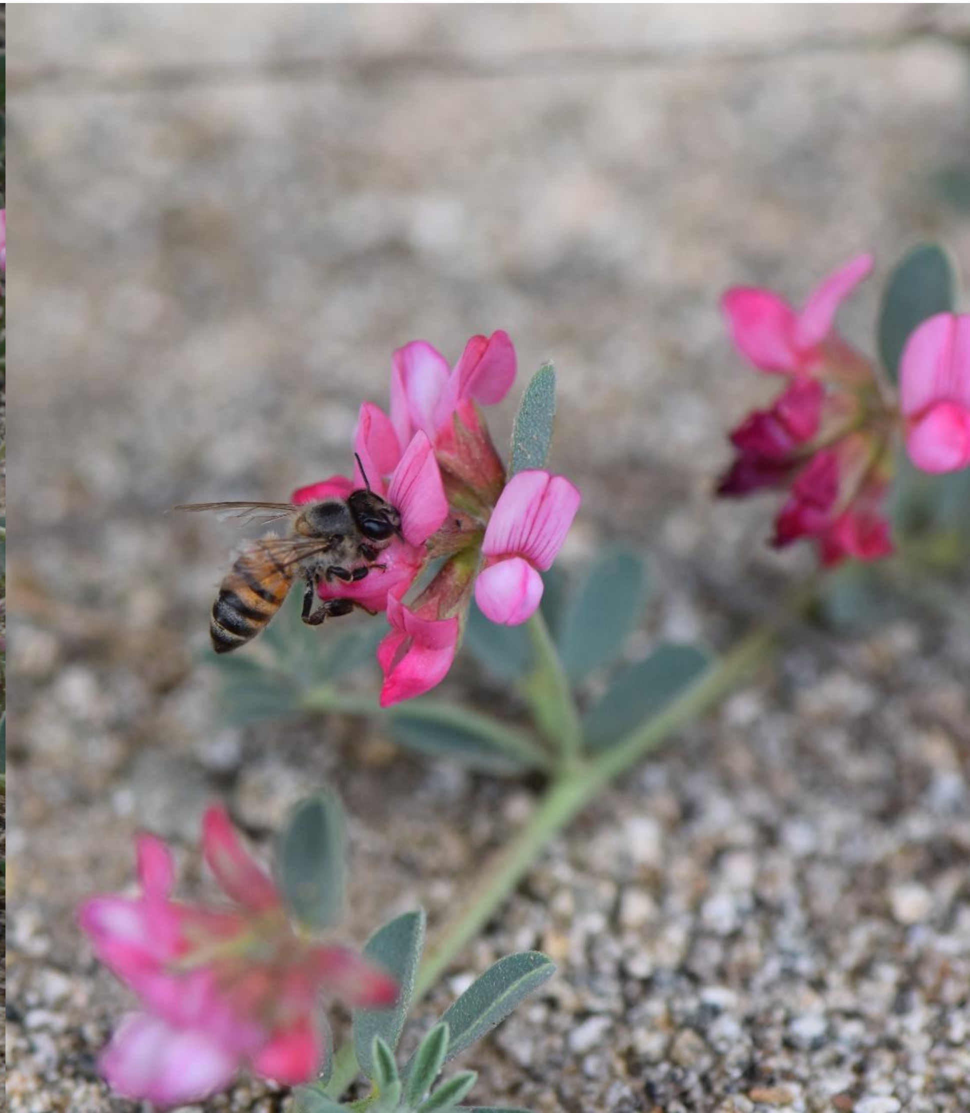

Maerue cricifolia

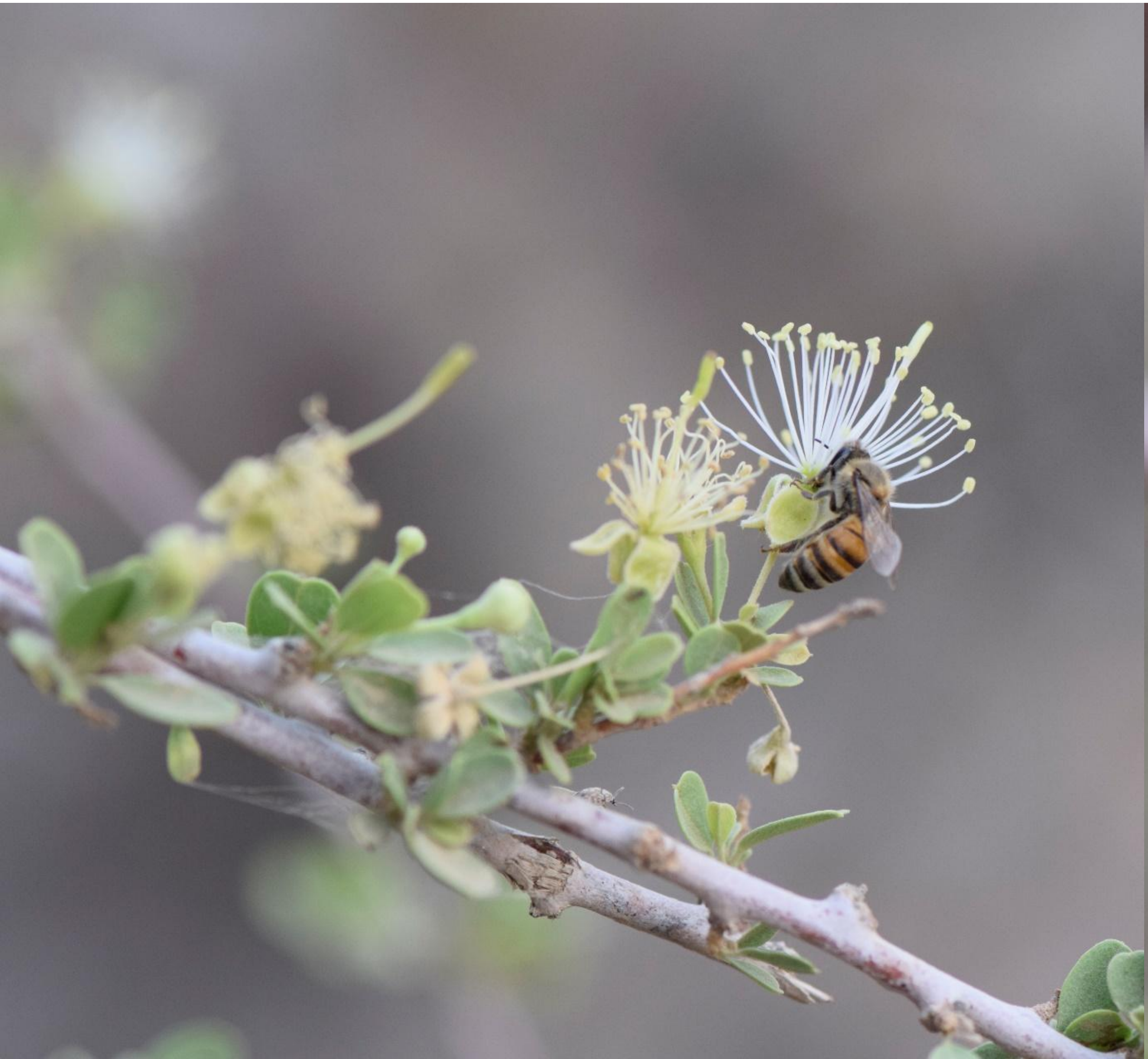

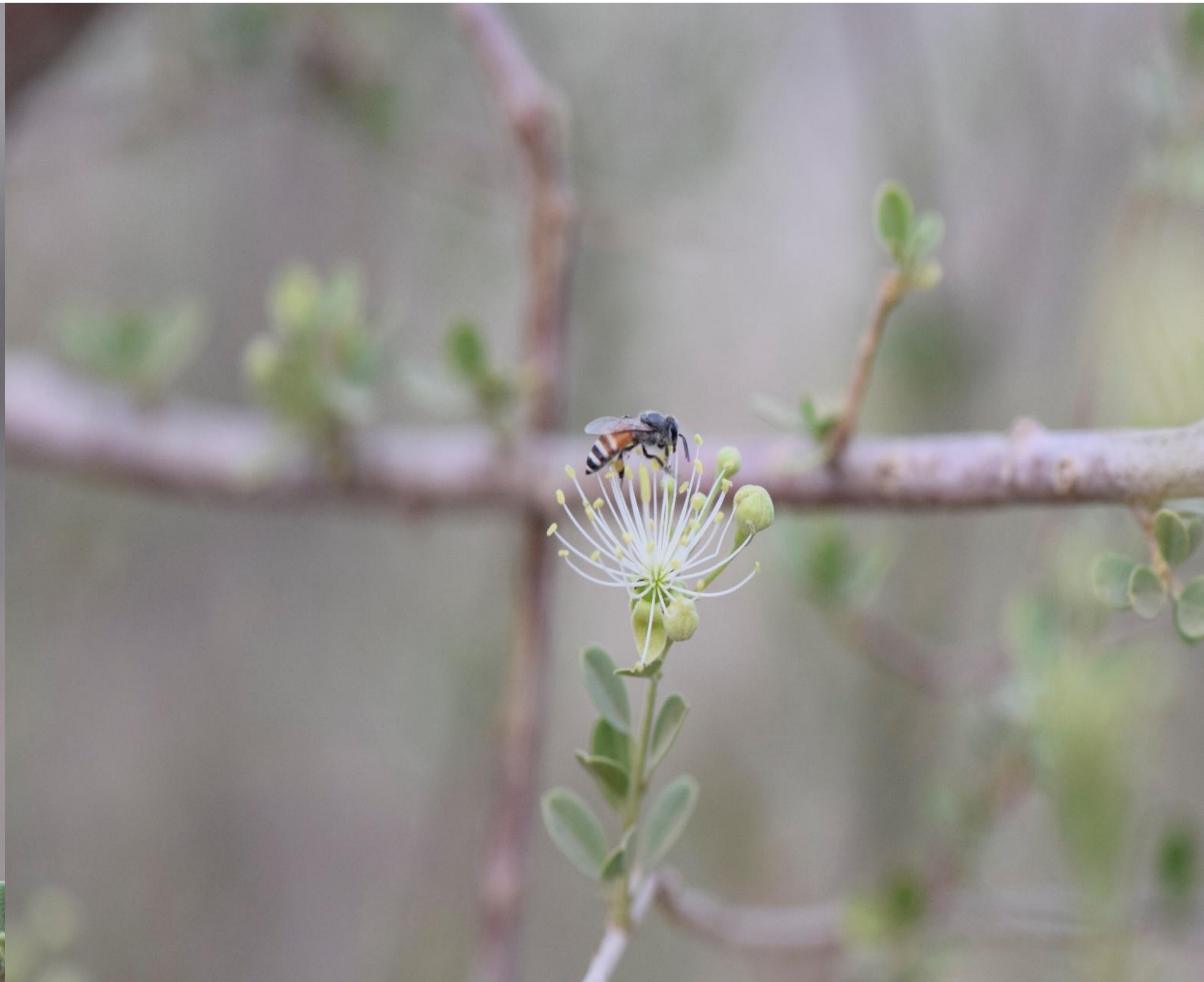

Mentha longifolia

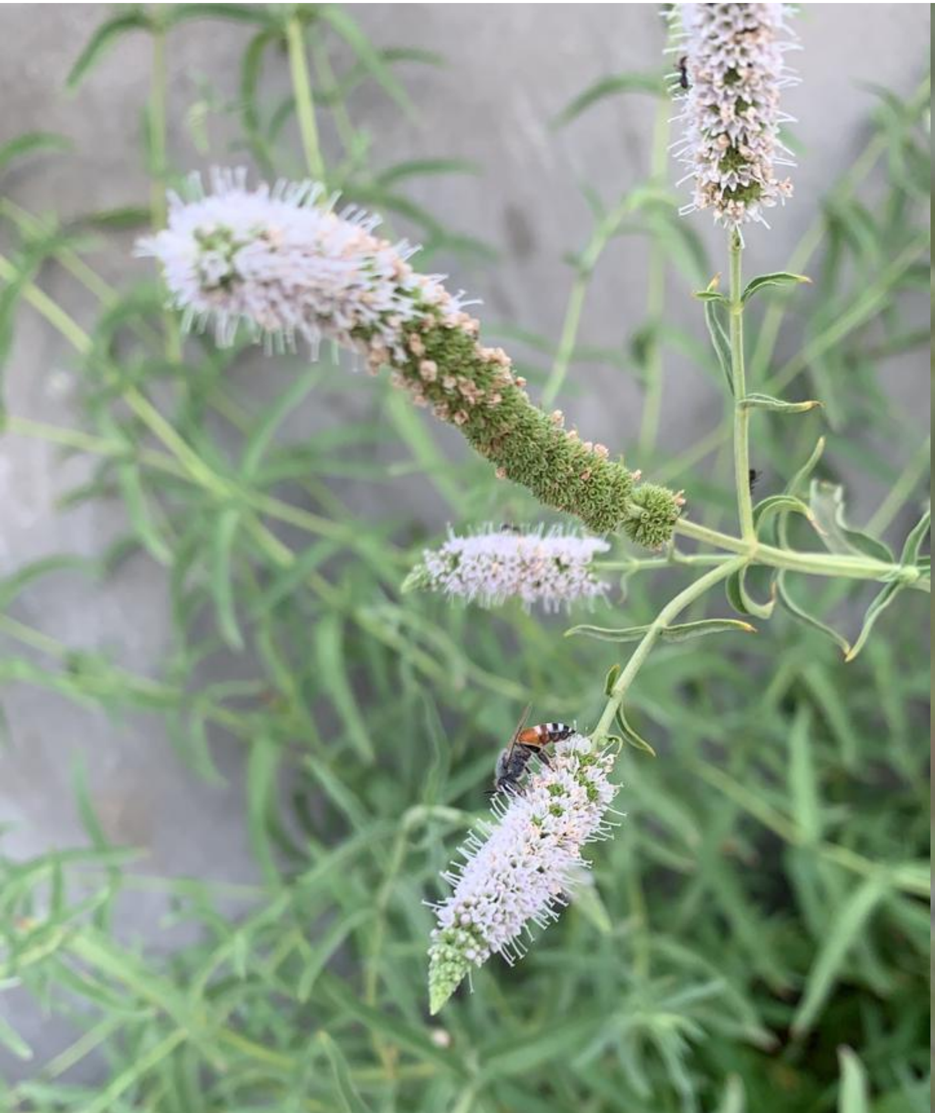

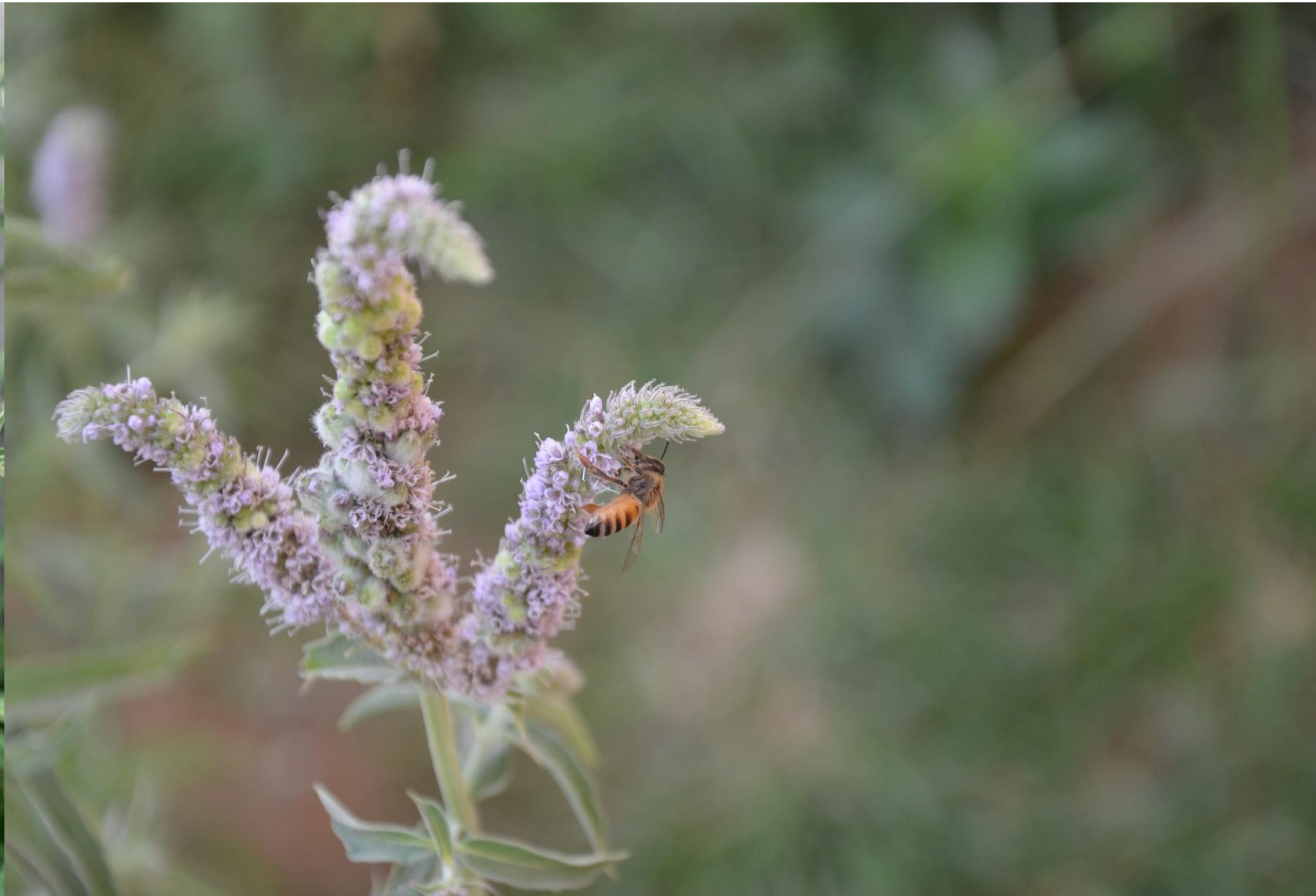

Myrtus communis

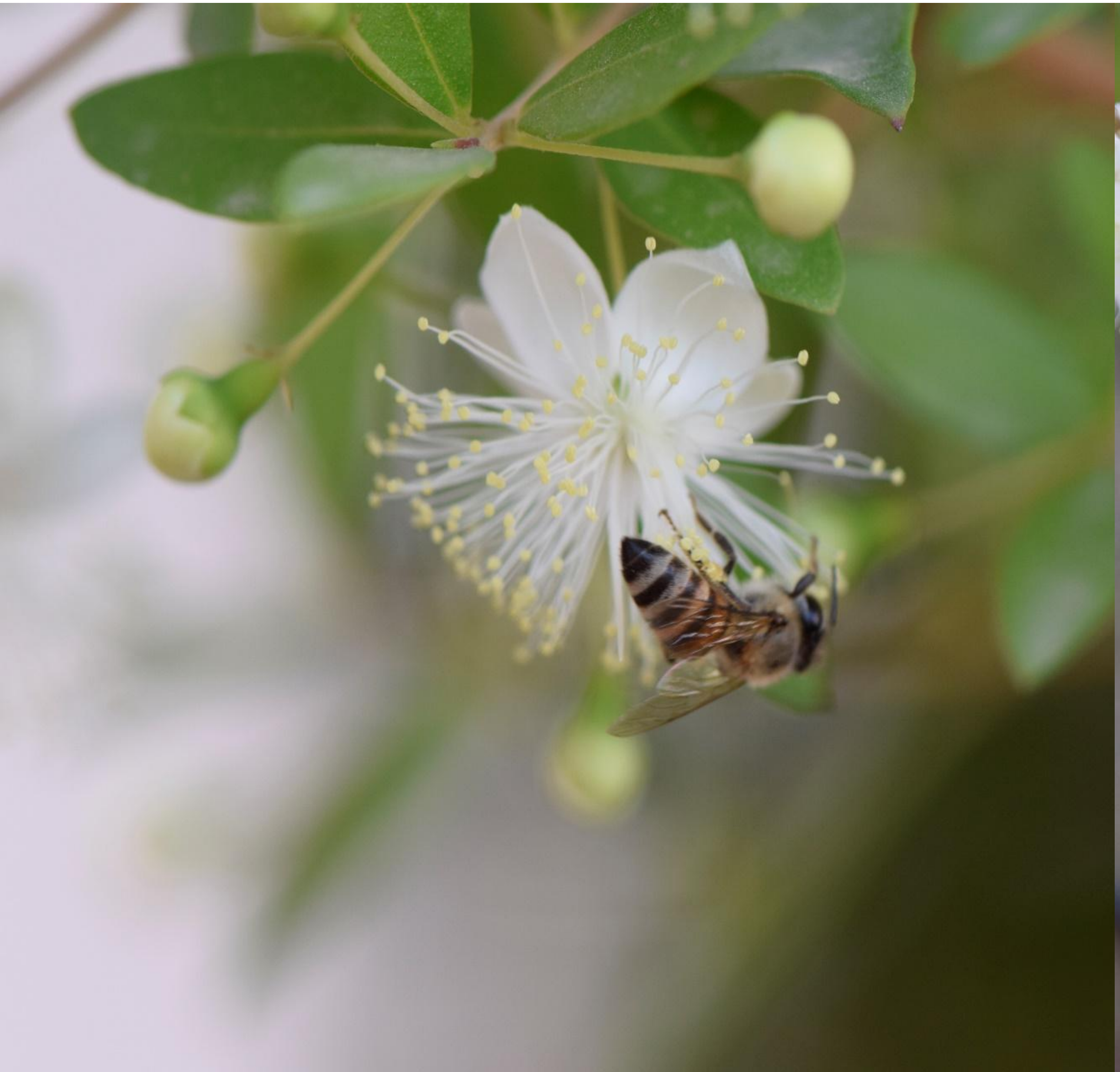

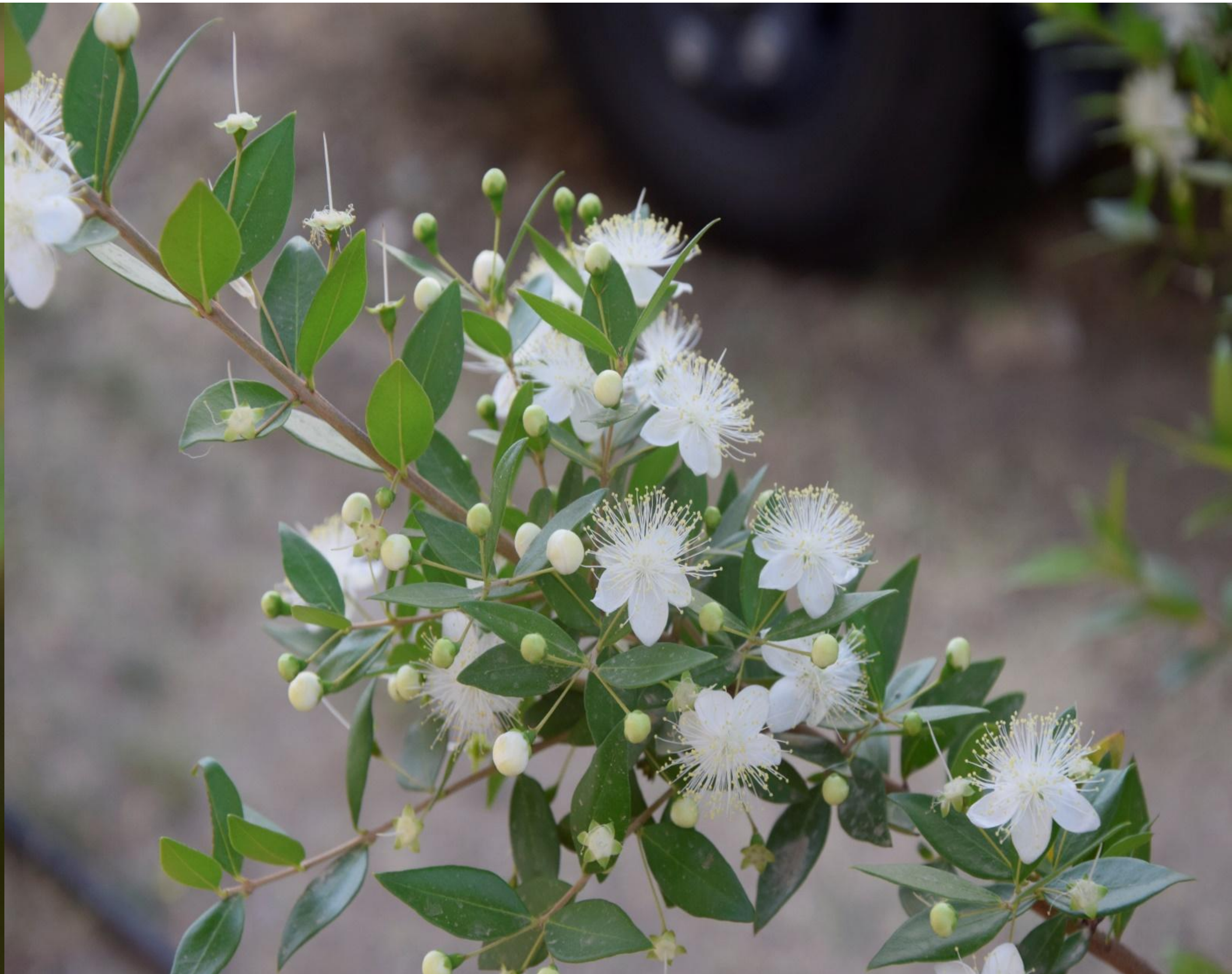

Nepeta deflersiana

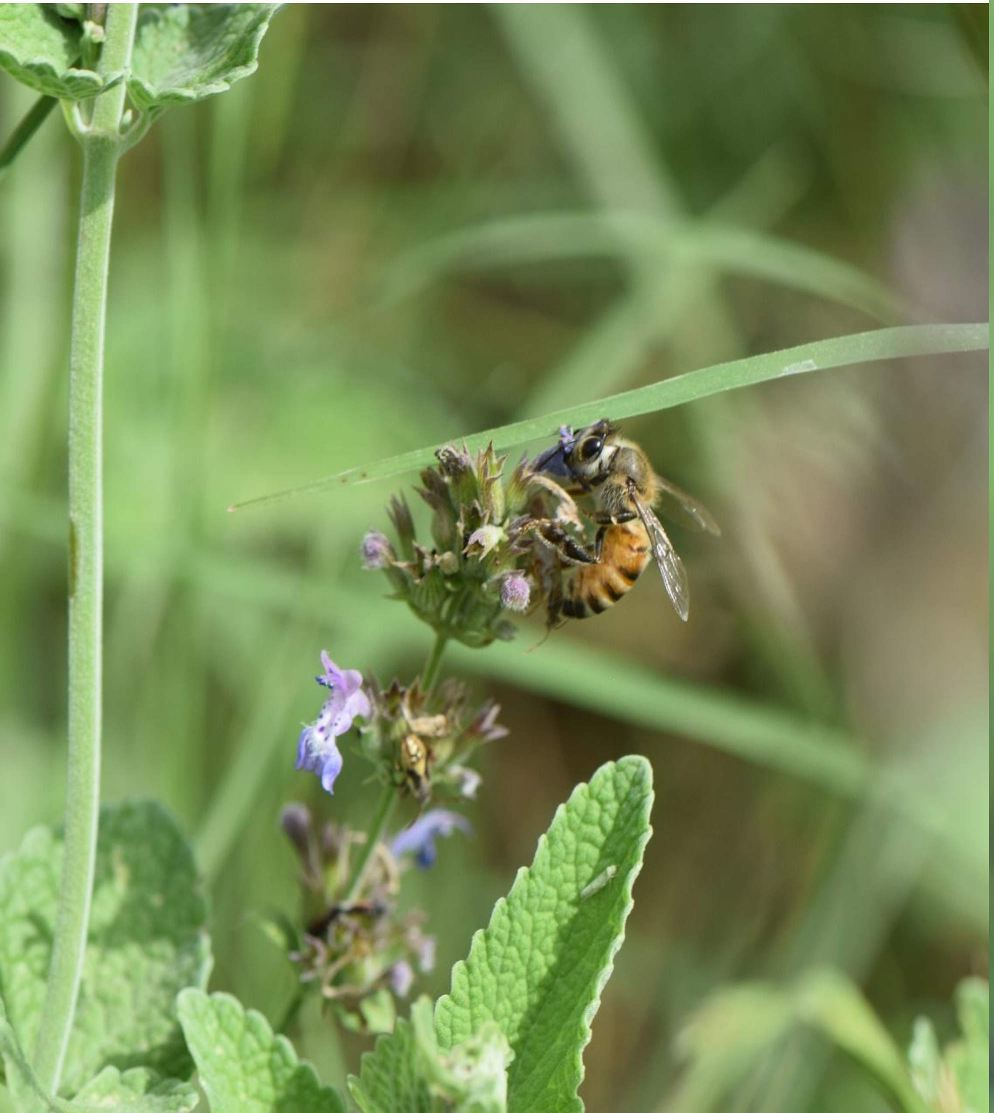

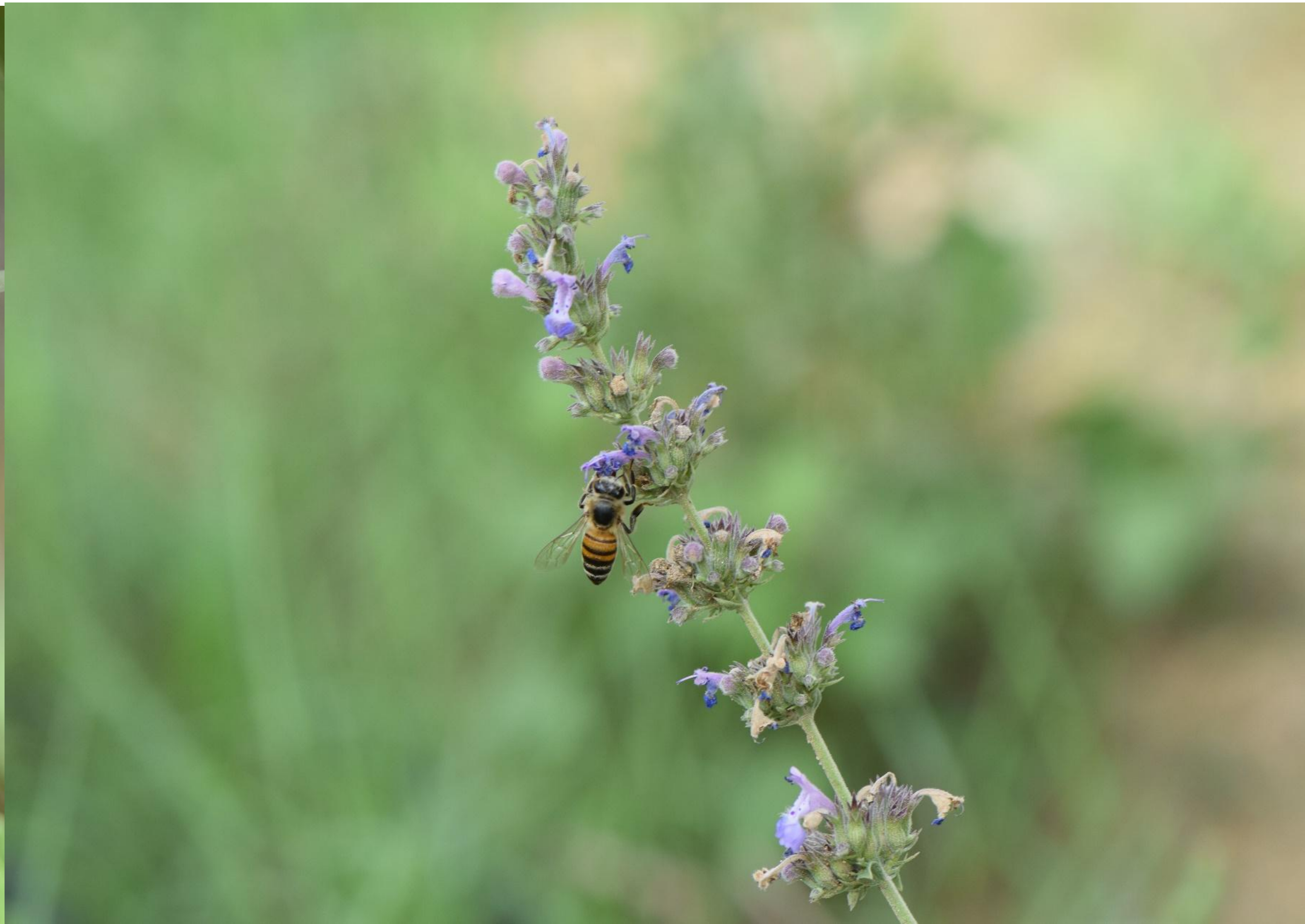

Ochradenus baccatus

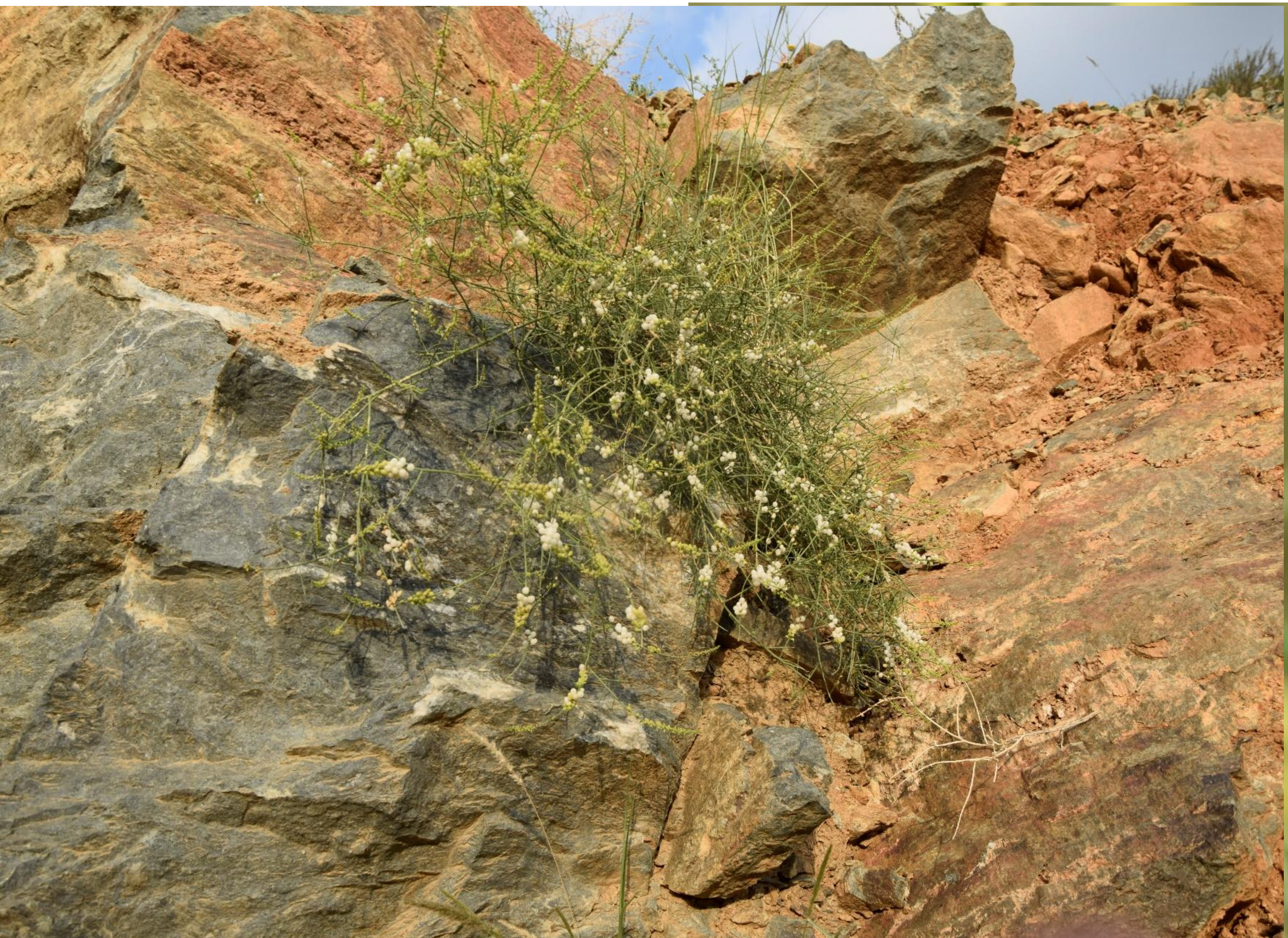
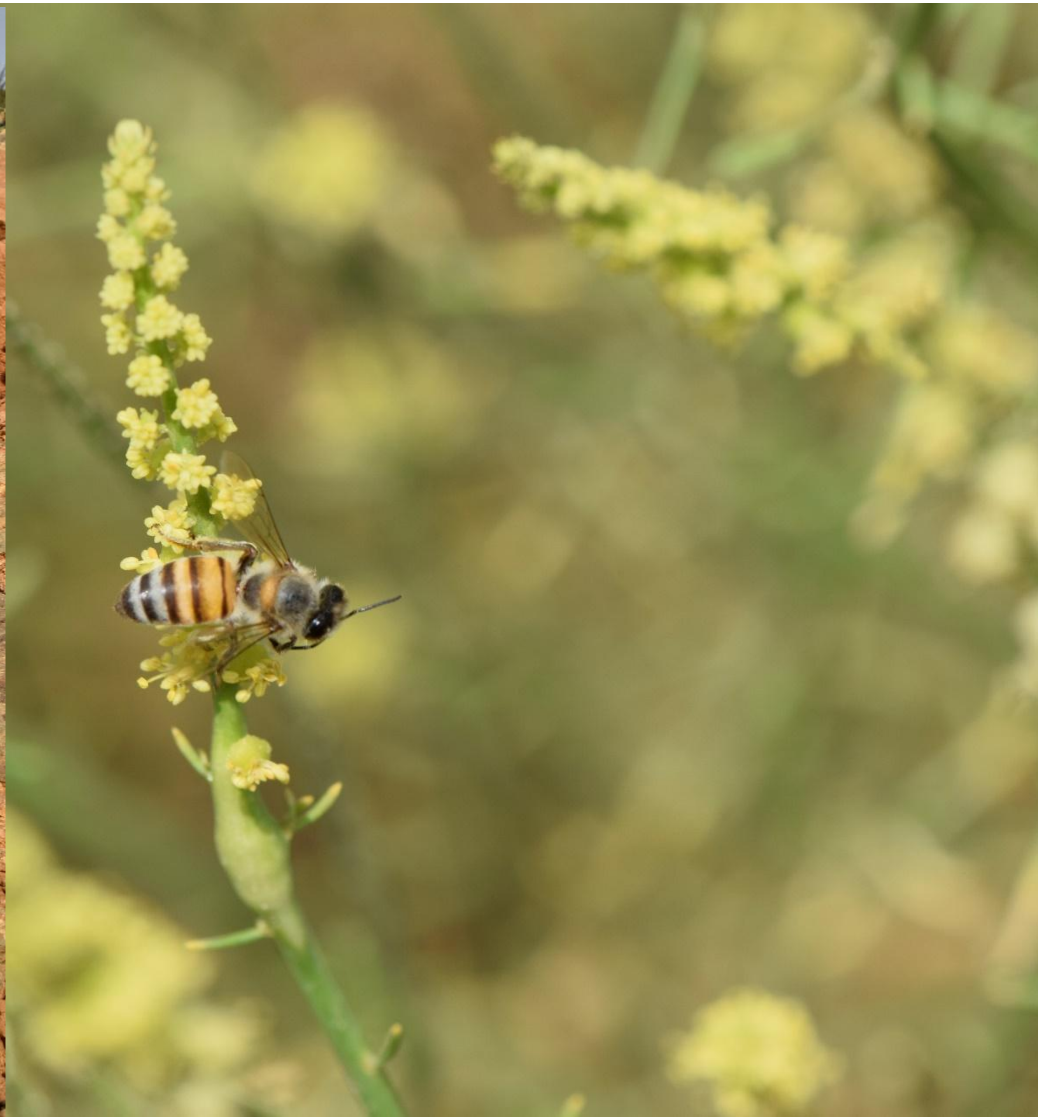

Onobrychis ptolemaica

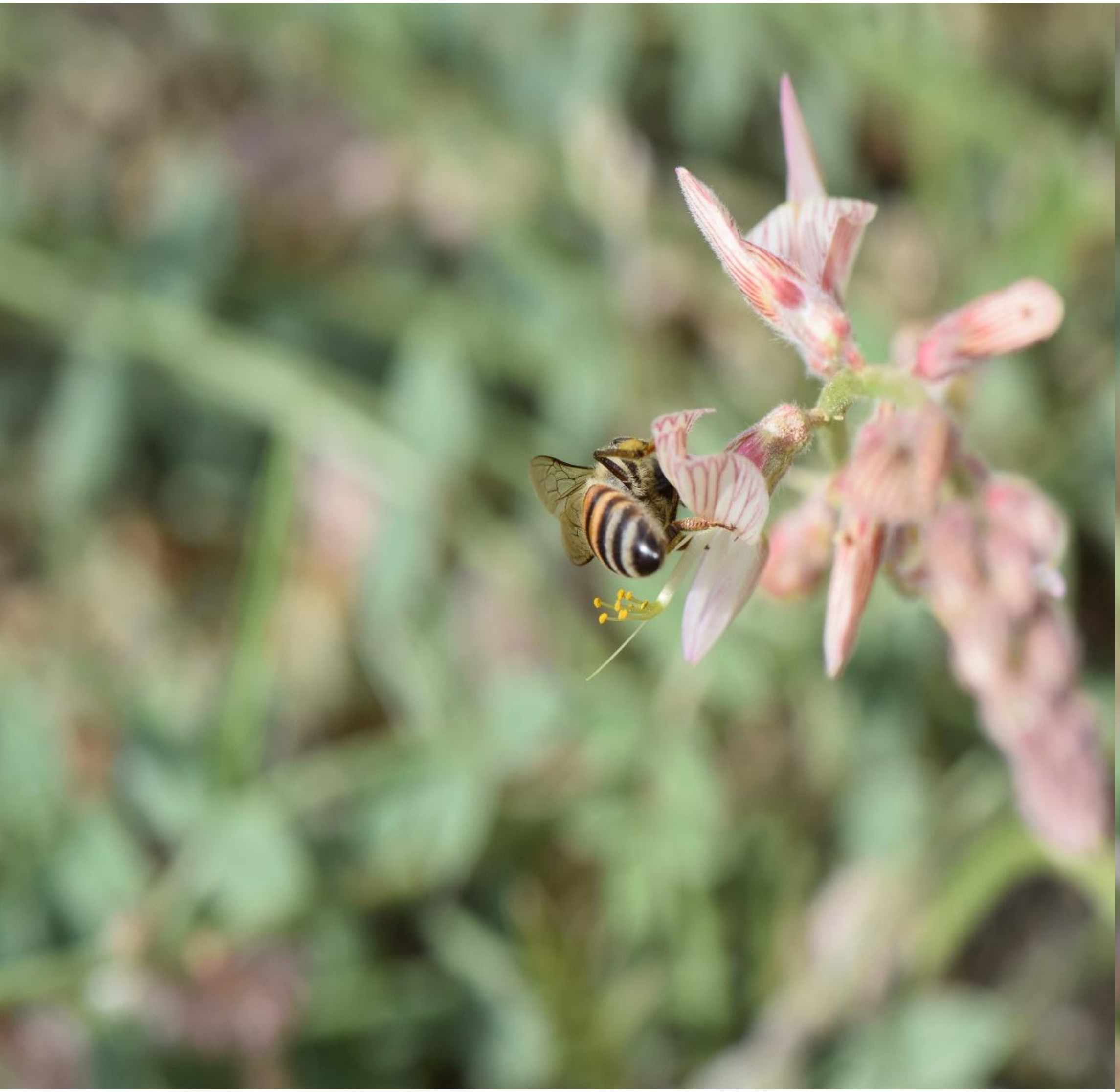

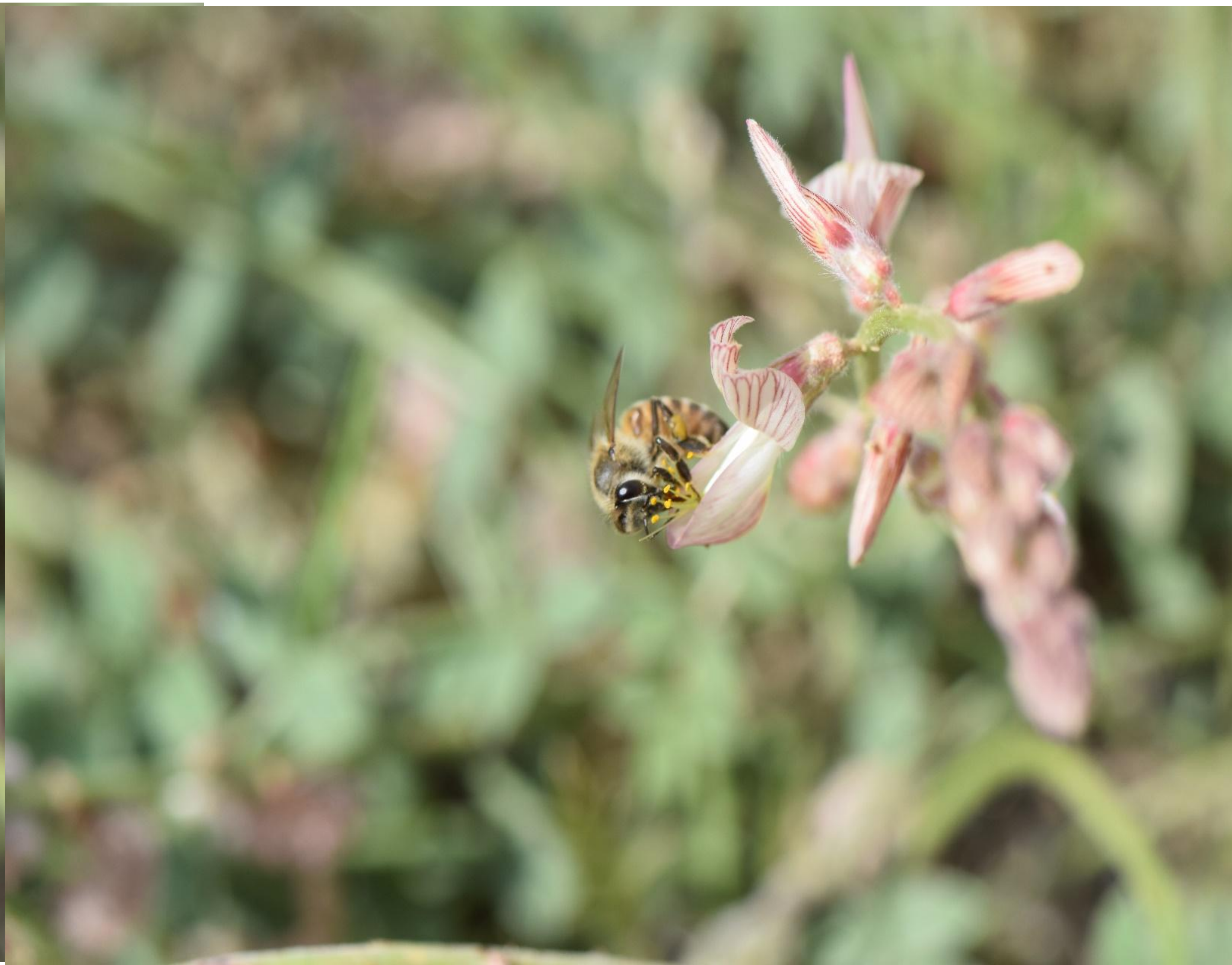

Onopordum heteracanthum

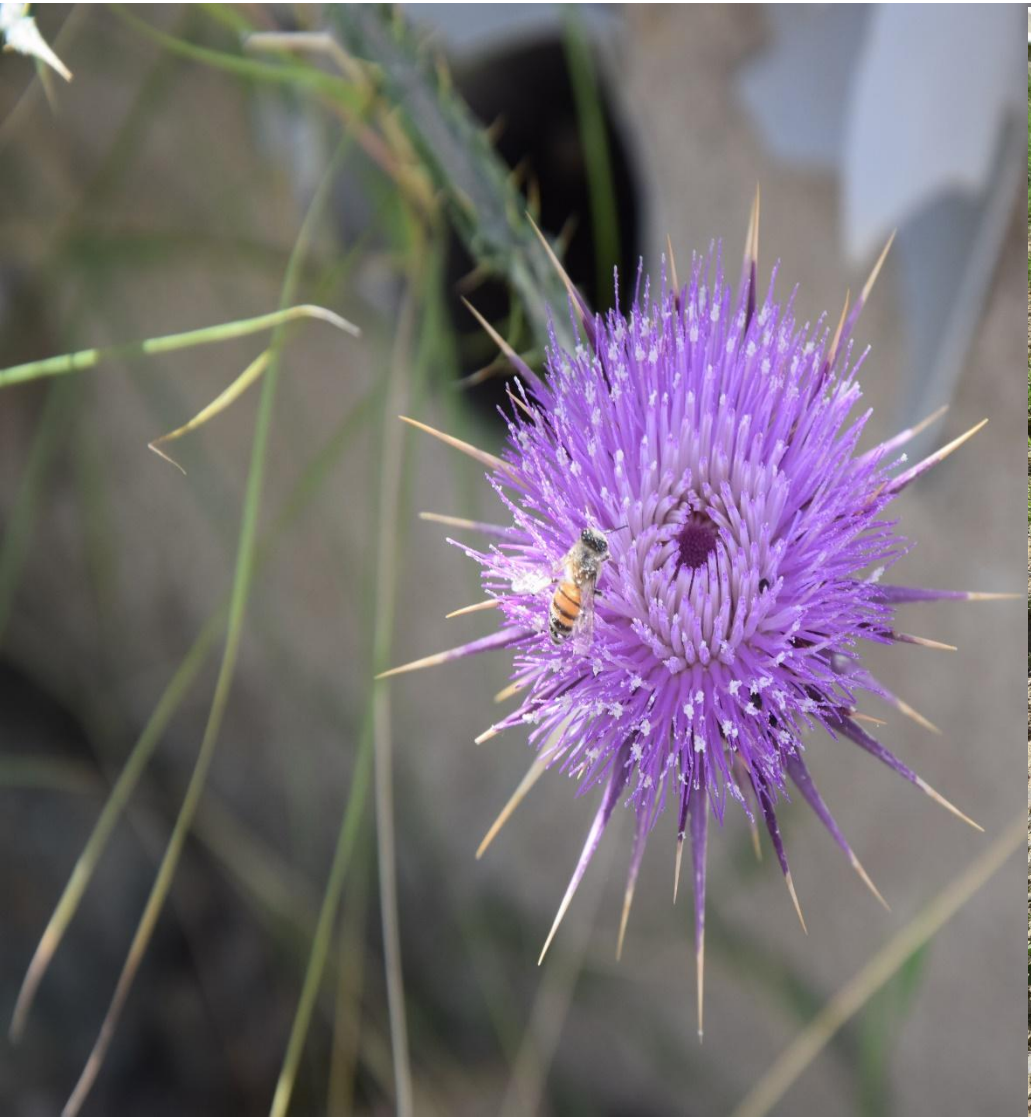

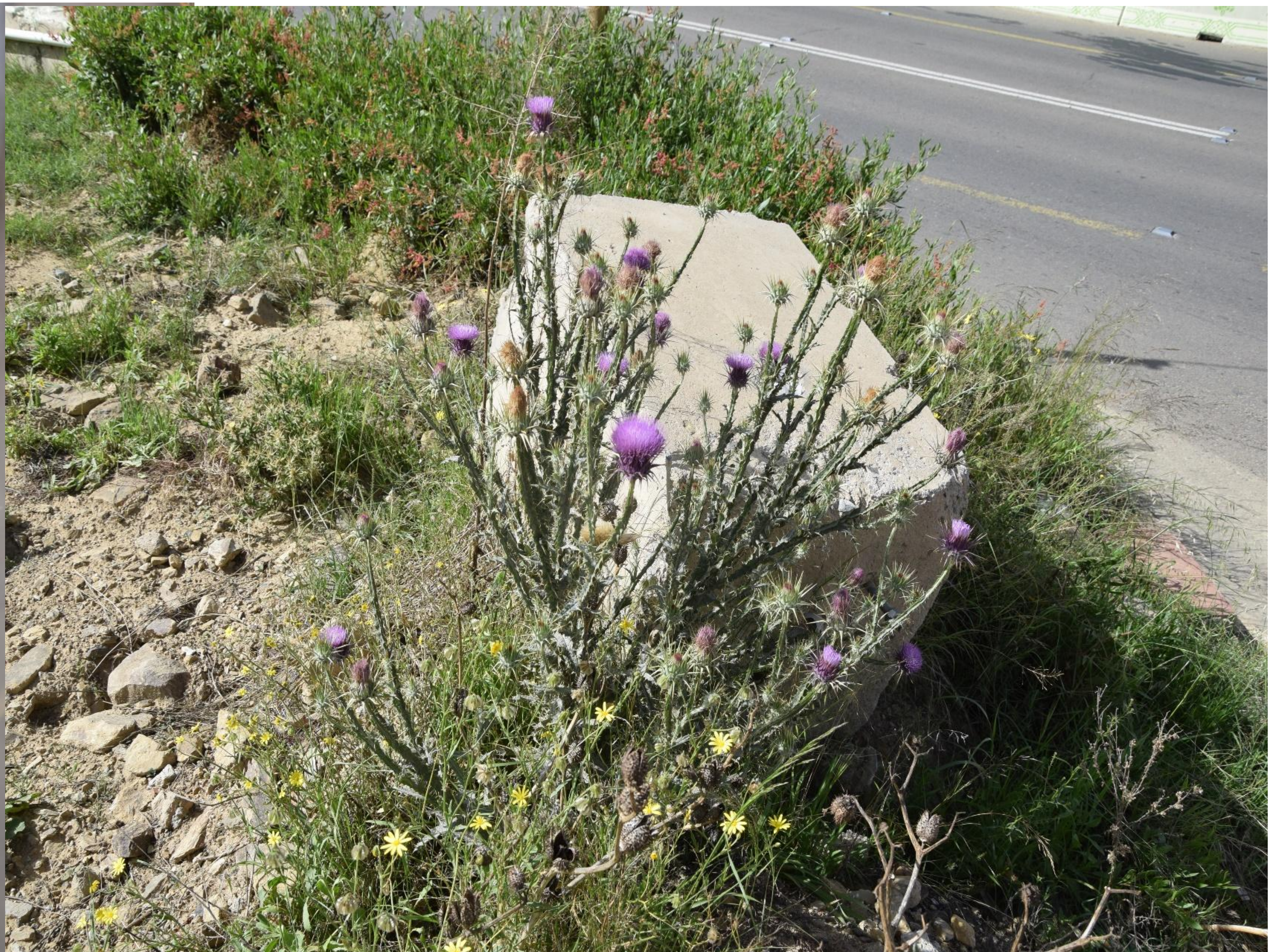

Opuntia ficus indica

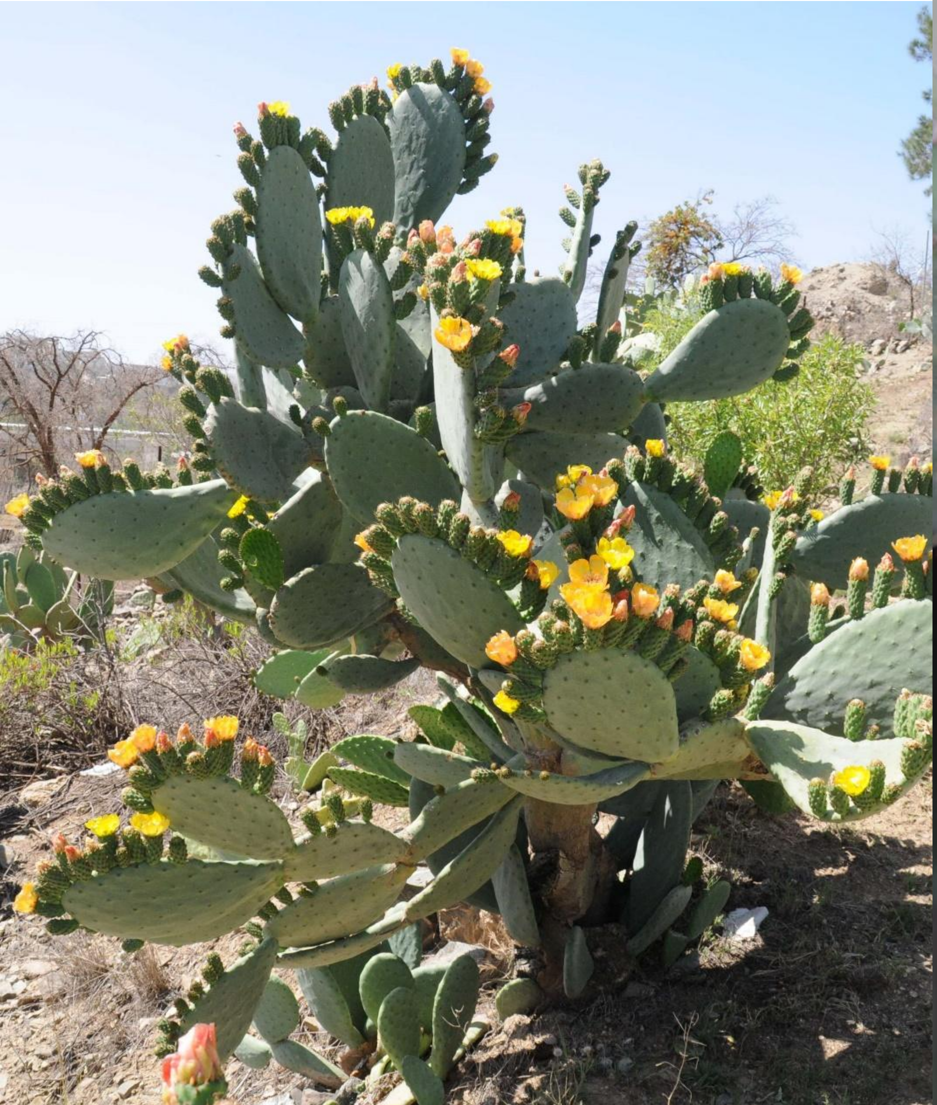

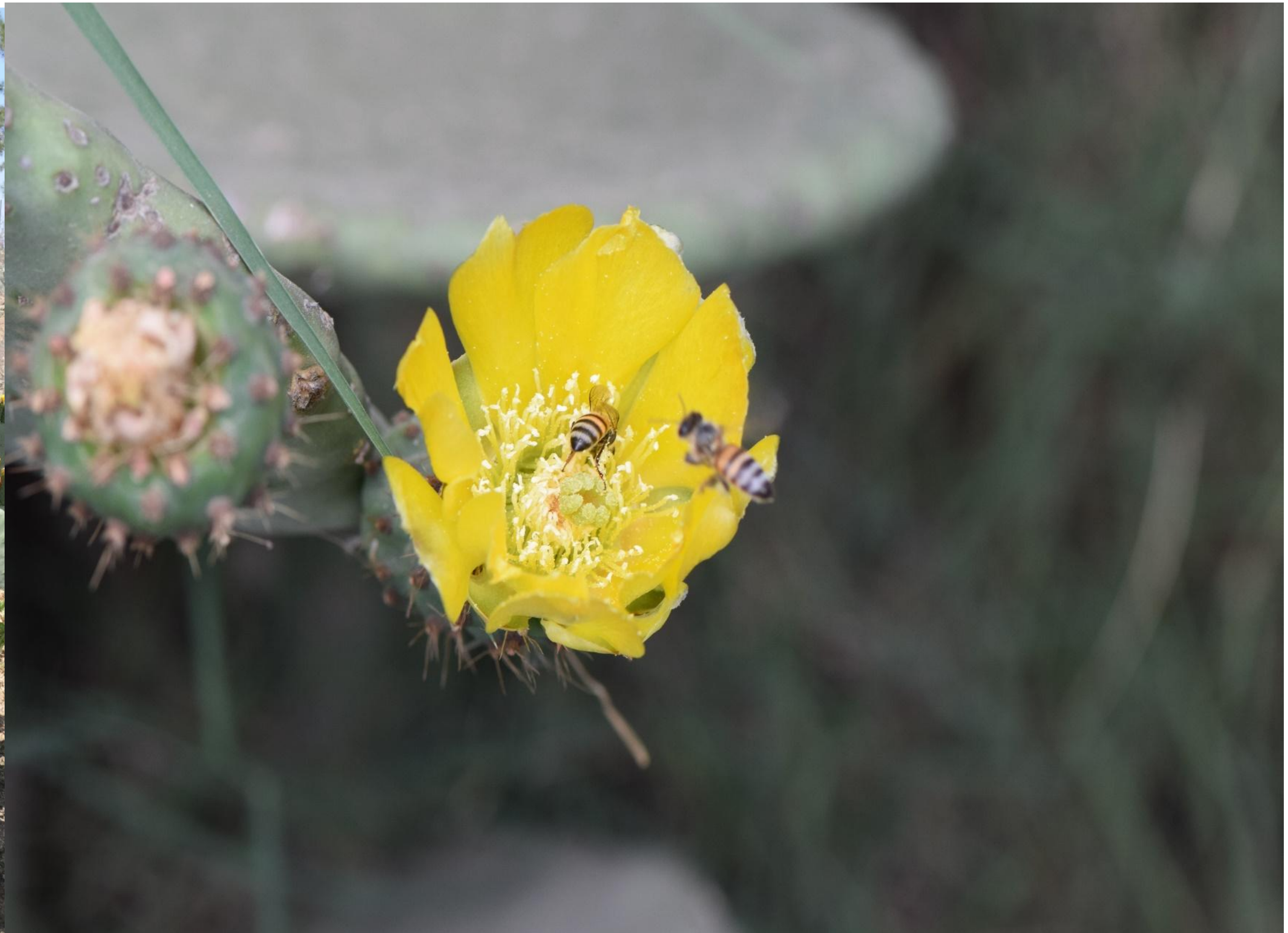

Osteospermum vaillantii

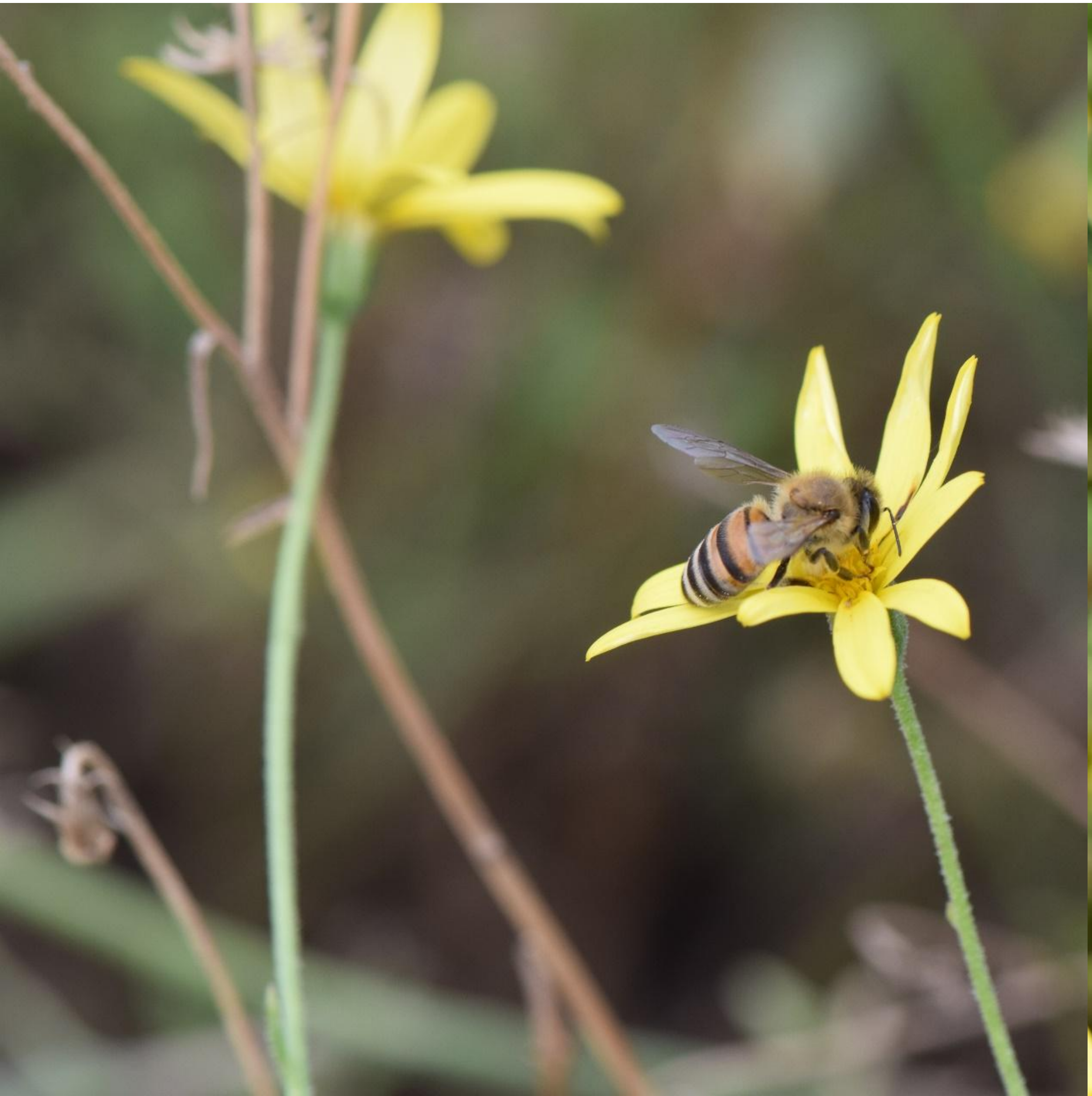

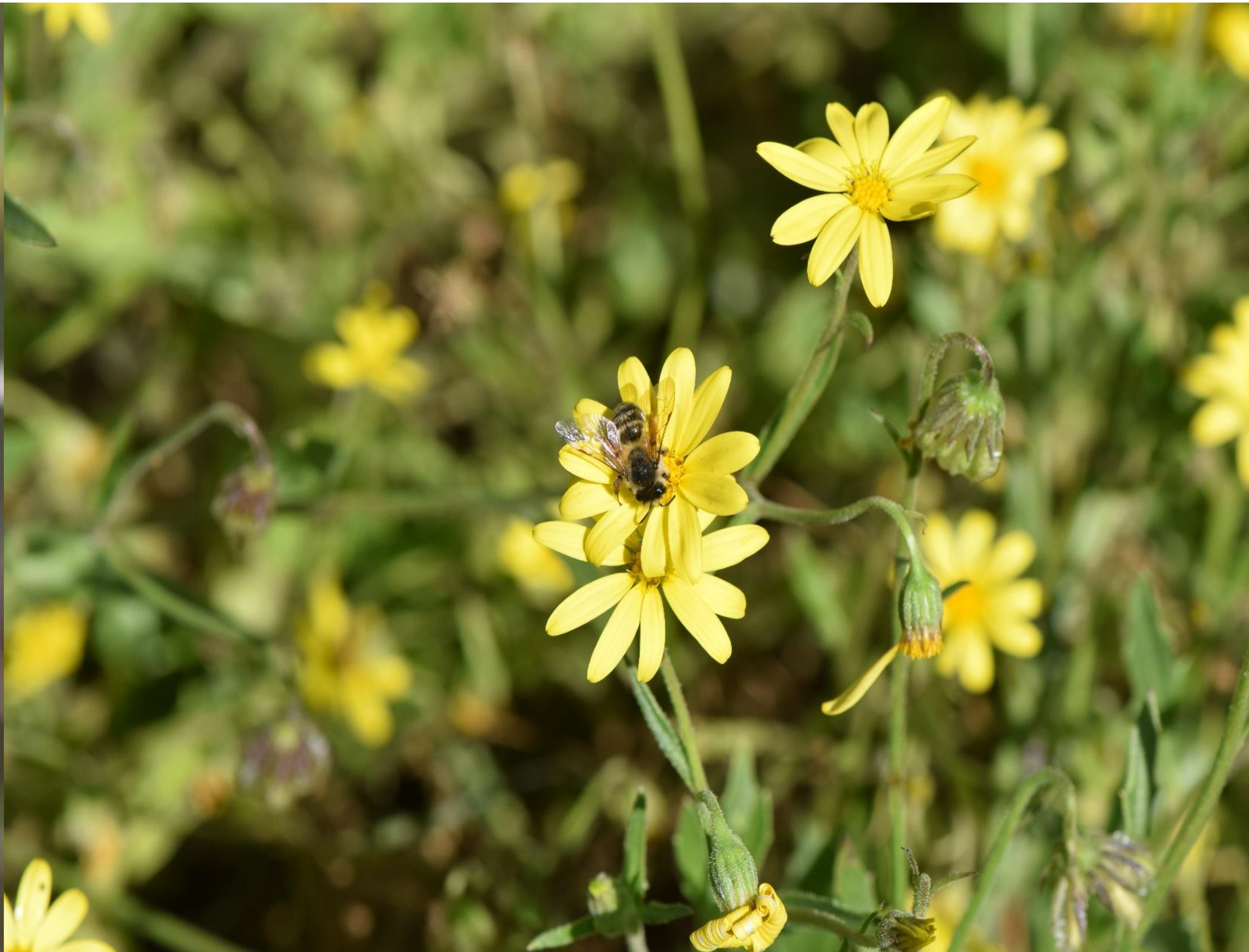

Oxalis corniculata

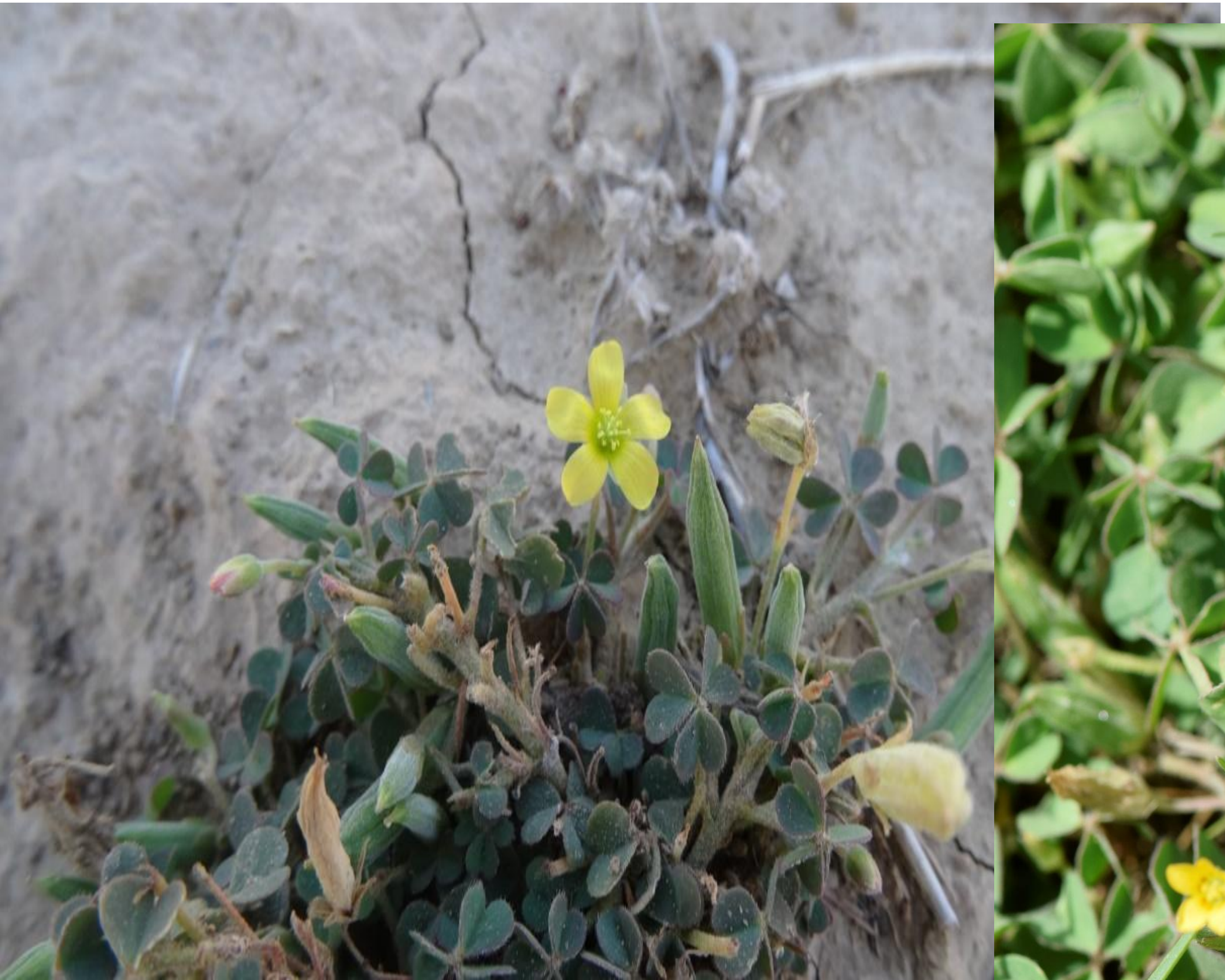

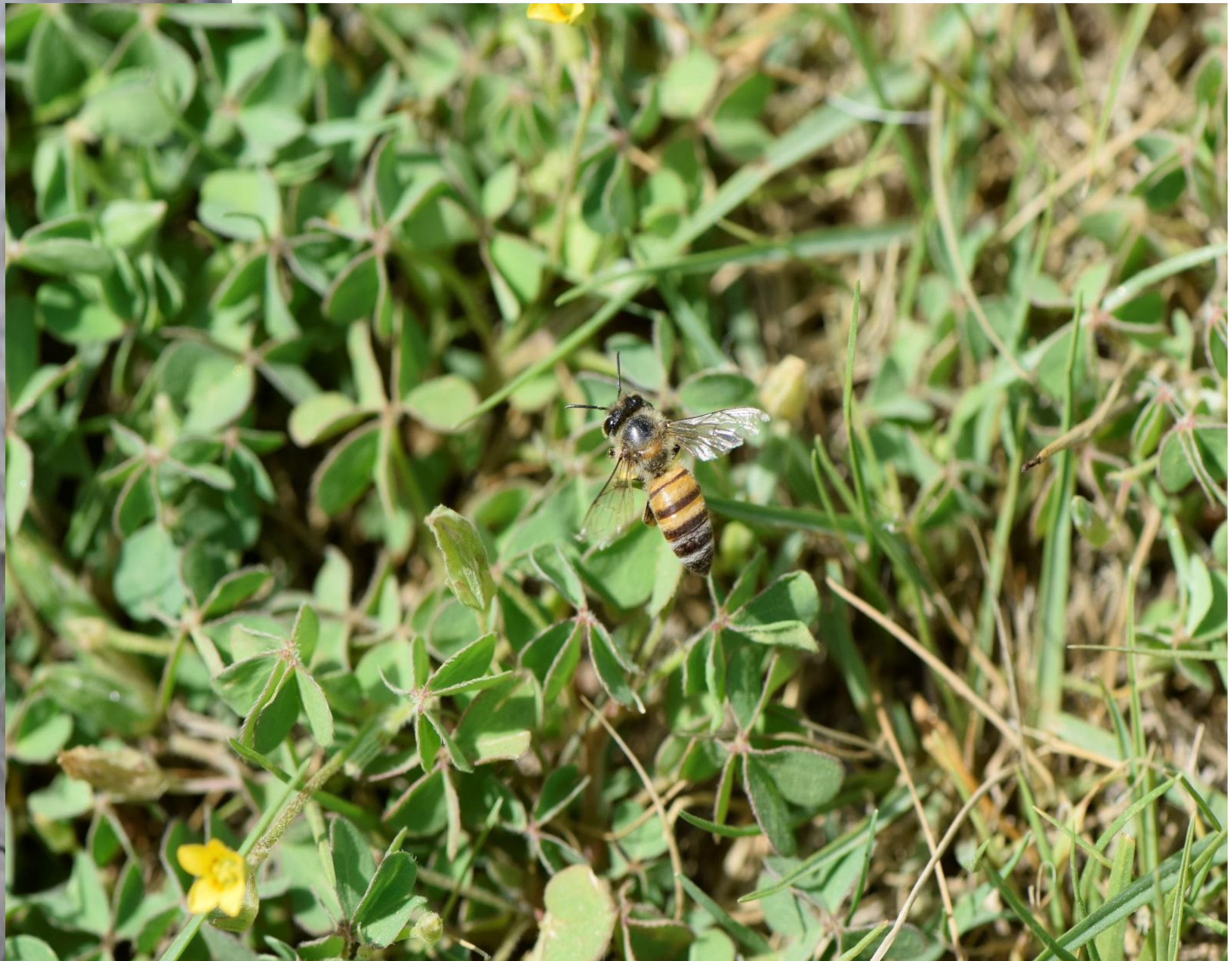

Picris asplenioides

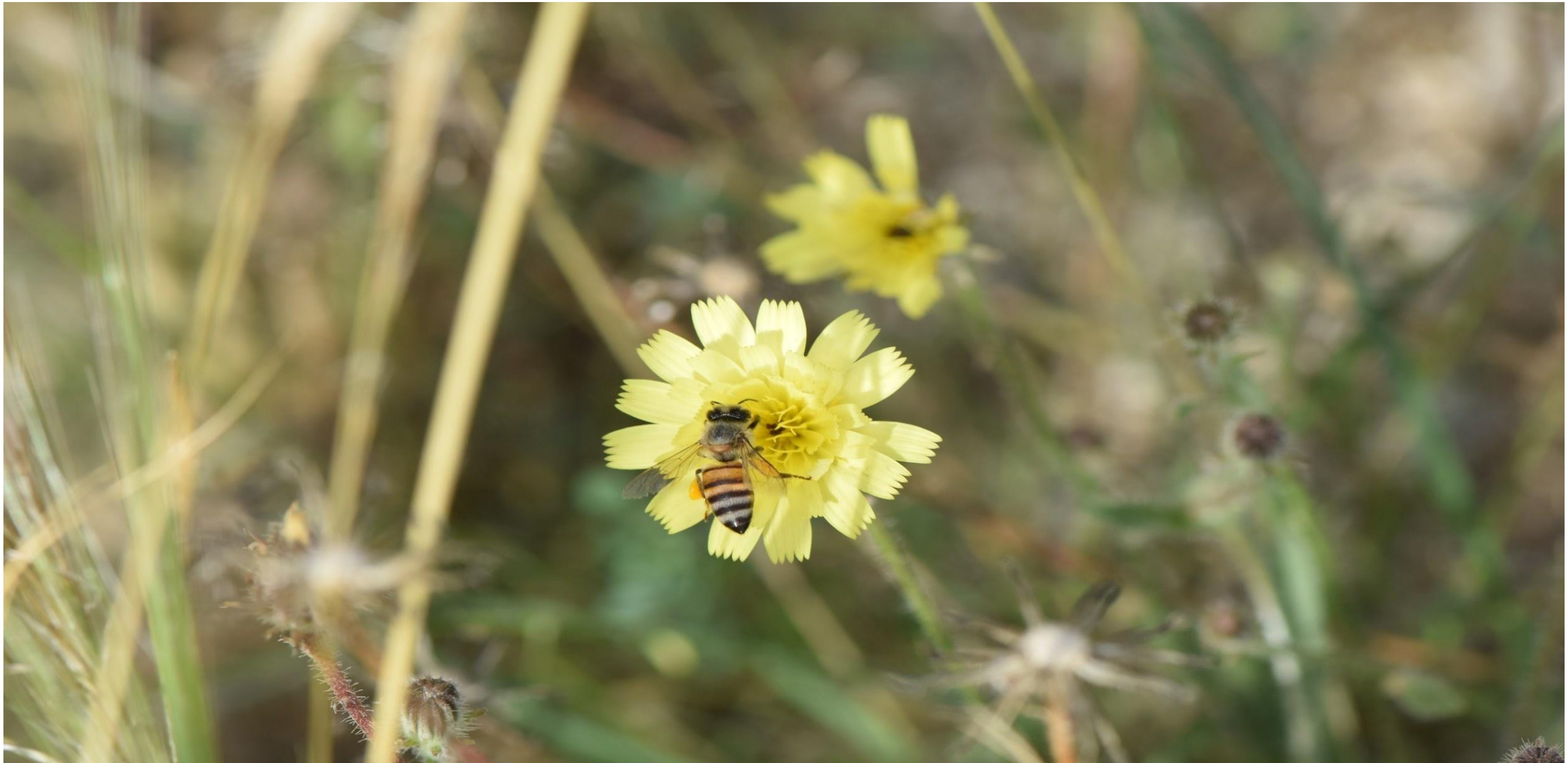

Plantago cylindrica

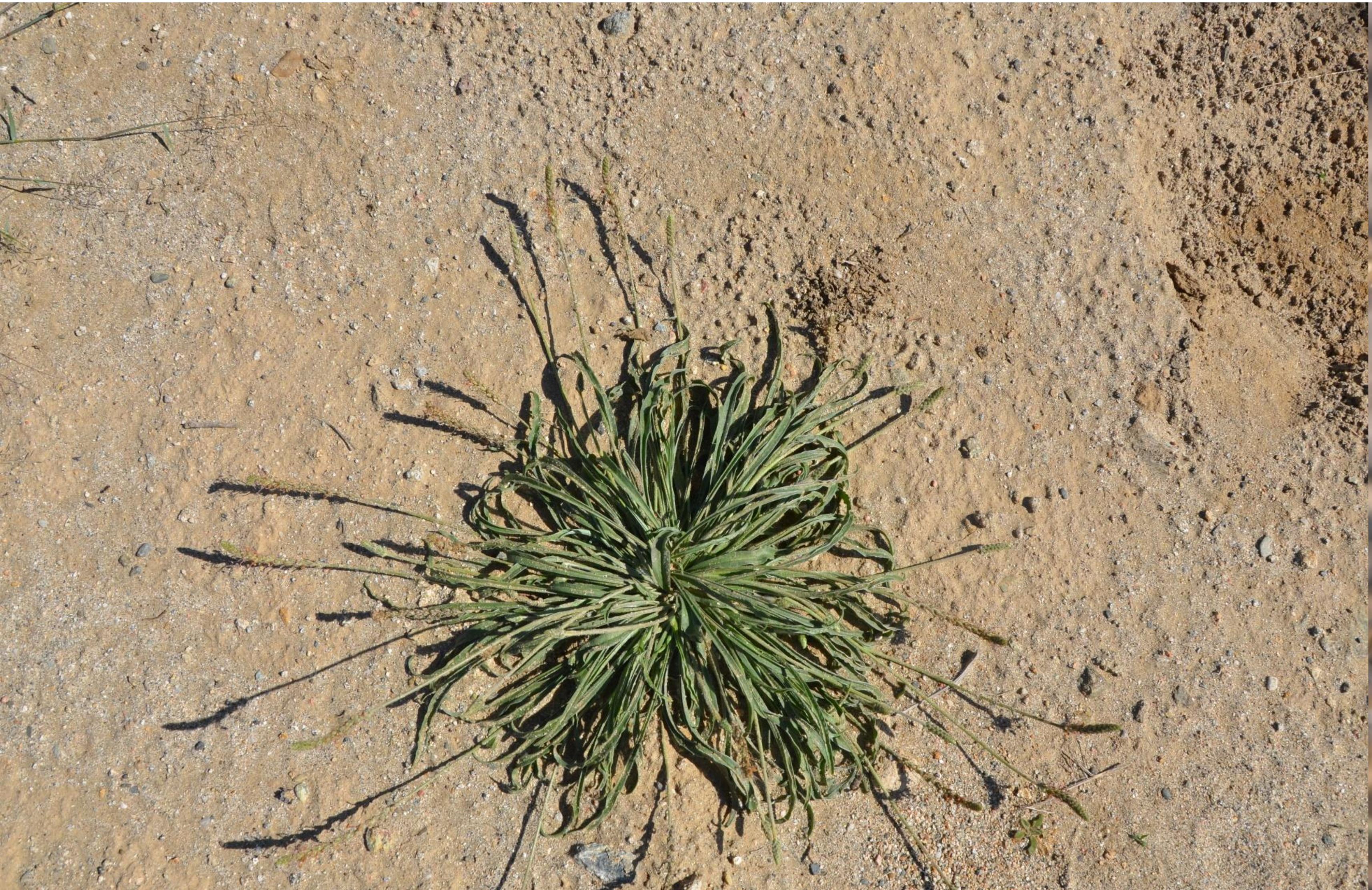

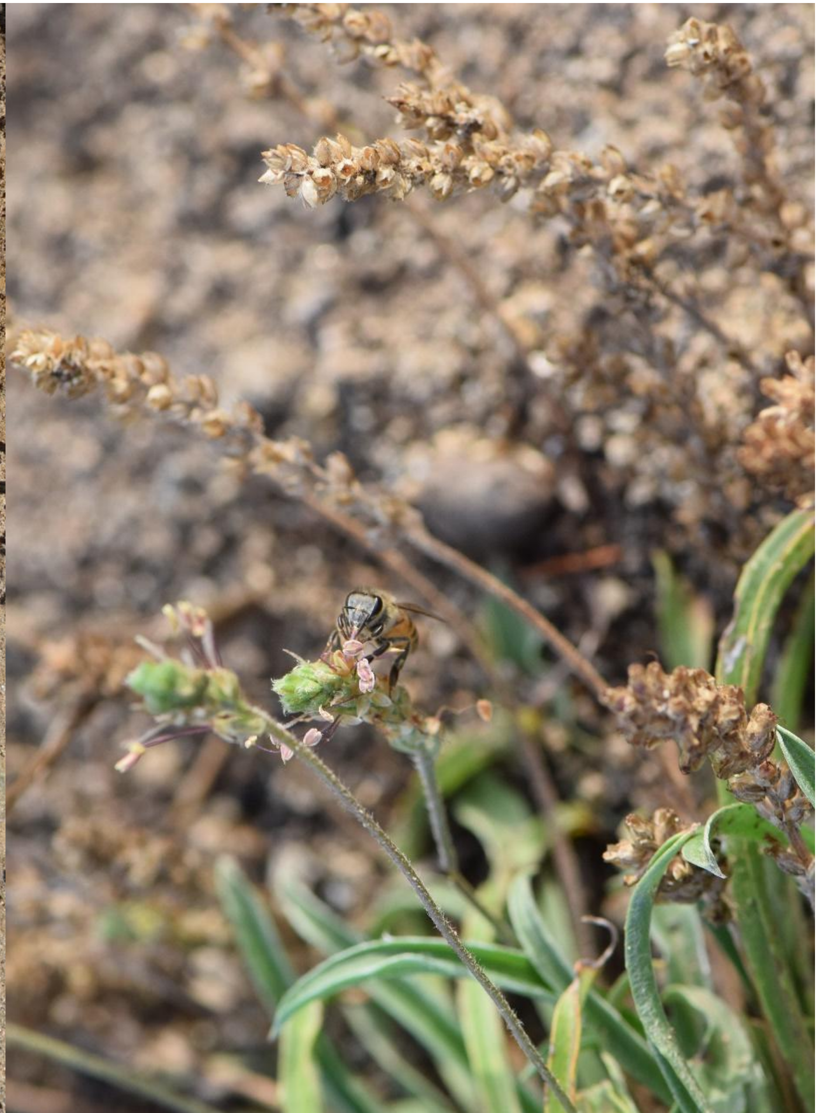

Pluchea dioscoridis

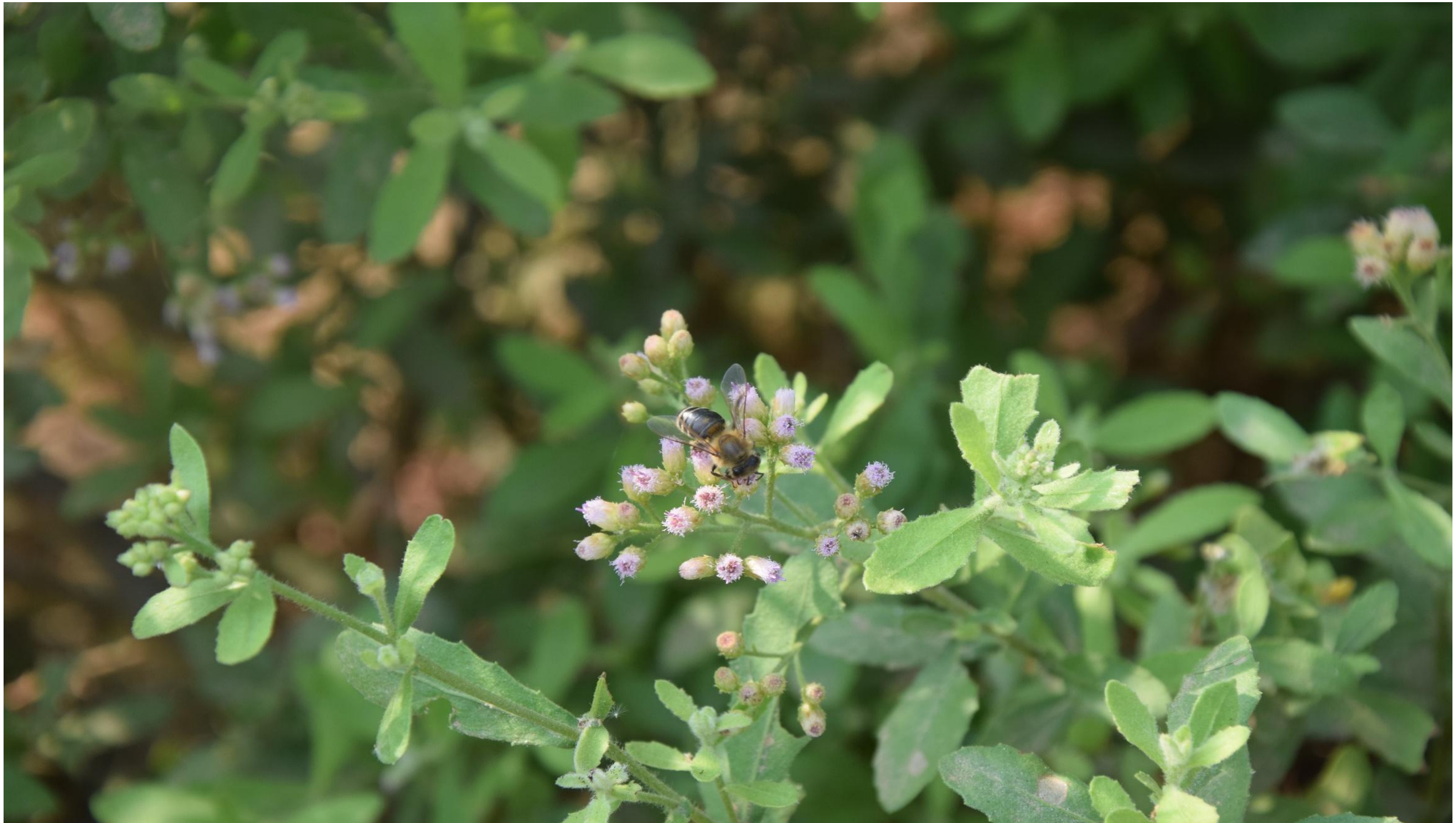

Psiadia punctulata

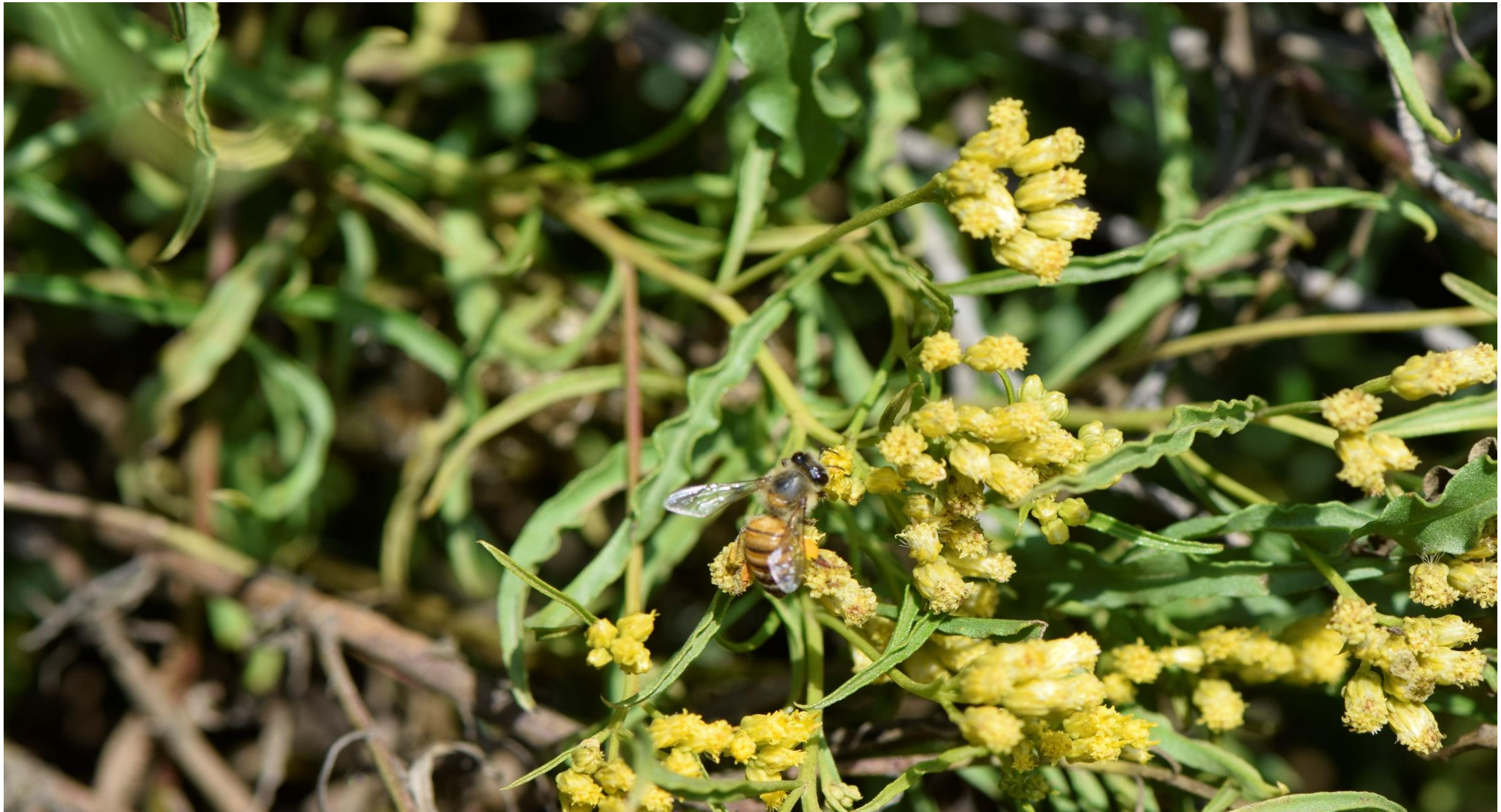

Pyrostria phyllanthoidea

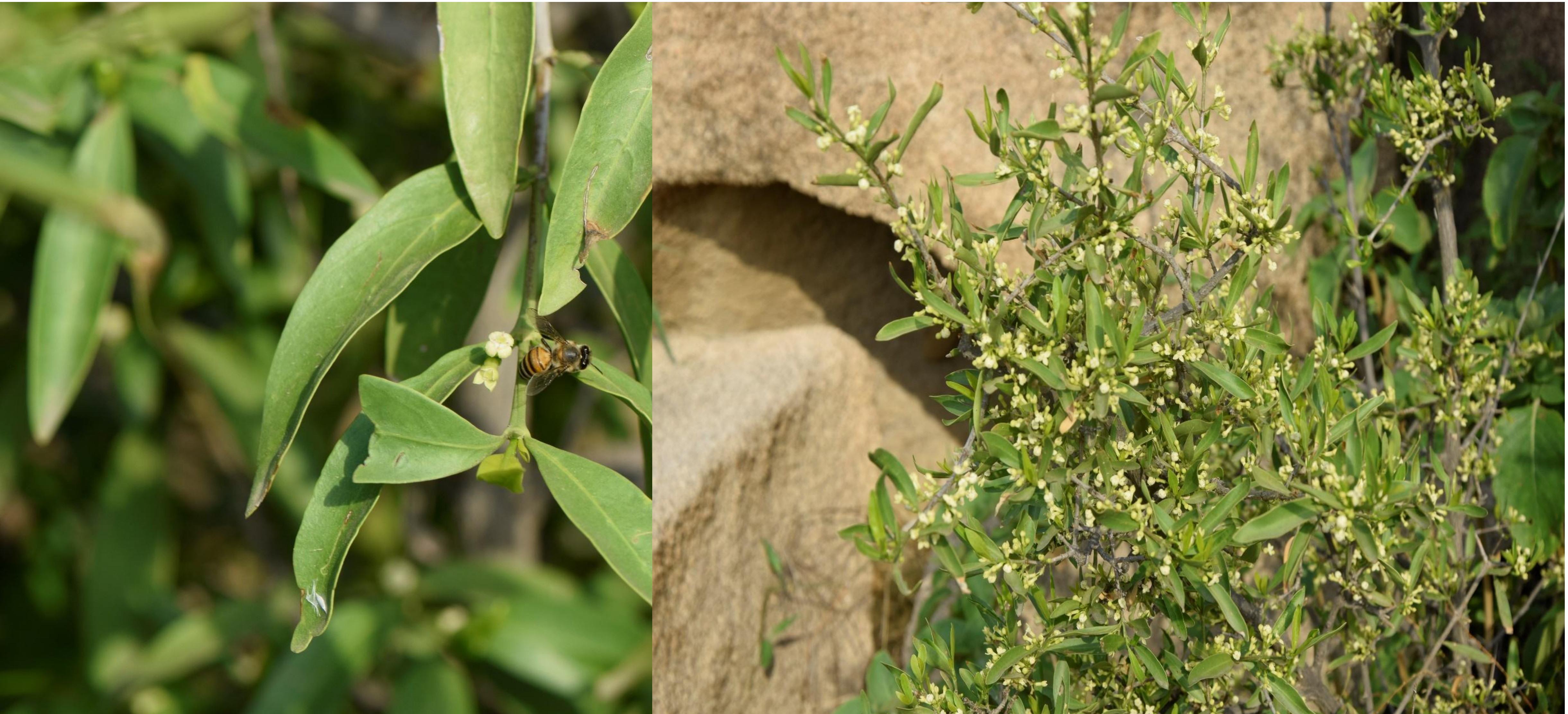

Rosa abyssinica

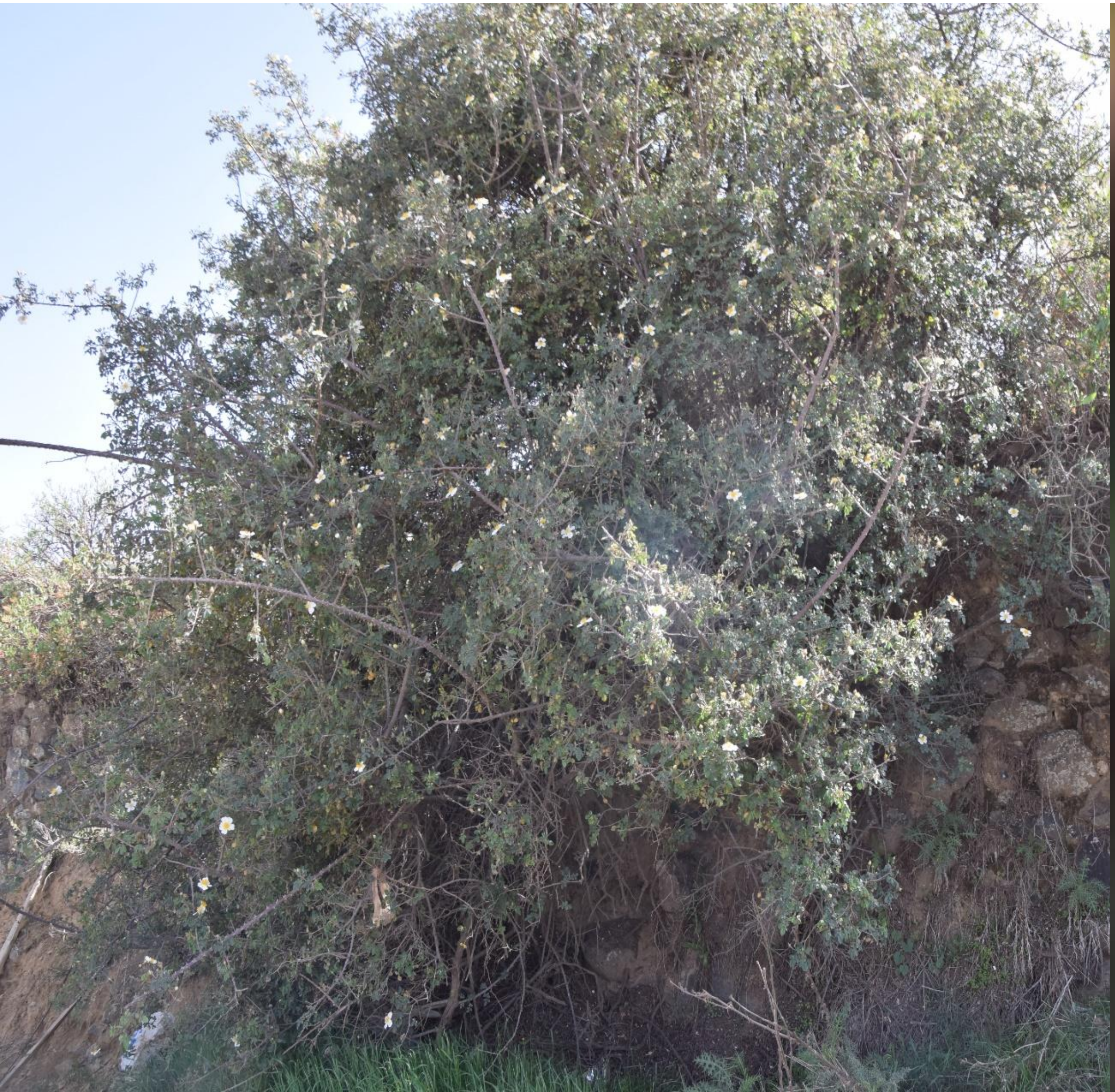

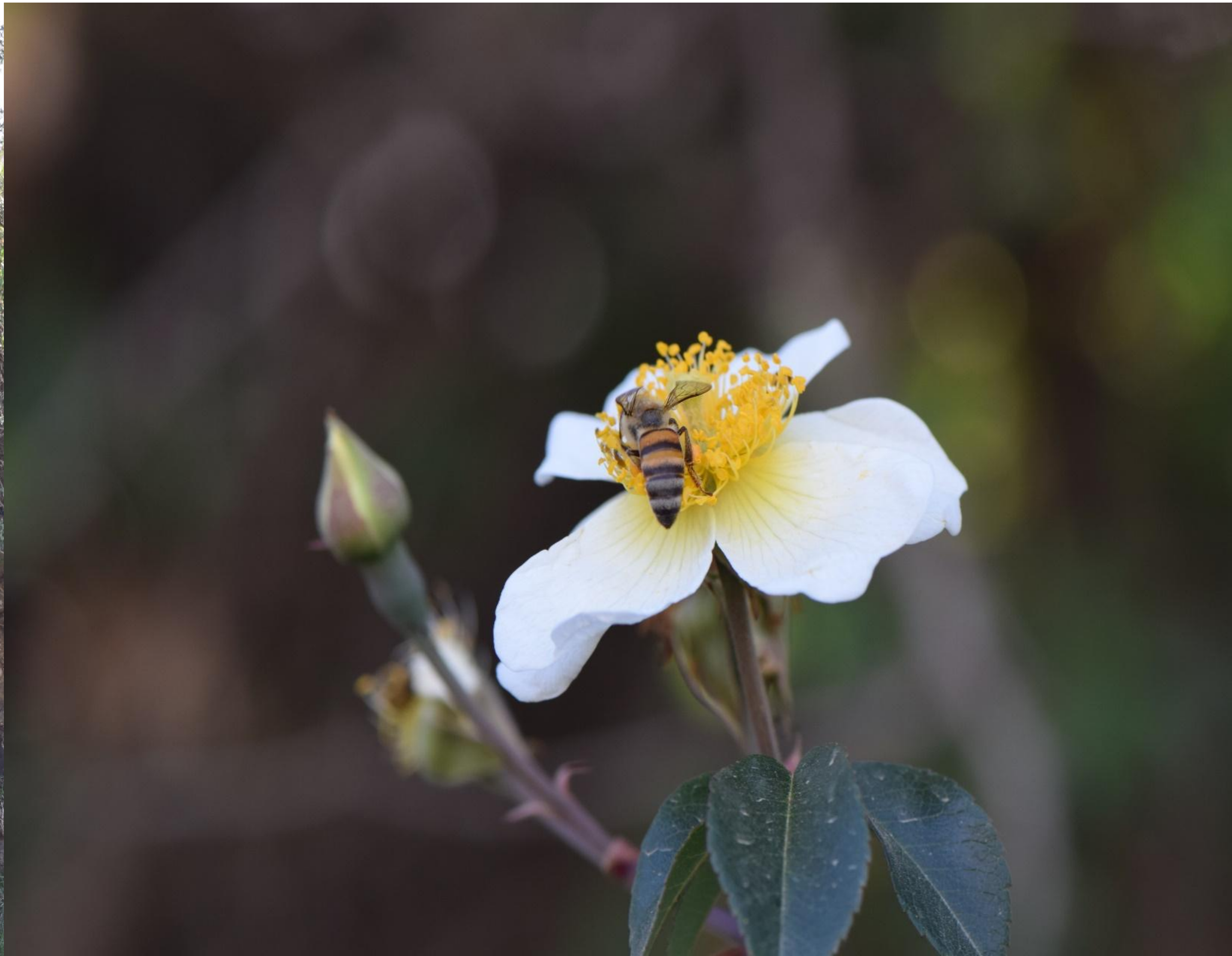

Rubus creticus

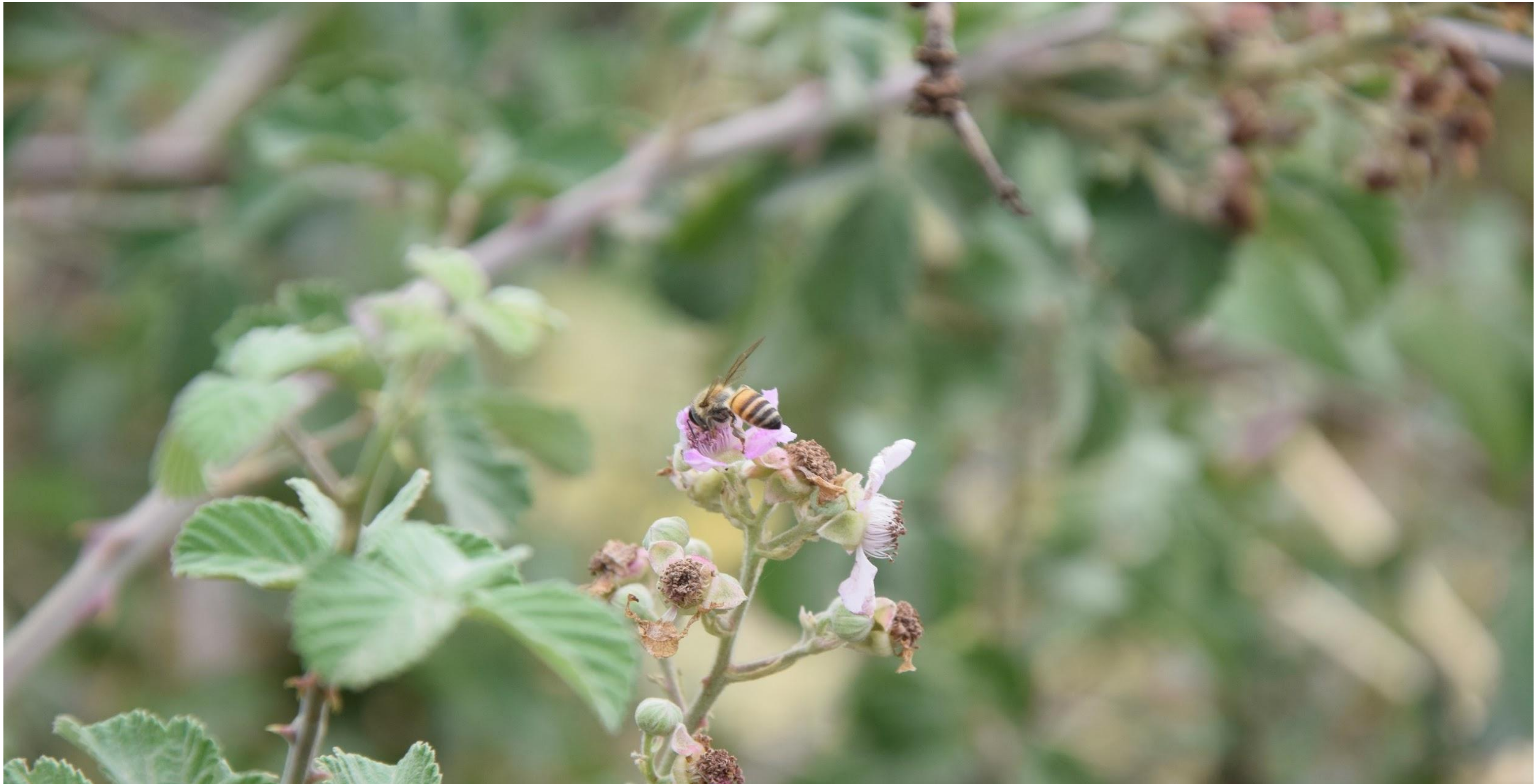

Rumex nervosus

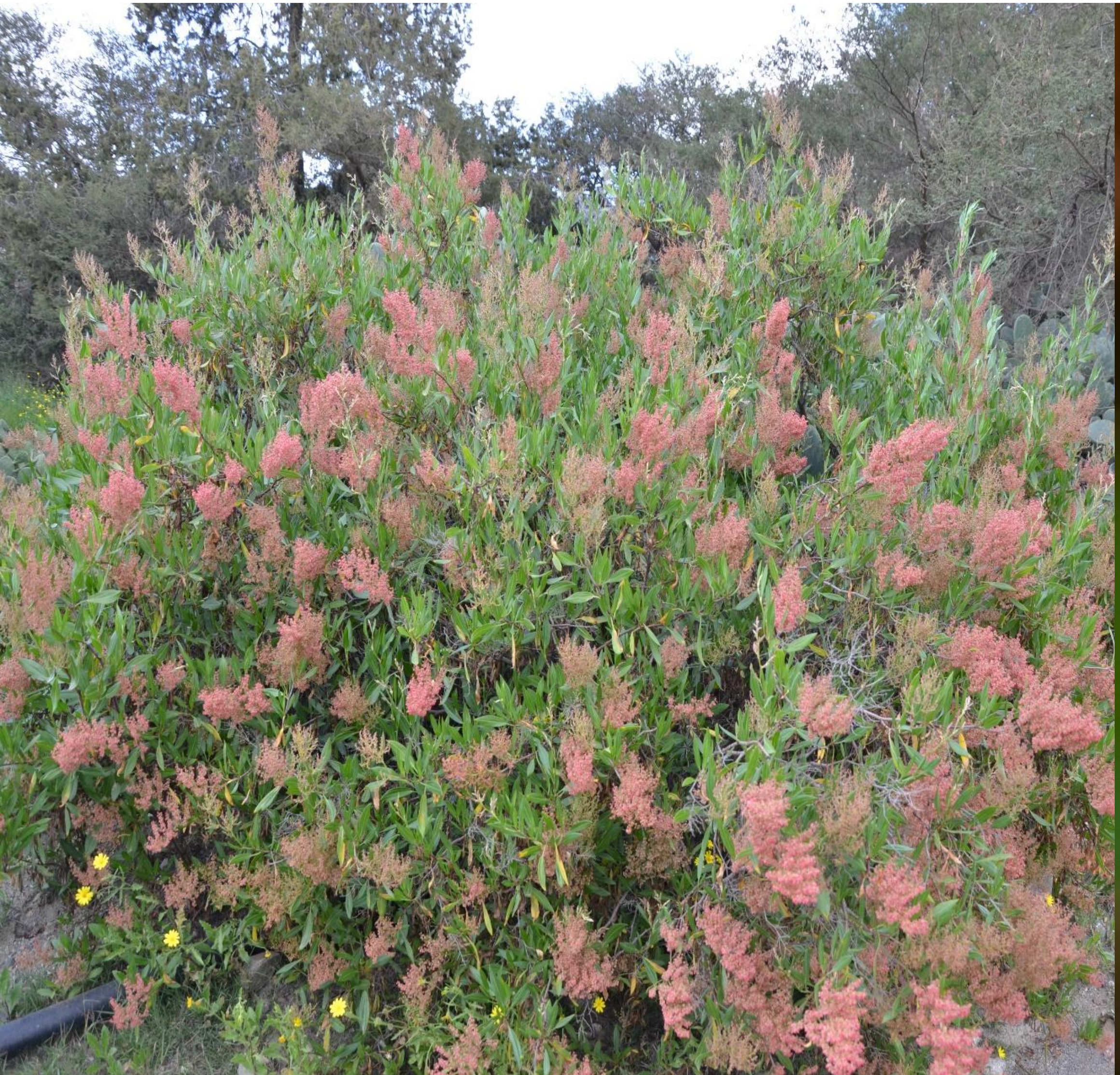

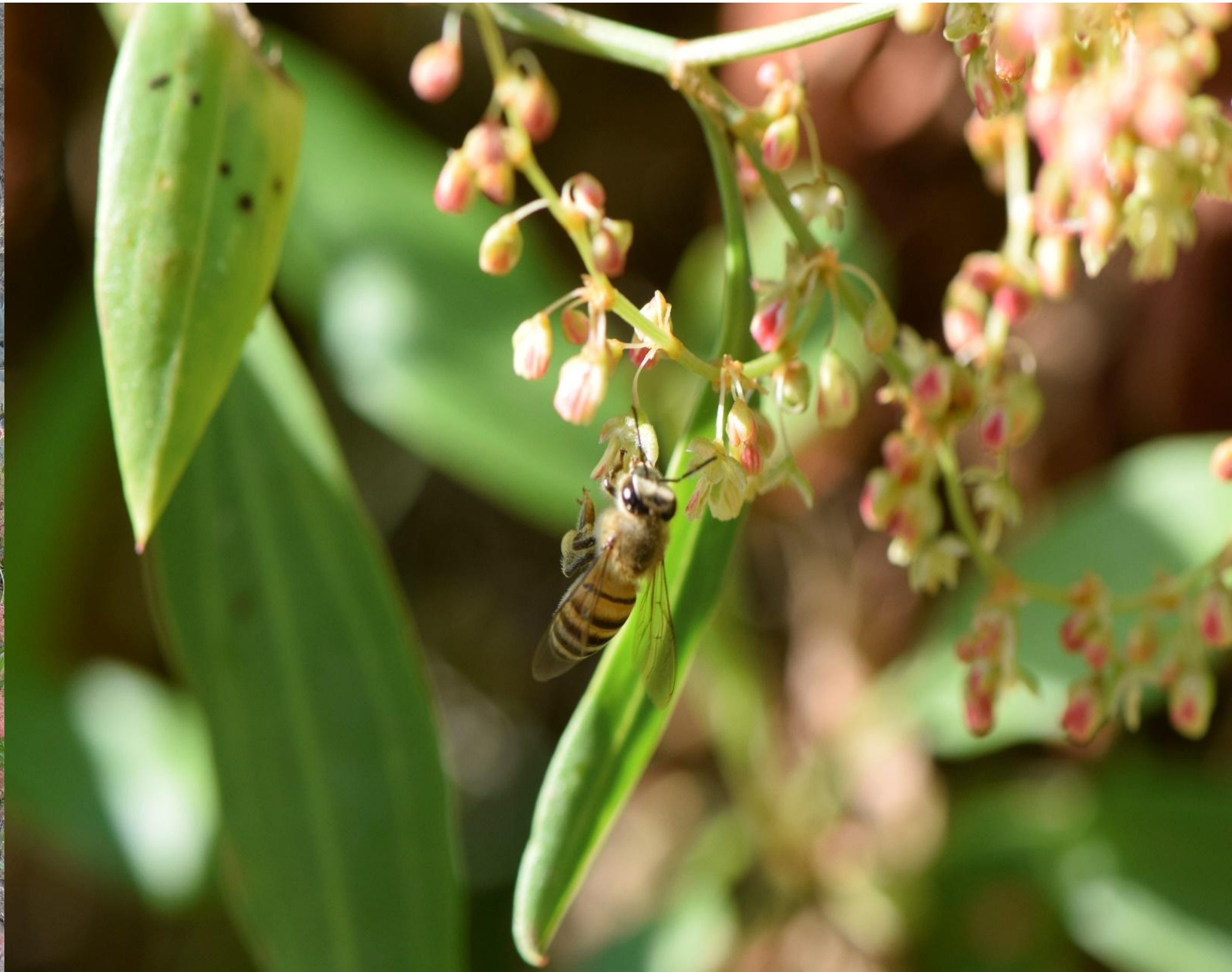

Segeretia thea

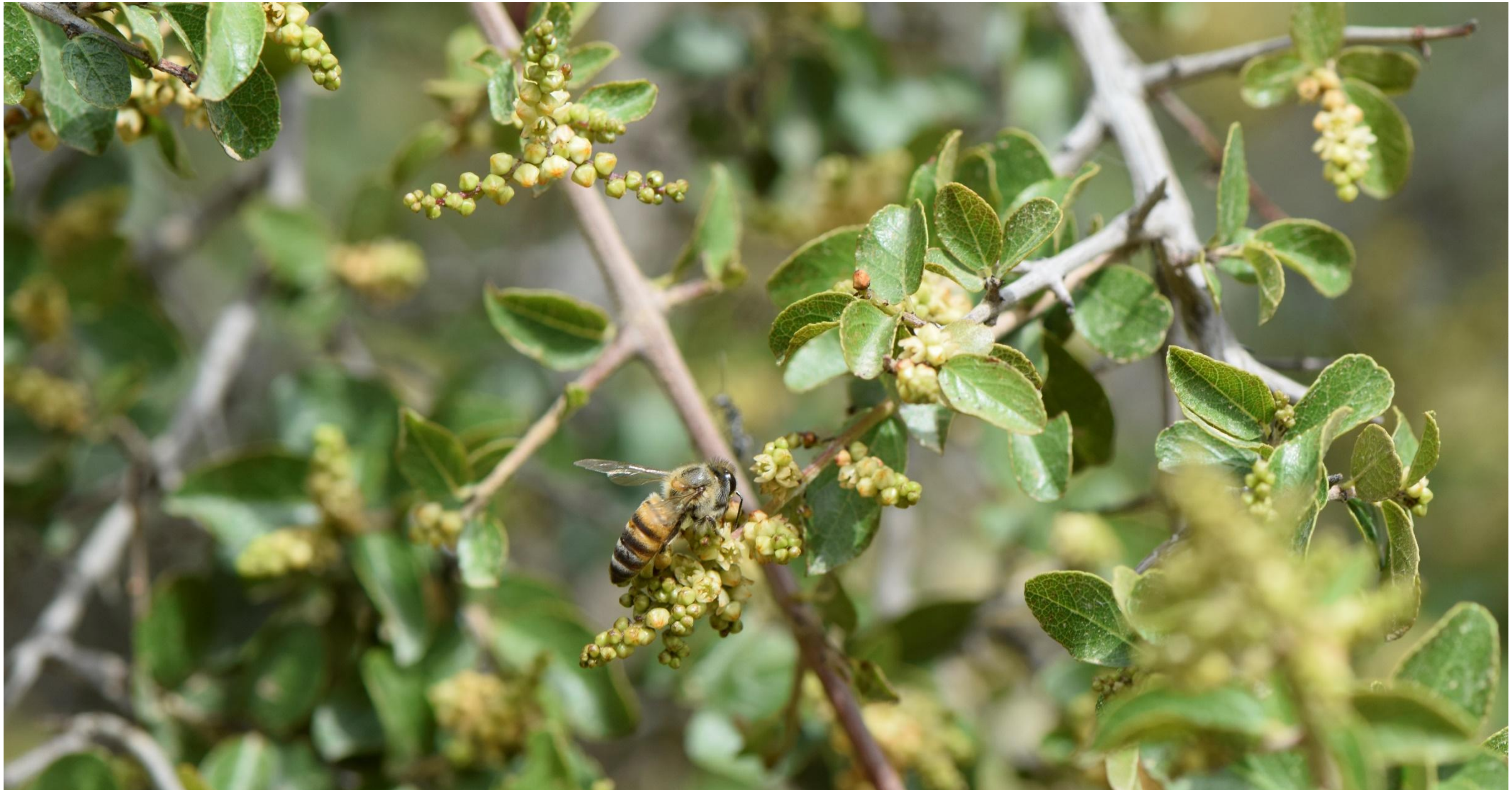

Schweinfurthia pterosperma

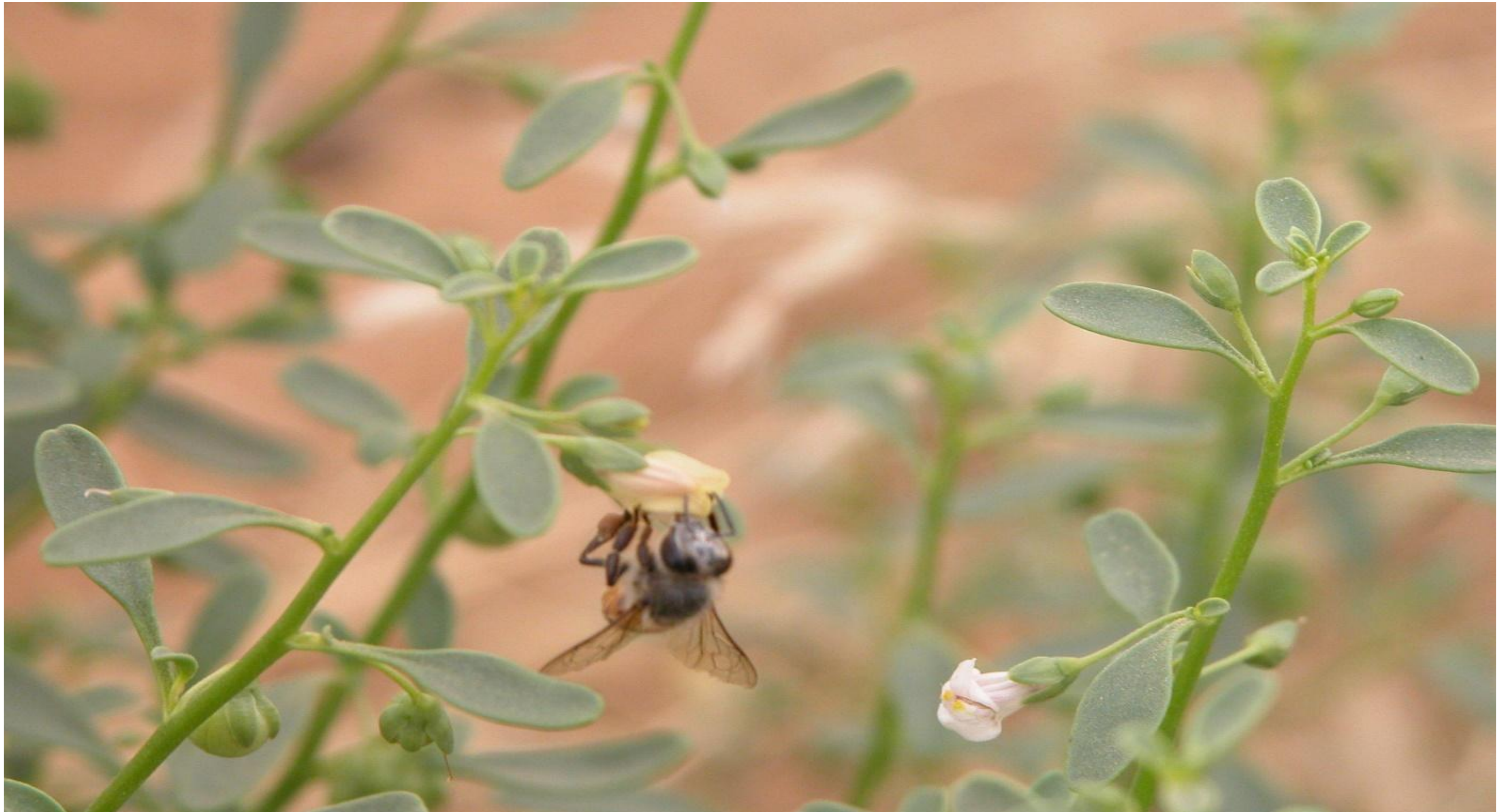

Acacia Oregina

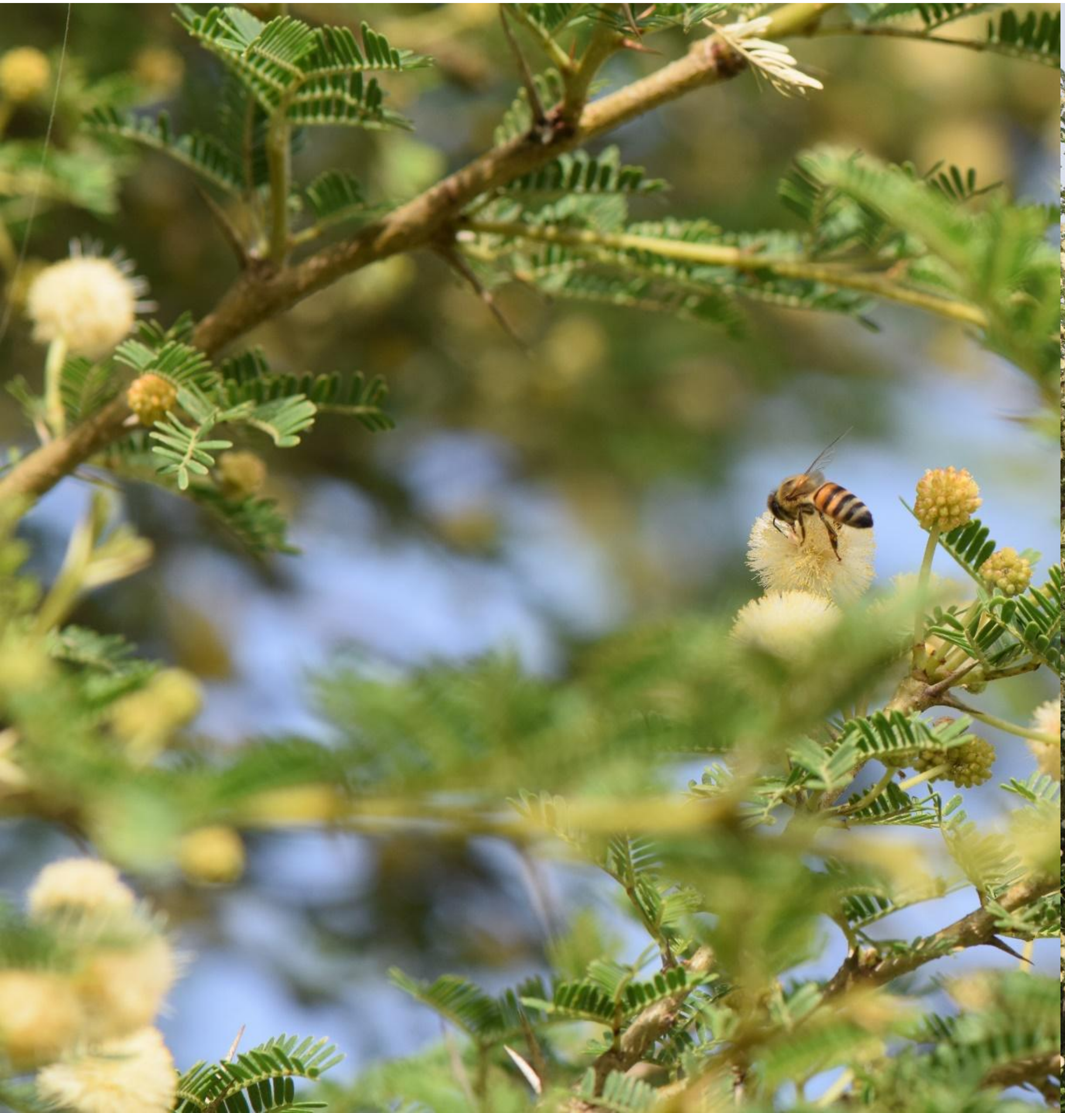

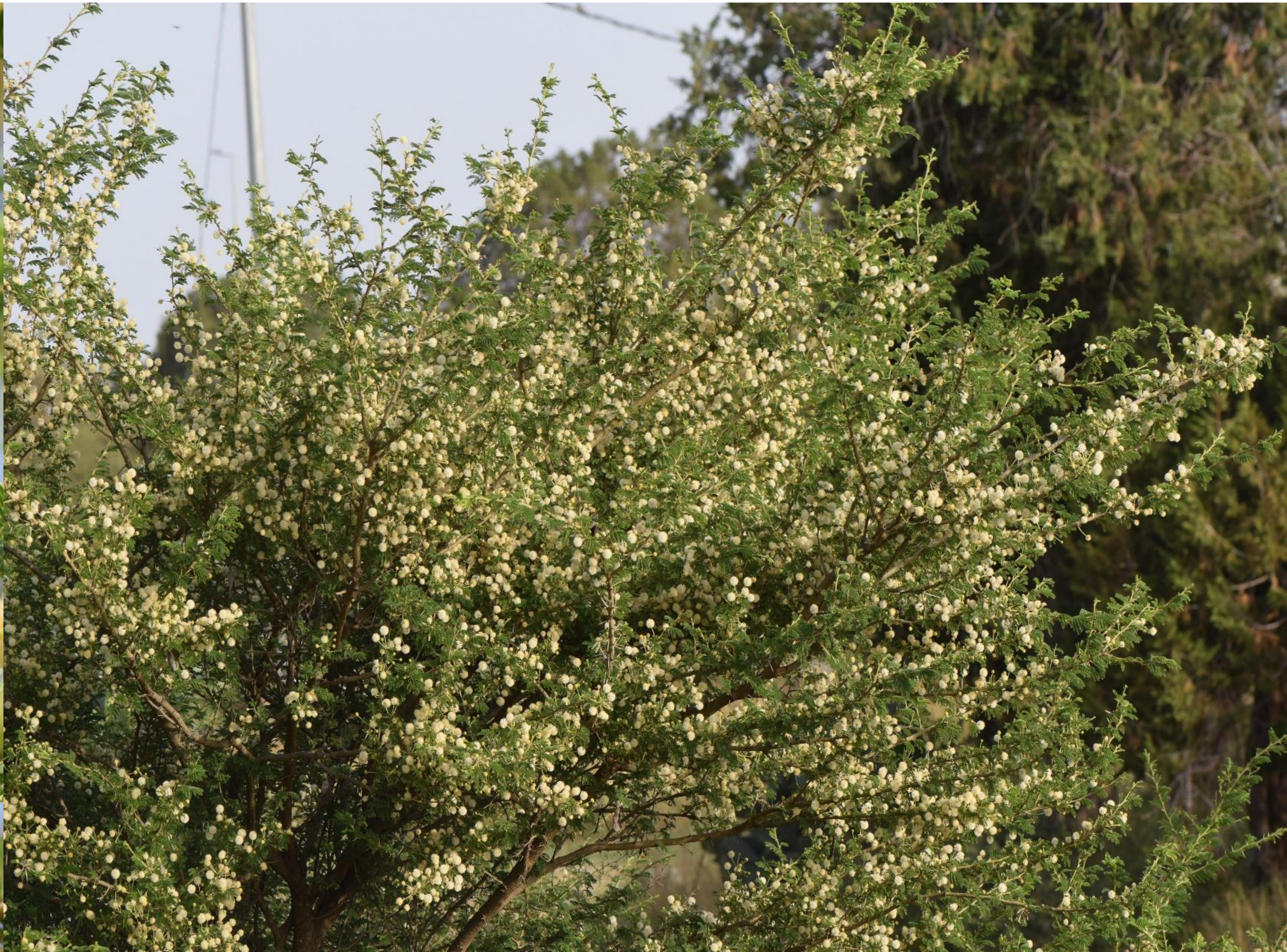

Supplement: Supplementary file 1 [file plants-12-01402-s001.zip › plants-2228482-supplementary.pdf]
